# Bioactive Extracts and Constituents from *Taraxacum mongolicum*: Antioxidant, Anti-Inflammatory, Enzyme-Inhibitory, and Molecular Docking Studies

**DOI:** 10.3390/antiox15060688

**Published:** 2026-05-29

**Authors:** Kuan-Ying Huang, Sin-Min Li, Jih-Jung Chen

**Affiliations:** 1 Department of Pharmacy, School of Pharmaceutical Sciences, National Yang Ming Chiao Tung University, Taipei 112304, Taiwan; kuanying9124.ps13@nycu.edu.tw (K.-Y.H.); sinminli@nycu.edu.tw (S.-M.L.); 2 Department of Medical Research, China Medical University Hospital, China Medical University, Taichung 404333, Taiwan; 3 Traditional Herbal Medicine Research Center, Taipei Medical University Hospital, Taipei 110301, Taiwan

**Keywords:** *Taraxacum mongolicum*, antioxidant, anti-inflammation, molecular docking

## Abstract

*Taraxacum mongolicum*, a medicinal and edible plant of the Asteraceae family, is widely consumed in East Asia and contains diverse bioactive compounds. This study systematically evaluated the bioactivities of whole-plant extracts and their components and elucidated the underlying anti-inflammatory mechanisms. Among the extracts, the methanol fraction exhibited the strongest antioxidant activity, effective inhibition of nitric oxide (NO) production, and modulation of inflammation-related proteins. In addition, the extracts demonstrated α-glucosidase and acetylcholinesterase (AChE) inhibitory activities, indicating multifunctional bioactive potential. Activity-guided analysis identified luteolin (**2**) and apigenin (**4**) as key active compounds with strong NO inhibitory effects. Western blot analysis revealed that both compounds significantly downregulated NO-related protein expression. Mechanistically, luteolin attenuated inflammatory responses by inhibiting NF-κB signaling and modulating the MAPK pathway, whereas apigenin primarily exerted its effects through NF-κB suppression. Both compounds also promoted M2 macrophage marker expression, suggesting a role in immune regulation. Molecular docking analysis further confirmed stable binding interactions of luteolin and apigenin with iNOS and COX-2. Overall, these findings demonstrate that *T. mongolicum* possesses antioxidant, enzyme-inhibitory, and anti-inflammatory activities and supports its further investigation as a multifunctional bioactive resource.

## 1. Introduction

Persistent inflammation and oxidative imbalance are considered key pathogenic mechanisms contributing to a wide range of disorders, such as metabolic abnormalities, progressive neurodegenerative diseases, immune system dysfunction, and aging-related physiological deterioration. Extensive evidence indicates that sustained inflammatory imbalance triggers overproduction of reactive oxygen species. Oxidative stress, in turn, amplifies inflammatory signaling, forming a self-perpetuating vicious cycle that disrupts cellular homeostasis, exacerbates tissue injury, and accelerates disease progression [1,2,3].

Nitric oxide (NO) functions as a critical mediator of immune responses and acts as an inflammatory mediator while also connecting inflammatory signaling with oxidative stress [4]. NO readily reacts with reactive oxygen species (ROS), particularly superoxide anions (O_2_^•−^), to generate highly reactive nitrogen species (RNS) [5,6]. These reactive intermediates induce oxidative and nitrate reduction in essential cellular components, thereby impairing normal cellular activity and exacerbating tissue injury [7]. In chronic inflammation-associated conditions, including atherosclerosis, neurodegenerative disorders, and metabolic pathologies, sustained overproduction of NO frequently coexists with elevated ROS levels [8,9]. This imbalance is considered a major driver of nitro-oxidative stress and contributes to persistent inflammatory activation and disease progression [10].

Although conventional anti-inflammatory therapies and corticosteroids are effective in controlling acute inflammatory responses, their long-term use in chronic disease settings is often limited by adverse gastrointestinal, cardiovascular, and immunological effects [11]. Consequently, there is growing interest in identifying alternative strategies with improved safety profiles that can simultaneously modulate multiple inflammation- and oxidative stress-related signaling pathways. Under these circumstances, plant-derived compounds and natural products are receiving growing attention owing to their multitarget biological activities, low toxicity profiles, and potential suitability for prolonged use. Numerous studies have demonstrated that polyphenols and flavonoids play an important role in regulating cellular redox homeostasis and inflammatory signaling pathways, including those mediated by NF-κB and MAPK, supporting their immunomodulatory and anti-inflammatory activities [12].

*Taraxacum mongolicum* Hand.-Mazz., a medicinal–edible plant in the Asteraceae family, has been traditionally consumed and used across East Asia and the temperate regions of Eurasia. With growing interest in plant-derived bioactive compounds and natural functional ingredients, *Taraxacum mongolicum* has increasingly been recognized as a plant-based bioresource with considerable development potential. *T. mongolicum* has emerged as a promising botanical resource. The safety of dandelion for human consumption has been recognized by organizations including EFSA and the U.S. NCCIH, particularly when used in herbal products and dietary supplements. Furthermore, dandelion extracts have been designated as Generally Recognized as Safe (GRAS) by the U.S. Food and Drug Administration (FDA), supporting their incorporation into health-related formulations [13,14]. These regulatory approvals provide a robust safety framework for further biomedical investigation of *Taraxacum mongolicum* derived bioactive compounds.

Earlier studies have demonstrated that ethanolic and aqueous extracts of *Taraxacum mongolicum* exhibit anti-inflammatory and antioxidant activities [15,16]. However, most existing studies have focused on individual extracts or isolated biological endpoints, and systematic investigations integrating solvent-dependent chemical composition, multifunctional bioactivities, and underlying molecular mechanisms remain limited. Although significant progress has been made in the discovery of plant-derived bioactive compounds, many phytochemicals remain insufficiently characterized and require further investigation [17,18,19,20,21,22,23,24,25]. To address these limitations, this study employed an activity-guided, multi-level experimental strategy to systematically evaluate the bioactive potential of whole-plant *Taraxacum mongolicum* extracts in inflammation- and immune-related contexts. By integrating solvent fractionation across different polarities, enzyme- and cell-based assays, compound isolation and structural characterization, and mechanistic analyses, this study aims to elucidate the molecular basis underlying the immunomodulatory properties of *T. mongolicum* and to provide a basis for future in vivo and translational investigation.

## 2. Materials and Methods

### 2.1. Chemical and Reagent

All solvents and chemical reagents employed in this study were of analytical or reagent grade and were used as received without additional purification. These materials were purchased from several commercial suppliers, including Sigma-Aldrich (St. Louis, MO, USA), Alfa Aesar (Lancashire, UK), Acros Organics (Geel, Belgium), ChemFaces, Chemicals and reagents were purchased from MedChemExpress (Monmouth Junction, NJ, USA), SHOWA Chemical Co., Ltd. (Chuo-ku, Japan), Tokyo Chemical Industry Co., Ltd. (Tokyo, Japan), and Avantor Performance Materials (Radnor, PA, USA). Spectroscopic analyses elucidated the structures of the isolated compounds. Proton nuclear magnetic resonance (^1^H-NMR) spectra were obtained on a Bruker 400 MHz spectrometer. Chemical shift values (δ) are reported in parts per million (ppm), and coupling constants (*J*) are expressed in hertz (Hz). Signal multiplicities were described using standard abbreviations, including s for singlet, d for doublet, t for triplet, q for quartet, m for multiplet, and br for broad signals. Infrared spectra were recorded using a Shimadzu IRAffinity-1S Fourier-transform infrared spectrophotometer (IR). Analytical thin-layer chromatography (TLC) and preparative thin-layer chromatography (PTLC) were conducted on silica gel 60 F254 plates purchased from Merck (Darmstadt, Germany). Detailed spectroscopic information for all isolated compounds, including ^1^H-NMR, electrospray ionization mass spectrometry (ESI-MS), and IR data, is provided in the Supporting Information.

### 2.2. Plant Material and Identification

The dried whole plant of *Taraxacum mongolicum*, consisting of roots, stems, leaves, and flowers, was purchased in July 2024 from Wenchang Traditional Chinese Medicine Pharmacy (Nangang District, Taipei, Taiwan). Plant identification was carried out by Prof. Jih-Jung Chen, based on the voucher specimen (HAST 126585) has been deposited in the Herbarium of the Biodiversity Research Center, Academia Sinica (Taipei, Taiwan). The dried plant sample underwent stepwise extraction with a series of solvents arranged according to increasing polarity, namely *n*-hexane, dichloromethane, ethyl acetate, acetone, ethanol, methanol, distilled water, and water (100 °C). Each solvent extraction was performed by shaking the plant material with the corresponding solvent for 72 h at 25 °C. After extraction, the suspensions were filtered to remove solid plant residues, and the resulting filtrates were evaporated under reduced pressure to eliminate the solvents. The crude extracts obtained from each solvent were dried and kept at −20 °C before subsequent analyses.

### 2.3. Isolation and Characterization of Compounds from Taraxacum mongolicum

**Figure 1:**
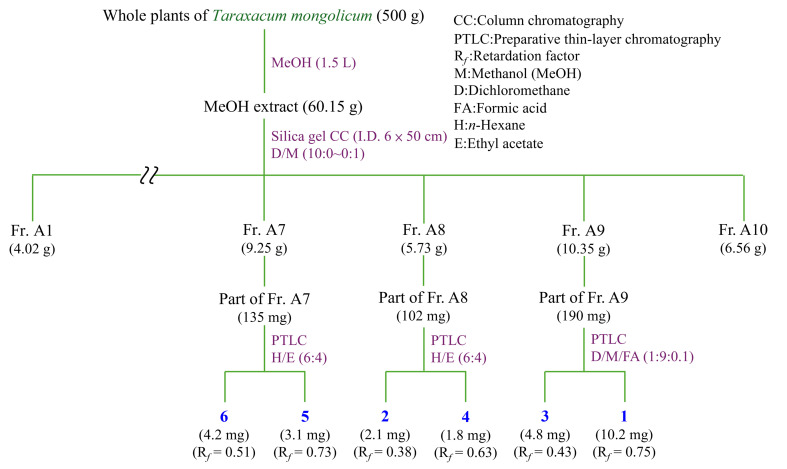
Extraction and isolation of active components from *Taraxacum mongolicum*.

Dried and powdered whole plants of *Taraxacum mongolicum* (500 g) were extracted with methanol (1500 mL) at 25 °C for 24 h. After extraction, the solutions were combined, filtered, and the solvent was removed under reduced pressure, resulting in a dark brown methanolic crude extract weighing 60.15 g. The crude extract was subsequently subjected to silica gel column chromatography (I.D. 6 × 50 cm). Elution was performed using a gradient system of D/M (10:0–0:1, *v*/*v*), which afforded ten fractions (Fr. 1–10) (Figure 1).

Part (135 mg) of fraction A7 was further purified by preparative thin-layer chromatography (PTLC) using H/E = 6:4, yielding compound **5** (3.1 mg, R*_f_* = 0.73), and **6** (4.2 mg, R*_f_* = 0.51). Part (102 mg) of fraction A8 (102 mg) was purified by PTLC with H/E = 6:4, yielding the constituents **2** (2.1 mg, R*_f_* = 0.38), **4** (1.8 mg, R*_f_* = 0.63), part of fraction A9 (190 mg) was purified by PTLC with D/M/FA = 10:90:1, getting components **1** (10.2 mg, R*_f_* = 0.75) and **3** (4.8 mg, R*_f_* = 0.43).

**Figure 2:**
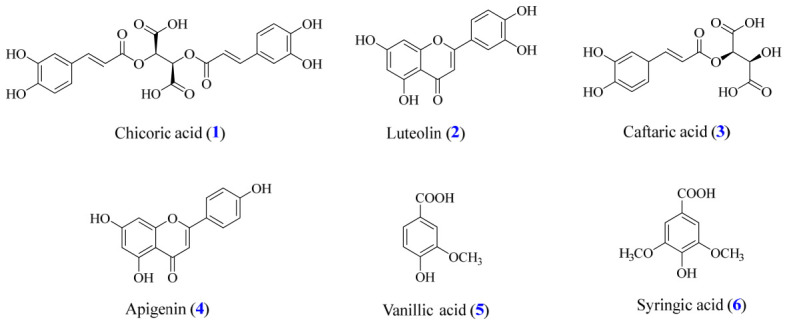
Chemical structure of Chicoric acid (**1**), Luteolin (**2**), Caftaric acid (**3**), Apigenin (**4**), Vanillic acid (**5**), Syringic acid (**6**) from *Taraxacum mongolicum*.

The six isolated functional constituents (Figure 2) were identified by comparing their ^1^H NMR, IR, and ESI–MS spectral data with authentic standards and published literature values. Based on these comparisons, the functional constituents were determined as chicoric acid (**1**) [26], luteolin (**2**) [27], caftaric acid (**3**) [28], apigenin (**4**) [29], vanillic acid (**5**) [30], and syringic acid (**6**) [31]. The purity of the isolated compounds was evaluated by high-performance liquid chromatography (HPLC). HPLC analysis of compounds **1**–**6** was performed using a gradient elution method. The mobile phase conditions were as follows: 6% acetonitrile (ACN) containing 0.1% formic acid (FA) from 0 to 3 min; 20% ACN containing 0.1% FA from 3 to 25 min; a linear gradient from 20% to 95% ACN containing 0.1% FA from 25 to 32 min; 95% to 6% ACN containing 0.1% FA from 32 to 35 min; and 6% ACN containing 0.1% FA from 35 to 40 min. The stationary phase consisted of a C18 column (250 mm × 4.6 mm, 5 μm). The UV detection wavelength was set at 254 nm, the flow rate was maintained at 1 mL/min, and the column temperature was kept at 25 °C. In the HPLC analyses, the purities of compounds 1–6 were all higher than 95.0%. Specifically, compound **1** showed a purity of 97.9% at a retention time (*t*_R_) of 13.23 min; compound **2**, 97.72% at *t*_R_ = 30.08 min; compound **3**, 97.96% at *t*_R_ = 6.61 min; compound **4,** 97.11% at *t*_R_ = 30.70 min; compound **5**, 97.01% at *t*_R_ = 8.37 min; and compound **6**, 97.64% at *t*_R_ = 8.33 min. Their spectroscopic data and HPLC purity analysis results of compounds **1**–**6** were provided in the Supporting Information (Figures S1–S24).

### 2.4. Total Flavonoid Content (TFC)

The total amount of flavonoids of each extract was determined through the AlCl_3_ colorimetric reaction, which forms stable flavonoid–aluminum complexes detectable at 415 nm [32]. Methanol diluted samples were reacted with 10% AlCl_3_ and 0.1 mM potassium acetate for 30 min at 25 °C. Absorbance was measured, and TFC was expressed as mg QE/g extract using a quercetin calibration curve.

### 2.5. Quantification of Total Phenolic Content (TPC)

The Folin–Ciocalteu colorimetric assay was used to estimate the TPC of each extract [32]. The absorbance was read at 750 nm using an ELISA microplate reader, and results were expressed as mg GAE per g extract based on a gallic acid standard curve.

### 2.6. Evaluation of DPPH Radical Scavenging Capacity

The DPPH radical-scavenging activity was evaluated using a published protocol [33]. An ethanolic DPPH solution (400 μM) was mixed 1:1 with the test solution (100 μL each; samples prepared by diluting DMSO stocks with ethanol). After standing in the dark at 25 °C for 30 min, the absorbance of the mixtures was determined at 520 nm. Scavenging (%) was calculated as:

Scavenging activity (%) = [(A_0_ − A_1_)/A_0_] × 100%

where A_0_ is the absorbance of the control (without sample), and A_1_ is that of the sample reaction.

### 2.7. Radical Scavenging Activity of ABTS

The ABTS capacity was evaluated following the previously reported [34]. ABTS^•+^ radicals were produced by mixing 28 mM ABTS with 9.6 mM potassium persulfate and keeping the mixture in the dark for 16 h. The solution was subsequently diluted to achieve an absorbance of 0.70 ± 0.02 at 740 nm. For measurement, the sample solution was combined with the ABTS^•+^ reagent, incubated for 6 min, and the absorbance was determined at 740 nm.

Radical inhibition activity (%) = [(B_0_ − B_1_)/B_0_] × 100%

where B_0_ is the control (without the sample), and B_1_ represents the sample.

### 2.8. Superoxide Assay

The ability to scavenge superoxide radicals was evaluated based on a previously described protocol [35]. The reaction mixture (NBT 300 μM, PMS 120 μM, sample, and NADH 468 μM in 16 mM Tris–HCl, pH 8.0) was incubated for 5 min at 25 °C. Absorbance was measured at 560 nm, and the scavenging activity (%) was calculated as:

free radical neutralizing activity (%) = [(C_0_ − C_1_)/C_0_] × 100

where C_0_ and C_1_ refer to the absorbance measured for the control and sample, respectively.

### 2.9. Ferric Reducing Antioxidant Power (FRAP)

The FRAP assay was conducted following a previously described method [36]. The FRAP working solution was prepared by combining acetate buffer with the other reagent components, FeCl_3_, and TPTZ in a ratio of 40:4:4. In the assay, 200 μL of the sample was mixed with 1800 μL of the FRAP reagent and incubated at 37 °C for 40 min. Absorbance was then recorded at 593 nm. Antioxidant capacity was calculated and expressed as mmol Trolox equivalent (TE) per gram of extract using a Trolox standard curve.

### 2.10. Evaluation of α-Glucosidase Activity

The α-glucosidase inhibitory activity was assessed following the method described by [37]. The enzyme solution (Sigma-Aldrich, St. Louis, MO, USA) was prepared in 0.1 M sodium phosphate buffer (pH 6.8, Showa Chemical Co. Ltd, Chuo-ku, Japan) at a final concentration of 1 U/mL. In each reaction mixture, 100 μL of the sample was combined with 20 μL of the enzyme solution. The reaction started by adding 380 μL of p-nitrophenyl-α-D-glucopyranoside (p-NPG, 0.53 mM, Alfa Aesar, Lancashire, UK) as the substrate. After incubation at 37 °C for 40 min, the reaction was stopped by adding 500 μL of 0.1 M Na_2_CO_3_. (Showa Chemical Co. Ltd, Chuo-ku, Japan) The absorbance was then recorded at 405 nm.

α-glucosidase activity = [(D_0_ − D_1_)/D_0_] × 100%

where D_0_ denotes the absorbance of the control, and D_1_ refers to the absorbance of the sample.

### 2.11. Acetylcholinesterase (AChE) Activity Assay

The modulatory effect on AChE activity was determined based on a previously described procedure with minor modifications [38]. The reaction mixture contained phosphate buffer (pH 8.0, SHOWA Chemical Co. Ltd, Chuo-ku, Japan), the test sample, AChE (Sigma-Aldrich, St. Louis, MO, USA), and DTNB (15 mM, Sigma-Aldrich, St. Louis, MO, USA), which were pre-incubated at 25 °C for 10 min. The reaction was subsequently initiated by the addition of acetylthiocholine iodide (AChI, 15 mM, Sigma-Aldrich, St. Louis, MO, USA) and allowed to proceed for 10 min at 20 °C. Absorbance was subsequently measured at 405 nm. The modulation rate (%) was calculated as follows:

Percent reduction in AChE (%) = [(E_control_ − E_sample_)/E_control_] × 100%

where E_control_ refers to the absorbance of the control, and E_sample_ refers to the absorbance of the sample.

### 2.12. RAW264.7 Cell

RAW264.7 macrophages were provided by Prof. Shu-Ling Fu (National Yang Ming Chiao Tung University, Taiwan). Cells were maintained in Dulbecco’s modified Eagle’s medium (DMEM; HiMedia, Mumbai, Maharashtra, India) containing 10% fetal bovine serum (Product No. 89510-886, Avantor, Radnor, PA, USA) and 1% penicillin–streptomycin (Gibco, Thermo Fisher Scientific, Waltham, MA, USA). Cell cultures were maintained at 37 °C in a humidified incubator with 5% CO_2_. Dulbecco’s phosphate-buffered saline (DPBS, Elabscience Biotechnology Inc., Wuhan, China) was purchased from Biological Industries (Foreston, MN, USA), and Cell banker 1 used for cell storage was obtained from ZENOAQ (Fukushima, Japan).

### 2.13. MTT Assay

Cytotoxicity was evaluated using the MTT assay [39]. A total amount of 4 × 10^6^ cells was placed in 96-well plates and incubated for 24 h. The cells were then treated with different concentrations of the samples in the presence of LPS (100 ng/mL) (Sigma-Aldrich, St. Louis, MO, USA) for 24 h. MTT solution (0.5 mg/mL) (Thermo Fisher Scientific, Waltham, MA, USA) was added and incubated for 3 h. After the reaction finished, the medium was removed, and the crystals were dissolved in DMSO (Toufen City, Miaoli County 351, Taiwan). Absorbance was measured at 570 nm.

### 2.14. LPS-Induced In Vitro Evaluation of Physiological Response

NO production in LPS-induced RAW264.7 macrophages was evaluated using the Griess reaction according to a previously reported method [40] with modifications. RAW264.7 cells were seeded in 96-well plates at 4 × 10^4^ cells per well, corresponding to a total of approximately 4 × 10^6^ cells per plate, and incubated for 24 h. Cells were incubated with the test compounds in the presence of LPS (100 ng/mL) (Sigma-Aldrich, St. Louis, MO, USA) for 24 h. The culture medium was subsequently harvested and analyzed for nitrite using the Griess reaction. ELISA reader was measured at 550 nm, and nitric oxide production was quantified using a sodium nitrite (NaNO_2, _Sigma-Aldrich, St. Louis, MO, USA) standard curve and expressed as μM nitrite equivalents.

### 2.15. Western Blot Analysis

Protein expression was analyzed by Western blotting using a previously described protocol [41]. RAW264.7 cells (1 × 10^6^ cells) were seeded in 6 cm culture dishes for 24 h to allow for cell attachment. The cells were then exposed to the tested compounds in the presence of lipopolysaccharide (100 ng/mL) (Sigma-Aldrich, St. Louis, MO, USA) for an additional 24 h. Following treatment, the cells were washed with DPBS and lysed to obtain total cellular proteins. The lysates were then centrifuged at 4 °C for 30 min., and the clear supernatants were collected as protein samples. For electrophoresis, protein extracts were combined with 4× Dye and heated at 100 °C for 10 min. The protein samples were resolved by SDS–polyacrylamide gel electrophoresis using 10% and 8% resolving gels with 5% stacking gels. After electrophoresis, proteins were transferred onto polyvinylidene difluoride (PVDF, Merck Millipore, Burlington, MA, USA) membranes. The membranes were blocked overnight with 5% bovine serum albumin. After blocking, the membranes were washed three times with TBST and incubated with primary antibodies (1:1000) for 24 h at 4 °C. On the following day, the membranes were washed three times with TBST and subsequently incubated with horseradish peroxidase (HRP, Sigma, St. Louis, MO, USA)-linked secondary antibodies (1:3000) for 1 h at 25 °C. The protein signals were detected with an enhanced chemiluminescence (ECL) detection system. When necessary, the membranes were stripped and reprobed to detect total protein levels. Band intensities were analyzed using ImageJ software (version 1.54p) [42]. Detailed information on the primary antibodies is provided in Supplementary Table S1.

### 2.16. Molecular Docking

Computational docking was conducted to explore the possible interactions between the isolated compounds and several target enzymes, following previously established computational procedures [43]. The 3D crystal structures of α-glucosidase (PDB ID: 3A4A) [44], acetylcholinesterase (PDB ID: 1ACJ) [45], COX-2 (PDB ID: 3NT1) [46], and iNOS (PDB ID: 1M9T) [47] were downloaded from the Protein Data Bank (PDB). Before performing the docking calculations, the protein structures were processed by eliminating crystallographic water molecules using BIOVIA Discovery Studio 2021 (version 21, San Diego, CA, USA). Ligand molecules were retrieved from the PubChem database and converted into 3D structures using Chem3D software (version 16.0). Subsequently, hydrogen atoms and Gasteiger charges were added to both the protein and ligand models with AutoDock Tools (version 1.5.6), and the prepared structures were exported in PDBQT format for docking analysis. Docking simulations were then performed using AutoDock Vina (version 1.1.2). Docked conformations with the best binding scores were chosen for subsequent visualization and interaction analysis in BIOVIA Discovery Studio 2021 (version 21, San Diego, CA, USA).

### 2.17. Prediction of Physicochemical Properties Using In Silico Methods

The physicochemical characteristics of the investigated bioactive ingredients, including molecular weight, lipophilicity (logP), polar surface area (PSA), molecular volume, and compliance with Lipinski’s parameters, were evaluated using the Molecular Property Prediction Tool (MolSoft LLC, San Diego, CA, USA; available at https://molsoft.com/mprop/, accessed on 22 March 2026) [48]. This platform estimates molecular descriptors based on input SMILES or structural data through machine learning models and empirically derived algorithms developed from large datasets of food- and plant-derived ingredients. All structures were manually validated and converted to canonical SMILES before analysis to ensure reproducibility and computational accuracy. These parameters provide a comprehensive overview of molecular behavior in aqueous environments and help predict their potential bioavailability and functional compatibility in food systems.

### 2.18. Statistical Analysis

All measurements were carried out in three independent experiments, and the results are presented as mean ± standard deviation (SD). Differences among groups were evaluated by one-way analysis of variance (ANOVA) followed by Tukey’s multiple comparison test. Statistical analyses were conducted using IBM SPSS Statistics software (version 29.0; IBM Corp., Armonk, NY, USA) [49]. For NO production and MTT-based cell viability assays, treatment groups were compared with the LPS control using Student’s *t*-test. Levels of statistical significance were set at *p* < 0.05, *p* < 0.01, and *p* < 0.001.

## 3. Results and Discussion

### 3.1. TPC, TFC, and Extraction Yields of Taraxacum mongolicum from Different Solvents

Comparative extractions of *Taraxacum mongolicum* using solvents of different polarity revealed marked differences in extraction yield, suggesting that solvent polarity strongly affects the recovery efficiency of phytochemicals. As shown in Table 1, a clear trend was observed between solvent polarity and extraction yield, indicating that solvent polarity plays an important role in phytochemical recovery. Among all extraction conditions, water at 100 °C produced the highest overall yield (30.25%), followed by methanol extraction (12.45%), whereas *n*-hexane and dichloromethane exhibited relatively low extraction efficiencies.

Regarding phenolic recovery, the methanol extract showed the highest TPC (42.59 ± 3.10 mg GAE/g), while the water (100 °C) extract also displayed relatively high TPC values (32.35 ± 5.74 mg GAE/g). These results are consistent with previous studies showing that *Taraxacum* species are rich in caffeic acid derivatives, which are highly polar compounds that can be efficiently extracted using polar solvents [50]. The relatively high TPC observed in the water (100 °C) extract suggests that thermal treatment may facilitate phenolic release. The elevated extraction temperatures can disrupt plant cell wall structures and weaken interactions between phenolic compounds and cell wall polysaccharides or proteins, thereby promoting the release of bound or conjugated phenolics [51].

In contrast, extracts obtained using *n*-hexane and dichloromethane contained negligible amounts of total phenolics (< 1 mg GAE/g), reflecting the limited ability of non-polar solvents to solubilize hydroxylated phenolic acids. However, the dichloromethane extract exhibited a moderate total flavonoid content (13.03 ± 4.72 mg QE/g), comparable to that of the EtOAc and acetone extracts. This result suggests that low-to-medium-polarity solvents may preferentially enrich relatively hydrophobic flavonoid subclasses, such as aglycones or partially methoxylated flavonoids [52].

Overall, the methanol extract was characterized by concurrently high TPC and TFC, indicating broad recovery of phenolic acids and flavonoid compounds. In contrast, the water (100 °C) extract showed the highest extraction yield together with substantial phenolic content, suggesting enrichment of highly polar phenolic conjugates and other hydrophilic components.

### 3.2. Analysis of Antioxidant Activities in Solvent Extracts

To comprehensively evaluate the antioxidant potential of *Taraxacum mongolicum*, a traditional edible herb increasingly recognized as a food-grade bioresource, four complementary in vitro assays, including assessment of antioxidant activity, were examined using DPPH, ABTS, superoxide radical scavenging, and FRAP assays, representing different mechanisms like hydrogen atom transfer, electron transfer, and metal ion reduction [53]. The detailed quantitative results are summarized in Table 2.

The methanol extract showed the highest radical scavenging activity in both DPPH and ABTS assays, with SC_50_ values of 75.40 ± 2.30 and 74.75 ± 5.52 μg/mL, respectively. This was followed by the water (100 °C) extract (114.37 and 117.32 μg/mL) and the water extract (242.57 and 251.29 μg/mL). In contrast, extracts obtained using low-polarity solvents, including ethyl acetate, acetone, dichloromethane, and n-hexane, did not show appreciable radical scavenging activity in these assays (SC_50_ > 400 μg/mL). Overall, these results indicate a relationship between solvent polarity and radical scavenging capacity, which may reflect differences in the polarity distribution of antioxidant constituents among the extracts.

A distinct activity pattern was observed in the superoxide radical scavenging assay. The water (100 °C) extract exhibited the strongest scavenging activity (SC_50_ = 84.04 ± 2.22 μg/mL), followed by the water extract (193.77 ± 4.23 μg/mL), whereas the methanol and ethanol extracts showed no significant activity (SC_50_ > 400 μg/mL). Superoxide radicals predominantly exist in aqueous reaction systems, and their scavenging behavior may be associated with the hydrophilic characteristics of antioxidant components [54,55]. In addition, high-temperature water extraction may enhance the recovery of polar phenolic materials, which could further influence the performance of the extracts during the superoxide radical scavenging test [51].

In the FRAP assay, the methanol extract exhibited the highest reducing power (625.67 ± 9.19 mM TE/g), followed by the water (100 °C) extract (229.27 ± 4.41 mM TE/g) and the water extract (119.63 ± 4.12 mM TE/g). In contrast, extracts obtained using low-polarity solvents showed minimal reducing capacity (<25 mM TE/g). As FRAP is operating via a single-electron transfer (SET) mechanism, an antioxidant assay that primarily reflects the reducing capacity of a sample, several studies have indicated a positive relationship between FRAP values and TPC [53]. The differences in FRAP activity observed among the extracts are consistent with variations in their phenolic content.

Collectively, these results demonstrate that solvent polarity has a significant influence on the antioxidant performance of *Taraxacum mongolicum* extracts across different in vitro assays. Such differences may be attributed to variations in the polarity distribution of extracted constituents as well as the reaction characteristics inherent to each antioxidant assay.

### 3.3. Analysis of Enzyme Inhibitory Activities in Solvent Extract

To further explore the enzyme-modulating properties of *T. mongolicum*, enzyme inhibition assays were employed as an initial mechanistic screening approach. The modulatory effects of extracts obtained using solvents of different polarity were evaluated against α-glucosidase and acetylcholinesterase (AChE) in Table 3.

In the α-glucosidase inhibition assay, the *n*-hexane extract exhibited the strongest inhibitory activity (IC_50_ = 176.73 ± 13.13 μg/mL), showing greater potency than the positive control acarbose (IC_50_ = 460.71 ± 27.80 μg/mL). In addition, the dichloromethane, ethyl acetate, acetone, and ethanol extracts displayed moderate inhibitory effects, whereas the water and water (100 °C) extracts did not exhibit activity (IC_50_ > 800 μg/mL). Overall, α-glucosidase inhibitory activity was mainly distributed in low- to medium-polarity extracts, suggesting that modulation of this enzyme may be associated with chemical constituents that are not highly hydrophilic. Previous studies have indicated that α-glucosidase inhibitors are not restricted to phenolic compounds, and that lipid-related or other non-phenolic constituents may also contribute to inhibitory effects [56,57]. This provides a reasonable basis for the pronounced activity observed in the low-polarity extracts in the present study.

In the acetylcholinesterase inhibition assay, several extracts exhibited moderate inhibitory activity. The ethyl acetate, dichloromethane, *n*-hexane, and water extracts showed comparable IC_50_ values, ranging approximately from 68 to 97 μg/mL, whereas the methanol and ethanol extracts displayed relatively weaker inhibitory effects. These results indicate that AChE inhibitory activity was not confined to a single polarity fraction but may instead reflect the combined contribution of constituents with different polarity characteristics. Consistent with this interpretation, plant-derived AChE inhibition has frequently been attributed to additive or synergistic effects among multiple classes of compounds rather than to a single dominant constituent [58,59].

Overall, *Taraxacum mongolicum* extracts obtained using solvents of different polarity exhibited distinct and enzyme-specific inhibitory activity profiles across the three enzyme assays evaluated. These findings highlight the critical role of extraction solvent polarity in shaping enzyme-modulating properties and reflect the selective interactions between the chemically diverse constituents of *T. mongolicum* and different enzyme targets.

### 3.4. Analysis of NO Inhibitory Activities in Solvent Extracts

**Figure 3:**
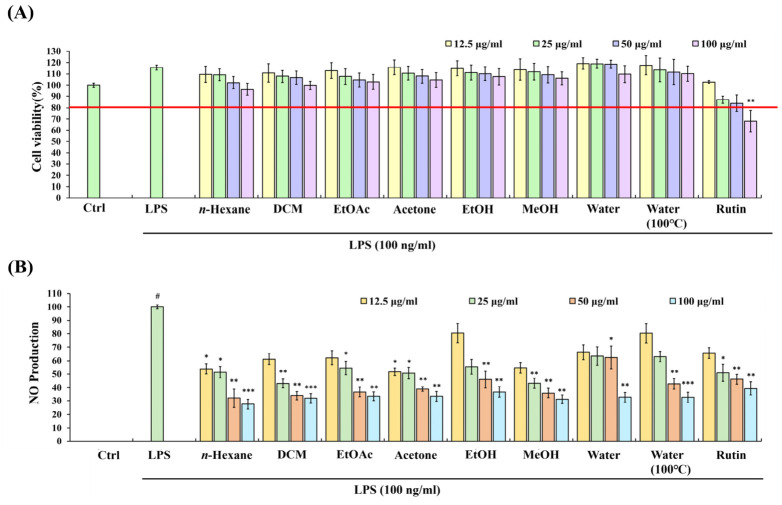
Effects of *Taraxacum mongolicum* solvent extracts on cell viability and NO production following LPS stimulation. (**A**) Cell viability after treatment with extracts (12.5–100 μg/mL). (**B**) Inhibition of NO production. Data are expressed as mean ± SD (n = 3). Rutin served as the positive control. Statistical analysis was performed using Student’s *t*-test. # indicates a significant difference compared with the untreated control group. The red line indicates the 80% cell viability threshold used for cytotoxicity evaluation. * *p* < 0.05, ** *p* < 0.01, and *** *p* < 0.001 indicate significant differences compared with the LPS-treated group.

As shown in Figure 3A, none of the *Taraxacum mongolicum* solvent extracts caused significant cytotoxic effects on RAW264.7 within the concentration range (12.5, 25, 50, and 100 μg/mL), with cell viability consistently remaining above 80%. These results indicate that the selected concentrations were appropriate for subsequent NO inhibition analysis and that the observed reduction in NO production was not attributable to nonspecific cell damage.

In Figure 3B, LPS stimulation markedly increased NO production in RAW264.7 cells. Treatment with *T. mongolicum* extracts obtained using different solvents resulted in concentration-dependent suppression of NO production. Overall, most organic solvent extracts showed pronounced inhibitory effects on NO production. At 100 μg/mL, the methanol, ethyl acetate, *n*-hexane, and dichloromethane extracts reduced NO levels to approximately 31.15%, 33.42%, 27.58%, and 31.91%, respectively, indicating substantial NO-modulating activity.

Table 4 revealed that the methanol extract exhibited the lowest IC_50_ value (15.61 ± 1.60 μg/mL), suggesting the strongest NO inhibition, followed by the ethyl acetate (16.17 ± 0.91 μg/mL), *n*-hexane (19.14 ± 0.63 μg/mL), and dichloromethane extracts (19.48 ± 2.11 μg/mL). In contrast, the aqueous extract showed a significantly higher IC_50_ value (71.50 ± 12.31 μg/mL), indicating limited efficacy in suppressing LPS-induced NO production. Notably, the methanol, ethyl acetate, *n*-hexane, and dichloromethane extracts exhibited NO inhibitory effects comparable to the positive control rutin at equivalent concentrations, particularly under high-dose conditions.

Overall, *T. mongolicum* extracts obtained using solvents of different polarity displayed distinct NO inhibitory profiles, suggesting that NO-modulating bioactive constituents are unevenly distributed among the solvent extractions. This pattern partially corresponds with the previously observed TPC and TFC results, as the methanol and ethyl acetate extracts, which exhibited higher TPC and TFC values, also demonstrated stronger NO inhibitory activity. These observations support the notion that phenolic acids, polyphenols, and flavonoids may contribute to the regulation of NO production in macrophages. However, the notable NO inhibitory effects observed for certain low-polarity extracts [60], such as the *n*-hexane and dichloromethane, despite their relatively lower TPC and TFC levels, suggest that NO modulation is not exclusively mediated by highly polar phenolic compounds. Instead, other less hydrophilic secondary metabolites [61], including lipid-derived or non-phenolic constituents, may also participate in the regulation of NO production in macrophages [62].

### 3.5. Antioxidant Activities of Taraxacum mongolicum in Isolated Components

Evaluation of the isolated constituent provided further insights into the molecular determinants of antioxidant activity. Among all tested constituents (Table 5), chicoric acid exhibited the most potent antioxidant effects, with SC_50_ values of 13.01 ± 0.54 μM, 15.75 ± 4.79 μM, and 21.97 ± 2.65 μM in the DPPH, ABTS, and superoxide assays, respectively, and an exceptionally high FRAP value of 18,119.77 ± 145.30 mM TE/g. Chicoric acid, a caffeoyltartaric acid derivative containing two catechol moieties, possesses a π-conjugated system that facilitates both electron transfer and hydrogen atom donation, displaying dual HAT–SET antioxidant mechanisms [63]. Its activity surpassed that of the positive control BHT, indicating that chicoric acid serves as a key functional indicator of the antioxidant capacity in *Taraxacum mongolicum* [64].

Luteolin and caftaric acid also showed moderate radical scavenging capacities. The high activity of luteolin is likely attributed to its 3′,4′-dihydroxy B-ring and C2 and C3 double bond, which effectively stabilize phenoxyl radicals and chelate metal ions, thus conferring both HAT and SET capabilities [64]. Although caftaric acid has a simpler structure, its caffeoyl group enables resonance stabilization of radicals and participates in Proton-Coupled Electron Transfer reactions, maintaining strong antioxidant performance even in aqueous environments. These two bioactive ingredients illustrate complementary chemical frameworks that contribute to both radical scavenging and metal ion reduction [65].

In contrast, apigenin, vanillic acid, and syringic acid exhibited relatively weak scavenging activity in DPPH and ABTS assays (SC_50_ > 100 μM), though they likely function as electron donors in FRAP assays. The limited hydroxyl substitution and conjugation in their structures reduce their radical stabilization ability. Nonetheless, the diverse polyphenolic profile of *Taraxacum mongolicum* collectively forms a synergistic antioxidant network. Overall, the antioxidant potency of *Taraxacum mongolicum* primarily derives from its structurally diverse phenolic constituents, with chicoric acid acting as the dominant dual mechanism antioxidant, while luteolin and caftaric acid provide complementary radical scavenging and metal reducing contributions [65].

**Table 5:** Antioxidant activities of *T. mongolicum* isolated components.

Compounds	SC_50_ (μM) ^A^			TE (mM/g) ^B^
	DPPH	ABTS	Superoxide	FRAP
Chicoric acid (**1**)	13.01 ± 0.54 ^a^	15.75 ± 4.79 ^a^	21.97 ± 2.65 ^a^	18119.77 ± 145.30 ^e^
Luteolin (**2**)	26.63 ± 5.38 ^b^	28.05 ± 1.22 ^b^	> 100	15520.61 ± 34.17 ^c^
Caftaric acid (**3**)	47.40 ± 0.79 ^c^	37.42 ± 6.59 ^c^	84.84 ± 5.82 ^b^	6678.04 ± 37.67 ^b^
Apigenin (**4**)	> 100	> 100	> 100	847.33 ± 16.60 ^a^
Vanillic acid (**5**)	> 100	28.92 ± 1.37 ^b^	> 100	6598.99 ± 90.86 ^b^
Syringic acid (**6**)	42.37 ± 2.88 ^c^	26.70 ± 1.91 ^ab^	> 100	16370.13 ± 39.89 ^d^
BHT ^C^	53.72 ± 3.74 ^d^	40.96 ± 4.87 ^c^	─	6557.42 ± 8.30 ^b^
Cynaroside ^D^	─	─	23.01 ± 2.62 ^a^	─

^A^ SC_50_ refers to the sample concentration required to scavenge 50% of radicals. ^B^ The reducing capacity in the FRAP assay is reported as mM TE per g extract. ^C^ BHT served as a positive control. ^D^ Cynaroside served as the standard compound for evaluating superoxide scavenging activity. Experimental results are presented as mean ± SD (n = 3). Different letters (a–e) indicate statistically significant differences among samples at *p* < 0.05 according to Tukey’s test.

### 3.6. Enzyme Inhibitory Activities of Taraxacum mongolicum in Isolated Components

Among the isolated constituents (Table 6), apigenin demonstrated the highest α-glucosidase modulating activity (IC_50_ = 53.28 ± 3.72 μM), which was significantly greater than the inhibitory activity of the positive control, acarbose (IC_50_ = 630.73 ± 5.23 μM), followed by chicoric acid and luteolin. These findings suggest that the phenolic and flavonoid constituents of *Taraxacum mongolicum* play a crucial role in regulating carbohydrate-metabolizing enzymes. Similar α-glucosidase reduction has also been observed in other plant-based foods, and these bioactive constituents may help attenuate postprandial glucose elevation by slowing starch digestion and glucose release [66].

In the acetylcholinesterase (AChE) decrease assay, vanillic acid (IC_50_ = 100.07 ± 11.68 μM) and syringic acid (IC_50_ = 128.22 ± 16.57 μM) exhibited the highest reduction among the tested phenolic acids, which was consistent with the moderate reduction observed in the polar extracts. These findings indicate that small phenolic acids present in *T. mongolicum* may contribute to the regulation of AChE activity, thereby supporting the maintenance of neuronal homeostasis. Such effects can be regarded as a form of dietary support for regulating neurotransmission, suggesting a potential nutritional relevance to cognitive wellness associated with the consumption of *Taraxacum mongolicum* [67].

**Table 6:** Enzyme suppression activities of *Taraxacum mongolicum* isolated components.

Compounds	IC_50_ (μM) ^A^	
	α-Glucosidase	AChE
Chicoric acid (**1**)	94.23 ± 11.92 ^b^	295.51 ± 12.65 ^d^
Luteolin (**2**)	121.91 ± 15.60 ^b^	> 400
Caftaric acid (**3**)	> 800	231.55 ± 13.72 ^bc^
Apigenin (**4**)	53.28 ± 3.72 ^a^	247.91 ± 20.74 ^cd^
Vanillic acid (**5**)	> 800	100.07 ± 11.68 ^a^
Syring acid (**6**)	> 800	128.22 ± 16.57 ^a^
Acarbose ^B^	630.73 ± 5.23 ^c^	─
Chlorogenic acid ^C^	─	186.22 ± 6.66 ^b^

^A^ IC_50_ corresponds to the concentration that inhibits 50% of enzyme activity. ^B^ Acarbose served as the reference inhibitor in the α-glucosidase assay. ^C^ Chlorogenic acid functioned as the reference compound in the AChE. Results are reported as mean ± SD from three independent measurements (n = 3). Different superscript letters (a–d) indicate significant differences between groups (*p* < 0.05, Tukey’s test).

### 3.7. NO Production Inhibitory Activities of Taraxacum mongolicum in Isolated Components

**Figure 4:**
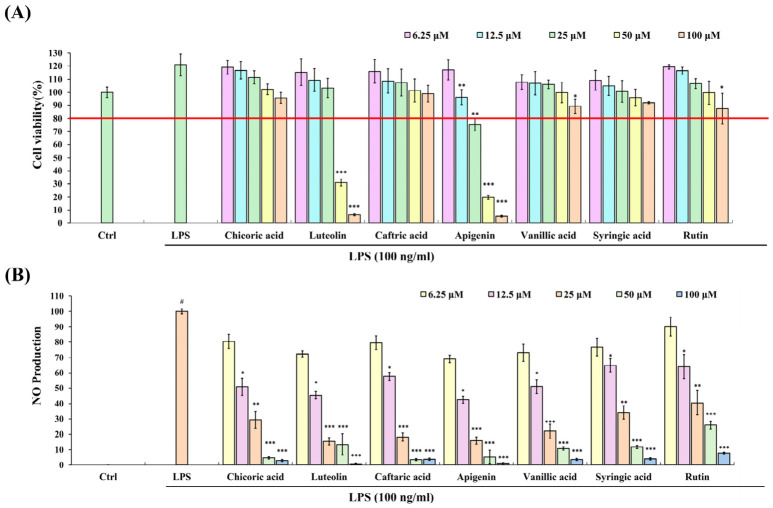
NO inhibitory activities of *Taraxacum mongolicum* compounds in RAW264.7 cells. (**A**) Cell viability of RAW264.7 cells treated with isolated compounds of *T. mongolicum* at concentrations of 6.25, 12.5, 25, 50, and 100 μM. (**B**) Inhibitory effects of *T. mongolicum* solvent extracts on NO production in RAW264.7. Results are shown as mean ± SD (n = 3). Rutin served as the reference control. The red line indicates the 80% cell viability threshold used for cytotoxicity evaluation. Statistical differences were evaluated by Student’s *t*-test. # indicating *p* < 0.05 versus the untreated control, while *, **, and *** denote *p* < 0.05, 0.01, and 0.001 compared with the LPS-treated group.

As shown in Figure 4A, among the isolated compounds, chicoric acid, luteolin, caftaric acid, vanillic acid, and syringic acid maintained cell viability above 80% in RAW264.7 at concentrations ≤ 25 μM, indicating that this concentration range was suitable for NO inhibition analysis without causing significant cytotoxic interference. In contrast, luteolin and apigenin showed a decreasing trend in cell viability at concentrations ≥ 50 μM, suggesting that these compounds may have cytotoxic effects at higher doses.

As illustrated in Figure 4B, LPS stimulation markedly increased NO production in RAW264.7. Treatment with individual isolated compounds resulted in a concentration-dependent reduction in NO production, although the extent of inhibition varied among compounds. At 25 μM, chicoric acid, luteolin, caftaric acid, apigenin, vanillic acid, and syringic acid reduced NO production by approximately 70.55%, 84.45%, 81.75%, 83.39%, 77.99%, and 65.75%, respectively. These inhibitory effects were greater than those observed for the positive control rutin at the same concentration (59.53%), indicating that most of the isolated constituents exhibited substantial NO-modulating activity.

The IC_50_ values for NO inhibition are summarized in Table 7. Luteolin (9.04 ± 0.14 μM) and apigenin (9.81 ± 0.82 μM) displayed the strongest inhibitory effects, followed by chicoric acid (14.21 ± 0.75 μM) and syringic acid (12.76 ± 0.12 μM), whereas vanillic acid and caftaric acid exhibited relatively higher IC_50_ values, indicating weaker NO inhibitory potency. These results suggest that flavonoids may represent key contributors to NO regulation in the methanol extraction of *Taraxacum mongolicum*.

From a structural perspective, luteolin and apigenin possess conjugated double-bond systems and multiple phenolic hydroxyl groups, which may influence intracellular redox balance and thereby indirectly modulate inflammatory signaling pathways associated with NO production [68]. In addition, caffeoyl derivatives such as chicoric acid and caftaric acid contain catechol moieties that may participate in regulating intracellular oxidative stress or interfere with the activation of enzymes involved in NO generation [69]. Previous studies have suggested that suppression of NO production is not solely dependent on direct radical scavenging activity but may also involve indirect modulation of cellular signaling and enzyme activity.

### 3.8. Analysis of Inflammatory-Related Protein of Taraxacum mongolicum in Solvent Extracts

#### 3.8.1. Modulation of iNOS and COX-2 Expressions by the Methanol Extract of Luteolin and Apigenin

**Figure 5:**
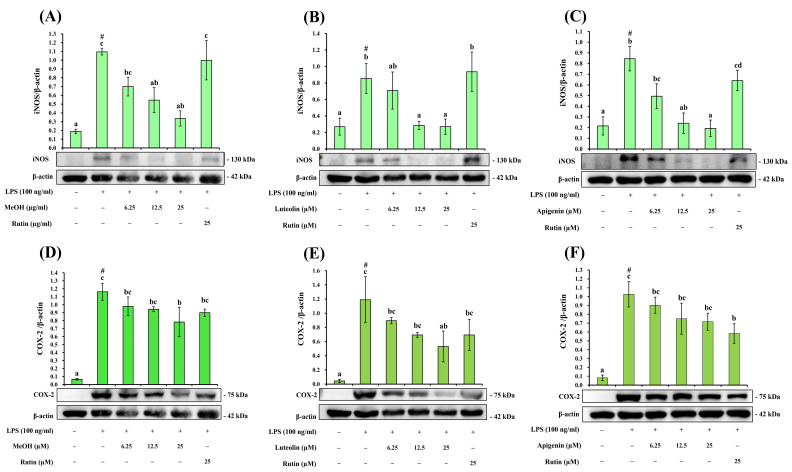
Effects of (**A**) MeOH extract, (**B**) luteolin, and (**C**) apigenin on iNOS expression. Effects of (**D**) MeOH extract, (**E**) luteolin, and (**F**) apigenin on COX-2 expression. Different letters (a–d) indicate significant differences among groups (*p* < 0.05, Tukey’s test). Rutin served as a positive control. # indicating *p* < 0.05 compared with the LPS-treated group.

To determine whether the observed reduction in NO production was associated with the regulation of inflammation-related enzymes, the protein expression levels of iNOS and COX-2 were further examined by Western blot analysis (Figure 5A–F). LPS stimulation markedly increased the expression of both iNOS and COX-2 compared with the untreated group, indicating successful induction of an inflammatory response in RAW264.7 cells.

Treatment with the methanol extract of *T. mongolicum*, as well as luteolin and apigenin, markedly attenuated the expression of these inflammation-related enzymes. At 25 μg/mL, the methanol extract reduced iNOS and COX-2 protein levels by 69.30% and 32.52%, respectively, relative to the LPS-treated control. Notably, this inhibitory effect was greater than that observed for rutin, suggesting that the methanol extract has a pronounced suppressive effect on iNOS and COX-2 expression.

In the comparison of luteolin and apigenin, both compounds effectively attenuated LPS-induced iNOS expression. At the higher concentration tested, luteolin and apigenin reduced iNOS protein levels by 67.44% and 77.16%, respectively, indicating that these flavonoids may contribute to the regulation of enzymes involved in NO production. Both compounds also suppressed COX-2 expression, with inhibition rates of 55.45% for luteolin and 30.15% for apigenin. However, compared with their effects on iNOS, the inhibitory effects on COX-2 were relatively weaker, suggesting that these flavonoids may exert differential regulatory effects on distinct inflammation-related proteins.

#### 3.8.2. Regulatory Effects on of TNF-α and IL-6 Expressions by the Luteolin and Apigenin

To assess their influence on inflammatory mediators and NO production, luteolin and apigenin were selected as representative compounds, and their effects on cytokine protein levels were analyzed in LPS-treated RAW264.7 cells, with rutin included as a positive control.

**Figure 6:**
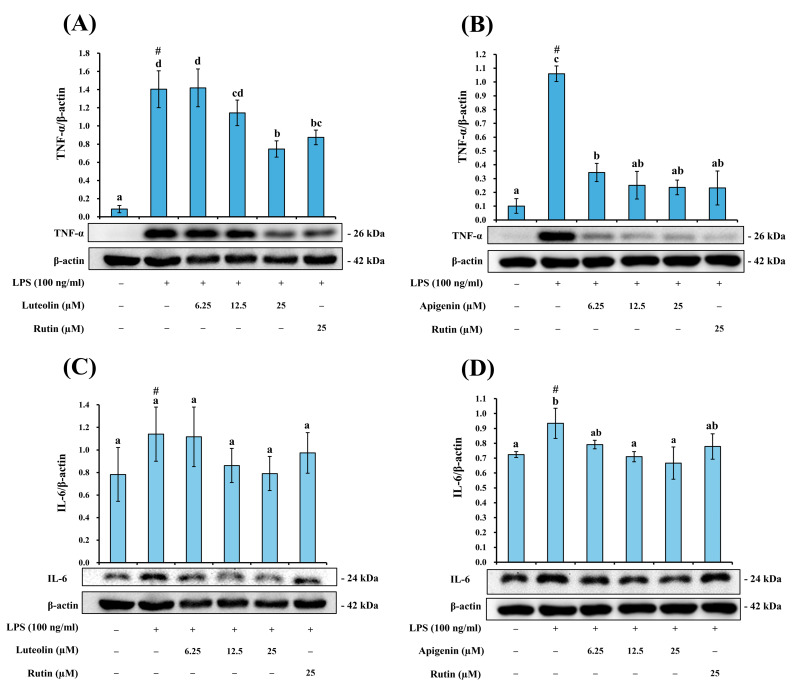
Modulation of LPS-induced TNF-α and IL-6 expression in RAW264.7 cells by the luteolin and apigenin from *T. mongolicum*. Effects of (**A**) luteolin and (**B**) apigenin on TNF-α expression. Effects of (**C**) luteolin and (**D**) apigenin on IL-6 expression. Different letters (a–d) indicate significant differences among groups (*p* < 0.05, Tukey’s test). Rutin served as a positive control. # indicated *p* < 0.05 compared with the LPS-treated group.

As shown in Figure 6A,C, luteolin at 25 μM reduced TNF-α and IL-6 protein expression by 46.81% and 30.63%, respectively, relative to the LPS-treated group. In both cases, the inhibitory effects of luteolin were greater than those observed for rutin at the same concentration. These results suggest that luteolin exerts inhibitory effects on both cytokines, with a relatively stronger effect on TNF-α expression.

In comparison, apigenin displayed markedly stronger inhibitory effects on both pro-inflammatory cytokines. As shown in Figure 6B and Figure 6D, apigenin at 25 μM reduced TNF-α and IL-6 expression by 77.73% and 28.66%, respectively, relative to the LPS-treated group. The inhibitory effect on TNF-α was comparable to that of rutin, whereas the reduction in IL-6 expression was greater than that observed for rutin. These findings suggest that apigenin exerts clear regulatory effects on both TNF-α and IL-6 under the present experimental conditions.

Overall, both luteolin and apigenin effectively attenuated LPS-induced TNF-α and IL-6 expressions. However, differences in their regulatory profiles were observed. Luteolin showed a relatively stronger inhibitory effect on TNF-α expression than on IL-6, whereas apigenin exhibited inhibitory effects on both TNF-α and IL-6 under the present experimental conditions. These findings suggest that the two flavonoids may differ in their regulatory preferences toward specific pro-inflammatory cytokines.

#### 3.8.3. Modulation of MAPK Phosphorylation and IκBα Phosphorylation in the NF-κB Pathway Expression by the Luteolin

**Figure 7:**
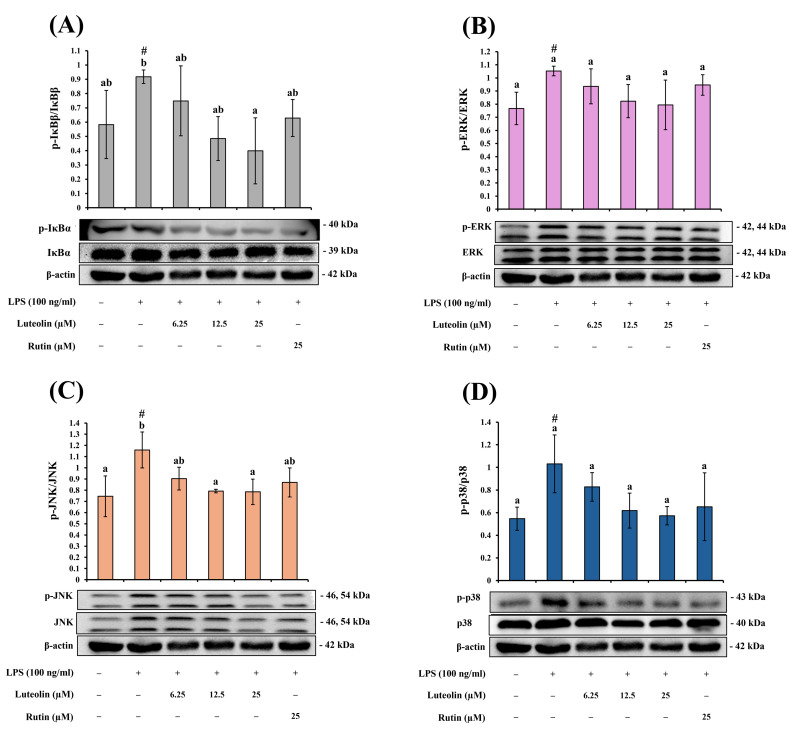
Regulation of NF-κB and MAPK signaling in RAW264.7 cells by luteolin from *T. mongolicum*. Protein expression of (**A**) IκBα, (**B**) ERK, (**C**) JNK, and (**D**) p38 following luteolin treatment. Data are shown as mean ± SD (n = 3). Different letters (a, b) indicate significant differences among groups (*p* < 0.05, Tukey’s test). Rutin served as a positive control. # indicating *p* < 0.05 compared with the LPS-treated group.

In order to examine the upstream signaling mechanisms underlying the anti-inflammatory activity of luteolin, the activation of key proteins involved in the NF-κB and MAPK pathways was analyzed in LPS-treated RAW264.7. As illustrated in Figure 7, LPS markedly enhanced the phosphorylation levels of IκBα, ERK, JNK, and p38 compared with the untreated control cells. However, luteolin treatment markedly reduced these phosphorylation events in a dose-dependent manner, with the greatest inhibitory effect observed at the highest concentration.

At 25 μM concentration, luteolin reduced IκBα phosphorylation by 56.59% relative to the LPS group, which was substantially greater than the inhibition observed for the positive control rutin. This result suggests that luteolin may interfere with NF-κB activation at an early regulatory step, consistent with its ability to suppress downstream inflammatory mediators. In addition, luteolin also attenuated the MAPK signaling pathway. Phosphorylation of JNK was reduced by 34.24% at 25 μM, which was comparable to the inhibitory effect of rutin (31.53%), while ERK phosphorylation was decreased by 25.48%, greater than that of rutin (10.10%). Similarly, p38 phosphorylation was reduced by 44.51%, which was also greater than the inhibition by rutin (36.77%). The data suggest that luteolin mitigates LPS-induced inflammatory signaling by affecting both NF-κB and MAPK pathways. The stronger suppression of IκBα phosphorylation compared with MAPK proteins indicates that blockade of NF-κB signaling may play a dominant role in reducing downstream inflammatory responses.

#### 3.8.4. Modulation of MAPK Phosphorylation and IκBα Phosphorylation in the NF-κB Pathway Expression by Apigenin

**Figure 8:**
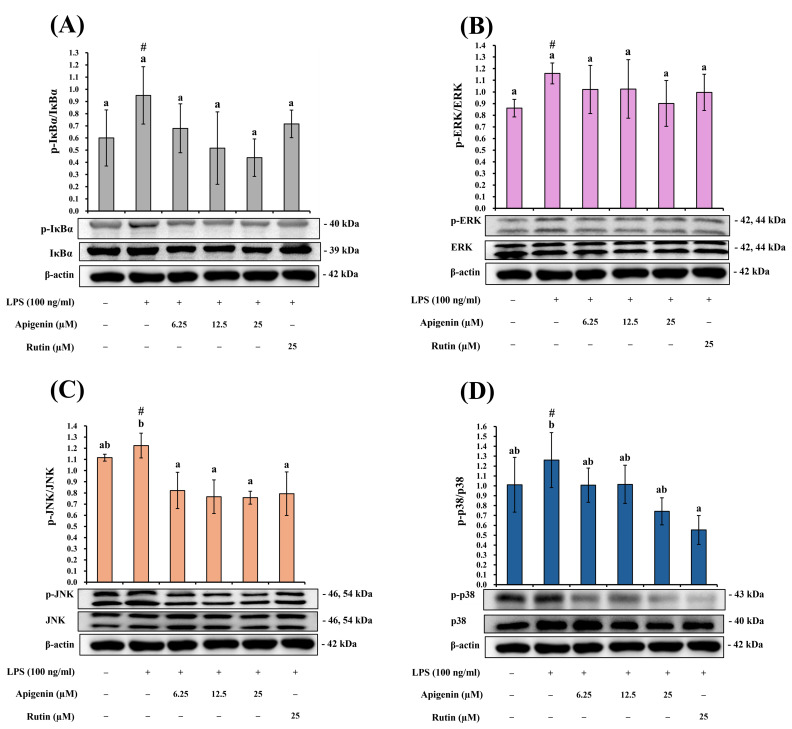
Modulation of LPS-induced NF-κB and MAPK signaling pathways in RAW264.7 macrophages by Apigenin from *T. mongolicum*. (**A**) Effect of apigenin on IκBα expression. (**B**) Effect of apigenin on ERK expression. (**C**) Effect of apigenin on JNK expression. (**D**) Effect of apigenin on p38 expression. Data are presented as mean ± SD (n = 3). Different letters (a, b) indicate statistically significant differences among groups (*p* < 0.05, Tukey’s test). Rutin was used as a positive control. # indicating *p* < 0.05 compared with the LPS-treated group.

To compare the differential regulatory effects of flavonoids on inflammatory signaling pathways, the influence of apigenin on the phosphorylation of key proteins within the NF-κB and MAPK pathways was investigated. As shown in Figure 8, treatment with apigenin 25 μM significantly decreased the phosphorylation of multiple signaling proteins in LPS-treated RAW264.7 cells. In particular, apigenin shows the lower IκBα phosphorylation by 53.85% relative to the LPS-stimulated group. This inhibitory effect exceeded that of rutin, which produced a reduction of 24.66%, suggesting that apigenin can effectively suppress activation of the NF-κB pathway.

Furthermore, in the MAPK signaling pathway, apigenin at 25 μM reduced the phosphorylation levels of JNK, ERK, and p38 by 38.15%, 14.07%, and 41.88%, respectively. Among these proteins, the inhibitory effects on JNK and p38 phosphorylation were greater than those observed for rutin under the same experimental conditions, indicating that apigenin also exerts regulatory effects on MAPK-related signaling proteins. Overall, apigenin exerted inhibitory effects on both NF-κB and MAPK signaling pathways.

#### 3.8.5. Modulation of M2 Phenotype Markers: Arg-1 and KLF4

**Figure 9:**
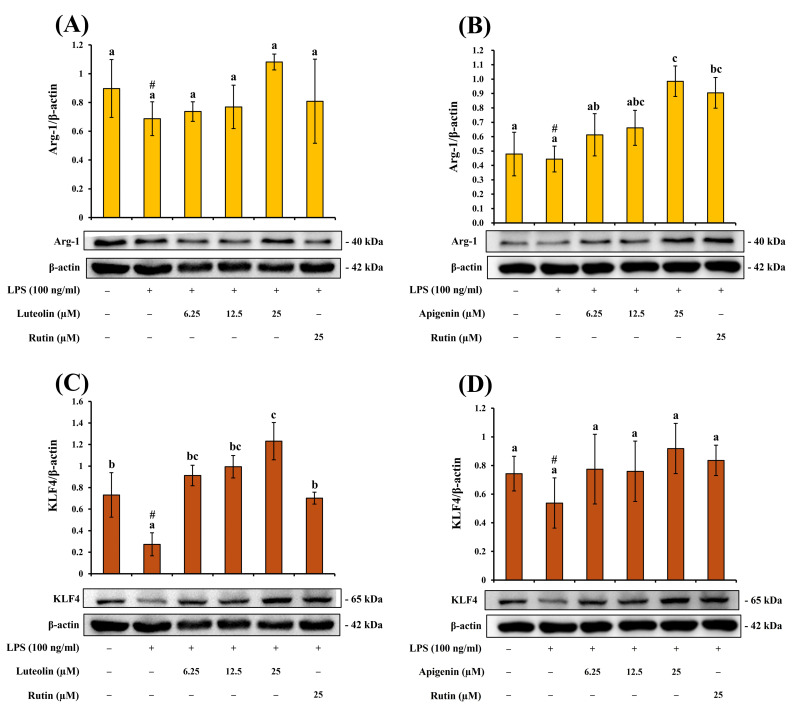
Influence of luteolin and apigenin isolated from *T. mongolicum* on M2 macrophage polarization markers in RAW264.7 cells. Effects of (**A**) luteolin and (**B**) apigenin on Arginase-1 expression. Effects of (**C**) luteolin and (**D**) apigenin on KLF4 expression. Values represent mean ± SD (n = 3). Different letters (a–c) indicate significant differences between groups (*p* < 0.05, Tukey’s test). Rutin was used as the reference compound. # indicating a significant difference compared with the LPS-treated group (*p* < 0.05).

In Figure 9, luteolin and apigenin effectively suppressed LPS-induced inflammation-related protein expression, their potential involvement in macrophage phenotype regulation was further evaluated. Accordingly, the expression of the M2-associated immunoregulatory markers Arg-1 and KLF4 was analyzed to assess whether modulation of inflammatory responses was accompanied by changes in macrophage activation status.

As shown by the results, in LPS-stimulated RAW264.7 macrophages, luteolin at 25 μM increased Arg-1 expression by 1.57-fold and KLF4 expression by 4.50-fold relative to the LPS-treated group. These increases were greater than those observed in the positive control, rutin. Similarly, apigenin at the same concentration increased Arg-1 and KLF4 expression by 2.21-fold and 1.72-fold, respectively, indicating that both flavonoids were associated with enhanced expression of M2-related markers following suppression of the pro-inflammatory response.

Taken together, the above results indicate that the methanol extract of *T. mongolicum* and its major constituents, luteolin and apigenin, effectively regulate LPS-induced inflammatory responses in RAW264.7. The results obtained from the iNOS and COX-2 analyses demonstrated that the methanol extract, luteolin, and apigenin reduced LPS-induced iNOS and COX-2 protein expression. Notably, the methanol extract exhibited relatively higher TPC and TFC values and showed antioxidant and anti-inflammatory activities, suggesting that its phenolic and flavonoid constituents may be associated with the observed anti-inflammatory effects [70]. Furthermore, luteolin and apigenin were subsequently isolated from the methanol extract, and both compounds effectively suppressed iNOS and COX-2 expression. These findings suggest that flavonoid constituents may represent some of the bioactive components contributing to the anti-inflammatory activity of the methanol extract of *T. mongolicum*.

Luteolin and apigenin, which were identified as major active compounds in the NO inhibition assay, exhibited pronounced inhibitory effects on iNOS expression. This finding was consistent with the observed reduction in NO production, suggesting that these flavonoids may attenuate NO-mediated inflammatory responses through suppression of iNOS expression. In contrast, the inhibitory effects on COX-2 expression were relatively weaker than those observed for iNOS, indicating that luteolin and apigenin may exert differential regulatory effects on distinct inflammation-related enzymes [71]. At the cytokine level, both luteolin and apigenin reduced TNF-α and IL-6 protein expression. Luteolin exhibited inhibitory effects on both TNF-α and IL-6, whereas apigenin showed a more pronounced suppressive effect on TNF-α while also reducing IL-6 expression. These findings indicate that, although both compounds belong to the flavonoid family, they may differ in their regulatory activities toward specific pro-inflammatory cytokines [72].

From the perspective of upstream signaling pathways, NF-κB and MAPK are recognized as major regulators of LPS-induced inflammatory responses in RAW264.7 cells [73]. LPS stimulation promotes IκBα phosphorylation, leading to activation of the NF-κB signaling pathway and subsequent regulation of downstream inflammatory mediators. In addition, the MAPK family, including ERK, JNK, and p38, is involved in the regulation of inflammatory signaling. In the present study, both luteolin and apigenin reduced the phosphorylation levels of IκBα, ERK, JNK, and p38, suggesting that their anti-inflammatory activities may not be limited to suppression of downstream inflammatory proteins, but may also involve inhibition of upstream NF-κB and MAPK signaling activation. Notably, although both luteolin and apigenin suppressed the phosphorylation of MAPK-related proteins, the inhibitory effects on ERK, JNK, and p38 were not identical [74]. Luteolin exhibited more pronounced inhibitory effects on IκBα, JNK, and p38 phosphorylation, suggesting that it may possess broader regulatory activity toward inflammatory signaling pathways. In contrast, apigenin demonstrated relatively stronger suppression of JNK and p38 than of ERK, indicating that it may preferentially regulate JNK/p38 MAPK signaling associated with inflammatory responses. These findings suggest that, although both compounds exhibit anti-inflammatory activity, their regulatory characteristics within the NF-κB and MAPK signaling pathways may differ slightly [75,76]. From a structure–activity relationship (SAR) perspective, these differential regulatory effects may be partially associated with structural differences between luteolin and apigenin. Luteolin possesses an additional 3′-hydroxyl group on the B ring, forming a 3′,4′-dihydroxy moiety, whereas apigenin contains only a 4′-hydroxyl group on the B ring [77]. Previous studies have suggested that hydroxylation patterns within flavonoids may influence their antioxidant capacity and biological activities [78]. Therefore, the broader inhibitory effects of luteolin on IκBα and MAPK phosphorylation observed in the present study may be partially associated with its B-ring hydroxylation pattern.

Finally, regarding macrophage activation-related markers, both luteolin and apigenin increased Arg-1 and KLF4 protein expression while simultaneously reducing iNOS, COX-2, TNF-α, and IL-6 expression [79]. Arg-1 is commonly regarded as an M2-associated marker, whereas KLF4 has been reported to participate in macrophage alternative activation and to cooperate with STAT6 in promoting expression of M2-associated genes such as Arg-1, while suppressing certain M1-related inflammatory targets [80]. Therefore, these findings suggest that, in addition to suppressing pro-inflammatory responses, luteolin and apigenin may also be associated with the modulation of macrophage activation status, accompanied by increased expression of M2-associated immunoregulatory markers [80].

### 3.9. Molecular Docking Analysis

To assess the enzyme-regulating potential of the six compounds isolated from *Taraxacum mongolicum*, computational docking analysis was performed. According to the results summarized in Table 8, apigenin (**4**) showed the most favorable interaction with α-glucosidase, presenting a binding affinity of −8.5 kcal/mol. Affinity was notably lower than that obtained for the reference inhibitor acarbose (−6.9 kcal/mol). Chicoric acid (**1**) and luteolin (**2**) also demonstrated appreciable binding affinities, with calculated docking energies of −8.4 and −8.2 kcal/mol, respectively [81].

For AChE, vanillic acid (**5**) and syringic acid (**6**) showed the strongest decrease potential, both with binding energies of −6.7 kcal/mol, outperforming the positive control chlorogenic acid (−6.4 kcal/mol). These results suggest that phenolic acids possess better structural complementarity and affinity for the AChE catalytic pocket. In comparison, luteolin (**2**) and apigenin (**4**) exhibited slightly weaker affinities (−5.1 and −5.8 kcal/mol, respectively), though their aromatic rings may still stabilize the ligand enzyme complex through π–π stacking and hydrophobic interactions with active-site residues.

Regarding inflammation-related enzymes, all tested constituents exhibited noticeable affinities toward iNOS and COX-2. Among them, luteolin (−9.1 kcal/mol) and apigenin (−8.7 kcal/mol) demonstrated stronger binding than rutin (−8.4 kcal/mol). These findings indicate that flavonoids with planar conjugated and polyhydroxylated structures may adopt favorable binding poses within the catalytic regions of these enzymes and form hydrogen-bonding and hydrophobic interactions with key amino acid residues, which may be relevant to the observed modulation of nitric oxide- and prostaglandin-related pathways.

Overall, the enzyme inhibition data and molecular docking simulations showed generally consistent trends, with docking analysis providing supportive structural insight into possible ligand–target interactions.

**Table 8:** Predicted binding affinity of active components for α-glucosidase, AChE, COX-2, and iNOS.

Compounds	Affinity (kcal/mol) ^A^			
	α-Glucosidase	AChE	iNOS	COX-2
Chicoric acid (**1**)	−8.4	−5.0	−9.3	−8.2
Luteolin (**2**)	−8.2	−5.1	−9.1	−8.8
Caftaric acid (**3**)	−7.5	−6.7	−8.4	−7.7
Apigenin (**4**)	−8.5	−5.8	−8.7	−8.6
Vanillic acid (**5**)	−5.6	−6.7	−6.9	−6.2
Syringic acid (**6**)	−5.7	−6.7	−7.2	−6.5
Acarbose ^B^	−6.9	─	─	─
Chlorogenic acid ^C^	─	−6.4	─	─
Rutin ^D^	─	─	−8.4	−8.1

^A^ Binding affinity value (kcal/mol) indicates the strength and spontaneity of ligand–enzyme interactions; lower values represent stronger binding. ^B^ Acarbose was used as a standard for α-glucosidase. ^C^ Chlorogenic acid was applied as a standard for acetylcholinesterase modulation. ^D^ Rutin was applied as a positive control for inflammation enzyme reduction.

**Figure 10:**
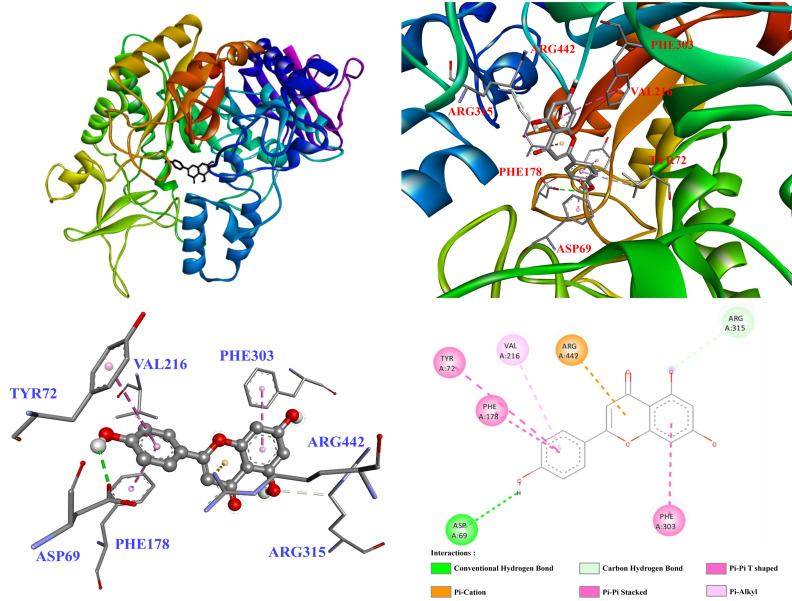
Docking analysis of apigenin with α-glucosidase.

To further elucidate the molecular mechanisms underlying the binding affinities, the interaction modes between representative constituents and their respective target enzymes were analyzed. In the Figure 10, apigenin was tightly embedded in the catalytic pocket through a network of hydrogen bonds and hydrophobic interactions. The ligand interacted with Asp69 and Arg442, key catalytic residues mediating proton transfer and transition-state stabilization [82], while Arg315 and Tyr72 enhanced polar stabilization. Val216, which determines substrate specificity in *S. cerevisiae* α-glucosidase, formed π alkyl contacts that reinforced structural complementarity. In addition, Phe178 and Phe303 established π–π stacking with the flavone ring, further stabilizing the ligand within the hydrophobic cleft. These multi-point interactions collectively positioned apigenin near the catalytic region and may contribute to the observed binding affinity (−8.5 kcal/mol), which could be relevant to the inhibitory activity observed in the enzyme assay. The engagement of Asp69, Arg442, Phe178, and Val216 suggests a potential interaction mode relevant to α-glucosidase inhibition. The positioning of apigenin near the catalytic region supports the possibility that these interactions may contribute to its inhibitory effect.

**Figure 11:**
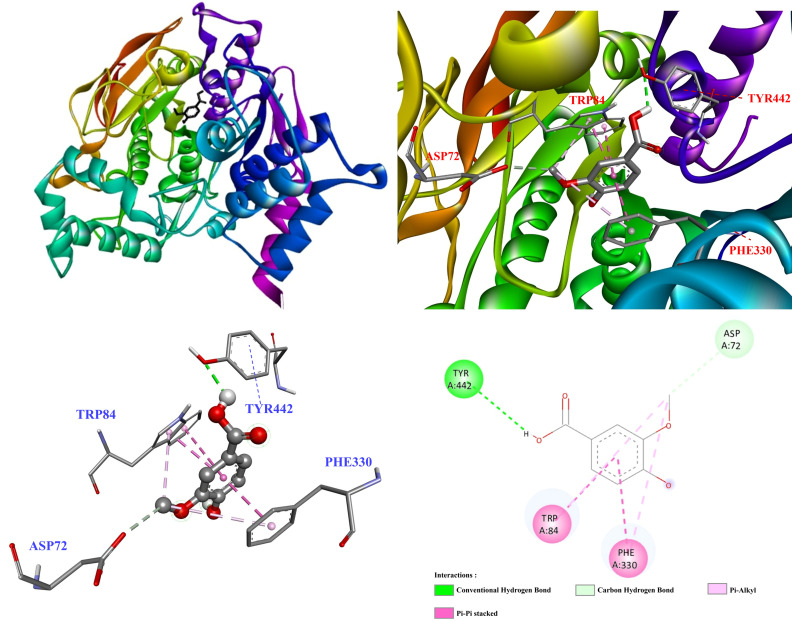
The binding interaction of vanillic acid with AChE was revealed by molecular docking.

In the AChE interaction with vanillic acid (Figure 11), vanillic acid was deeply embedded within the aromatic gorge and stabilized by multiple hydrogen-bonding and π–π stacking interactions. The docking results indicated that vanillic acid interacts with residues located in the peripheral anionic site (PAS), where hydrogen-bonding contacts were observed with Asp72 and Tyr442, residues known to facilitate substrate entry and proper catalytic positioning [83]. In addition, the aromatic ring of vanillic acid was positioned close to Trp84 and Phe330 within the catalytic anionic site (CAS), forming aromatic stacking contacts that contributed to hydrophobic stabilization of the enzyme–ligand complex [84,85]. These dual-site interactions, involving both PAS and CAS residues, are consistent with previously reported binding mechanisms of aromatic modulators [84,85]. Such engagement along the aromatic gorge may influence substrate diffusion and access to the catalytic triad, which is consistent with the observed docking pose and inhibitory potential of vanillic acid toward AChE.

**Figure 12:**
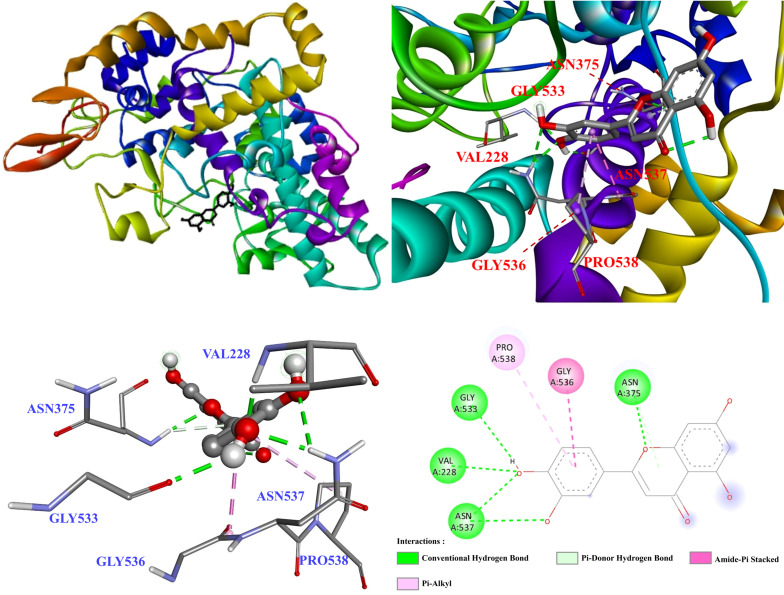
Binding interaction of luteolin with COX-2.

Docking results showed that luteolin was positioned within the hydrophobic pocket of COX-2 (Figure 12). The ligand formed hydrogen bond interactions with residues Asn375, Asn537, Val228, and Gly533, while additional π-related contacts with Gly536 and Pro538 further stabilized the complex. These interactions may help stabilize luteolin near the substrate entry region of COX-2, which may be relevant to its observed binding affinity. This binding pattern supports the possibility that luteolin could interfere with substrate access or positioning and thereby contribute to COX-2 modulation. Notably, the presence of Val228 and Asn375 at the junction of the hydrophobic cleft enhanced the structural complementarity and stability of the complex, consistent with luteolin’s strong affinity toward COX-2. Similar hydrogen-bonding and π–π stacking patterns between flavonoids and COX-2 have been reported previously, indicating that luteolin follows the typical binding framework observed for flavonoid-based COX-2 interacting ligands [86]. These interaction features provide a plausible structural basis for the potential influence of luteolin on COX-2-related prostaglandin biosynthesis.

**Figure 13:**
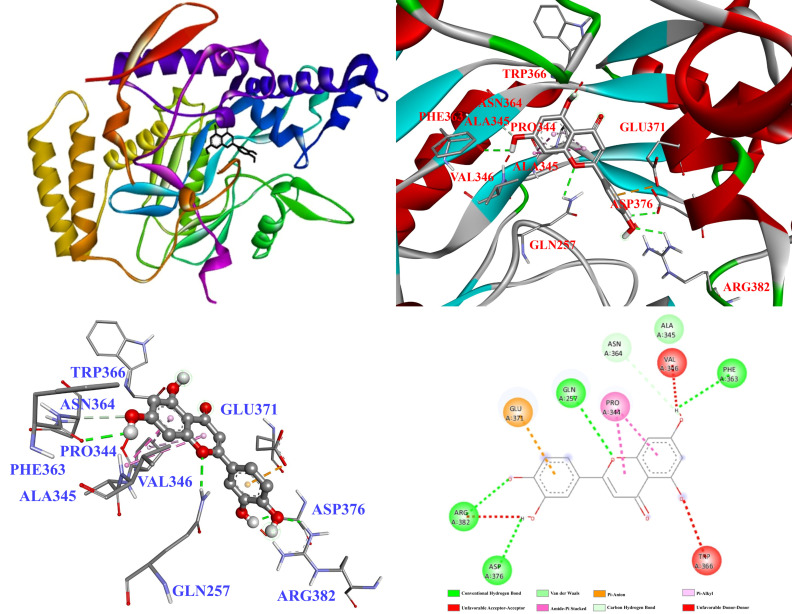
Molecular docking study of luteolin against iNOS.

In iNOS results (Figure 13), luteolin was firmly embedded in the iNOS catalytic pocket through multiple hydrogen bonds with Asp376, Gln257, Phe363, and Arg382, and hydrophobic interactions involving Val346 and Trp366. These residues are located near the heme–BH_4_ cofactor interface, which plays a key role in substrate anchoring and electron transfer during nitric oxide production [87,88]. π-related interaction with Trp366, Pro344 and Glu371 further stabilized the ligand within the heme-binding channel, consistent with the interaction mode observed in selective iNOS-binding ligands such as N-substituted acetamidines and thiourea analogs [87,88]. These stable hydrogen-bonding and aromatic interactions suggest that luteolin may adopt a favorable binding pose within the iNOS catalytic region, including residues located near the heme–BH_4_ microenvironment. Such interactions may be relevant to the observed iNOS modulation.

### 3.10. In Silico Physicochemical Properties Prediction

Key physicochemical characteristics of the major bioactive compounds identified from *Taraxacum mongolicum*, including molecular weight, LogP, PSA, HBD/HBA, pKa, and %ABS, are presented in Table 9. Such descriptors are frequently applied during preliminary drug discovery and physicochemical profiling to enable comparison of molecular properties among candidate compounds [89,90].

With respect to Lipinski’s rule of five, all evaluated compounds exhibited molecular weights below 500 Da, and most constituents fell within the recommended ranges for HBD ≤ 5 and HBA ≤ 10. consistent with commonly applied physicochemical criteria used in early-stage drug discovery [90,91]. Chicoric acid displayed elevated HBD and HBA values, accompanied by a markedly high polar surface area (PSA = 162.60 Å^2^), indicating a physicochemical profile characterized by pronounced polarity. Caftaric acid also exhibited a relatively high PSA value (125.65 Å^2^). These deviations primarily reflect structural features associated with high polarity, which may be associated with reduced passive permeability, but do not necessarily indicate a comprehensive limitation of biological activity [92,93].

In addition to Lipinski parameters, polar surface area is frequently used as an auxiliary indicator for assessing absorption and membrane permeability potential [90]. Previous studies have suggested that PSA values exceeding approximately 140 Å^2^ are associated with a reduced probability of passive diffusion-mediated absorption [94]. In the present analysis, chicoric acid exceeded this reference value, while caftaric acid approached the upper range. In contrast, luteolin (PSA = 89.05 Å^2^) and apigenin (PSA = 73.57 Å^2^) were located within a lower PSA range, indicating distinct differences in polarity-related properties among the compounds.

Regarding absorption-related predictions, %ABS values were estimated based on PSA using a commonly applied empirical equation (%ABS = 109 − 0.345 × PSA), which is frequently employed to provide a relative comparison of absorption potential among compounds [76]. Based on this estimation, chicoric acid exhibited a lower predicted %ABS value (52.90%), whereas luteolin, apigenin, vanillic acid, and syringic acid showed comparatively higher predicted values. The overall trend was consistent with the distribution of PSA values, with compounds of higher polarity corresponding to lower predicted absorption percentages and those with lower PSA exhibiting relatively higher values [91].

In terms of ionization characteristics, predicted pKa values indicated that vanillic acid (pKa = 5.12) and syringic acid (pKa = 5.30) may exist in partially ionized forms under physiological pH conditions, providing a physicochemical basis for their distribution between aqueous and lipid phases [95]. In contrast, chicoric acid and caftaric acid exhibited stronger acidic characteristics. These pKa values are presented to describe potential ionization tendencies and do not imply specific in vivo behavior.

Furthermore, drug-likeness scores varied among the evaluated compounds [96], with luteolin and apigenin displaying positive values, while highly polar phenolic acids showed lower or negative scores. These scores are intended to serve as supportive physicochemical indicators for relative comparison rather than as predictors of biological efficacy or pharmacological potency. Overall, the physicochemical and drug-screening-related parameters presented in this study indicate that the major bioactive constituents of *T. mongolicum* span a broad range of polarity, lipophilicity, and absorption-related characteristics.

Overall, by integrating extract-level screening, compound-level evaluation, mechanistic cellular analysis, and supportive in silico analyses, this study demonstrates that *T. mongolicum* is a promising source of multifunctional bioactive constituents. The observed antioxidant, enzyme-inhibitory, anti-inflammatory, and immunomodulatory effects, particularly those associated with luteolin and apigenin, provide a useful basis for further investigation of their biological significance and future application potential.

## 4. Conclusions

This study comprehensively explores the bioactive potential of *Taraxacum mongolicum* using solvent extraction, compound identification, enzymatic assays, cell-based models, and mechanistic analyses. Among the extracts, the methanol fraction showed the strongest antioxidant activity, inhibition of inflammation-related enzymes, and regulation of NO production, suggesting enrichment of bioactive phenolic acids and flavonoids. Comparative evaluation of six major constituents revealed distinct functional roles; chicoric acid exhibited strong antioxidant activity, while luteolin and apigenin showed prominent anti-inflammatory effects. Syringic acid and vanillic acid displayed relatively stronger AChE inhibitory activity. In the cell model, the methanol extract, luteolin, and apigenin significantly suppressed NO production and decreased the expression of iNOS and COX-2, accompanied by a decrease in cytokines. Mechanistic analysis indicated that luteolin inhibited inflammatory signaling through attenuation of the NF-κB pathway together with modulation of the MAPK cascade, whereas apigenin primarily acted through NF-κB inhibition. Both compounds also enhanced the expression of M2 macrophage markers, suggesting potential roles in regulating immune homeostasis. Overall, these findings support the value of *T. mongolicum* as a promising source of multifunctional bioactive constituents and worth further in vivo validation and translational studies.

## Figures and Tables

**Table 1:** TPC, TFC, and yield of *Taraxacum mongolicum* extracts.

Solvent Extracts	TPC (mg/g) ^A^	TFC (mg/g) ^B^	Yields (%) ^C^
*n*-Hexane	<1	<1	1.65
Dichloromethane	<1	13.03 ± 4.72 ^b^	2.10
EtOAc	14.98 ± 1.35 ^a^	13.54 ± 3.27 ^b^	2.90
Acetone	14.93 ± 2.44 ^a^	12.57 ± 2.63 ^b^	3.10
EtOH	9.92 ± 3.54 ^a^	4.09 ± 1.52 ^a^	6.70
MeOH	42.59 ± 3.10 ^b^	26.63 ± 1.89 ^c^	12.45
Water	12.28 ± 4.70 ^a^	4.03 ± 1.95 ^a^	14.50
Water (100 °C)	32.35 ± 5.74 ^b^	7.13 ± 1.31 ^ab^	30.25

^A^ TPC is presented as mg GAE per g of extract. ^B^ TFC is expressed as mg quercetin equivalents (QE) per g extract. ^C^ Yield (%) was calculated as (extract weight/initial dry sample weight) × 100%. Values represent mean ± SD (n = 3). Different letters (a–c) indicate significant differences among groups (*p* < 0.05, Tukey’s test).

**Table 2:** Assessment of the antioxidant capacity of *Taraxacum mongolicum* extracts.

Solvent Extracts	SC_50_ (μg/mL) ^A^			TE (mM/g) ^B^
	DPPH	ABTS	Superoxide	FRAP
*n*-Hexane	>400	>400	>400	<1
Dichloromethane	>400	>400	>400	<1
Ethyl acetate	>400	>400	>400	21.17 ± 6.19 ^a^
Acetone	>400	>400	>400	22.80 ± 11.90 ^a^
EtOH	342.36 ± 23.17 ^d^	272.77 ± 69.56 ^b^	>400	112.74 ± 12.71 ^a^
MeOH	75.40 ± 2.30 ^a^	74.75 ± 5.52 ^a^	>400	625.67 ± 9.19 ^b^
Water	242.57 ± 2.34 ^c^	251.29 ± 16.39 ^b^	193.77 ± 4.23 ^c^	119.63 ± 14.07 ^a^
Water (100 °C)	114.37 ± 1.91 ^b^	117.32 ± 19.14 ^a^	84.04 ± 2.22 ^b^	229.27 ± 0.41 ^a^
BHT ^C^	118.46 ± 0.69 ^b^	8.57 ± 0.34 ^a^	─	2135.78 ± 189.57 ^c^
Cynaroside ^D^	─	─	17.10 ± 0.72 ^a^	─

^A^ SC_50_ denotes the concentration required to scavenge 50% of radicals. ^B^ FRAP values are reported as mM TE per g extract. ^C^ BHT served as the reference antioxidant in the DPPH, ABTS, and FRAP assays. ^D^ Cynaroside was used as the reference compound in the superoxide scavenging assay. Data are expressed as mean ± SD (n = 3). Different letters (a–d) indicate significant differences among groups (*p* < 0.05, Tukey’s test).

**Table 3:** Enzyme suppression activities of *Taraxacum mongolicum* extracts.

Solvent Extracts	IC_50_ (μg/mL) ^A^	
	α-Glucosidase	AChE
*n*-Hexane	176.73 ± 13.13 ^a^	85.01 ± 5.16 ^a^
Dichloromethane	257.68 ± 49.19 ^ab^	68.91 ± 7.50 ^a^
Ethyl acetate	347.61 ± 44.91 ^bc^	68.63 ± 7.12 ^a^
Acetone	582.99 ± 48.36 ^ef^	73.91 ± 8.53 ^a^
EtOH	437.88 ± 7.34 ^cd^	317.66 ± 31.02 ^c^
MeOH	490.60 ± 0.46 ^de^	223.71 ± 27.63 ^b^
Water	>800	72.73 ± 7.73 ^a^
Water (100 °C)	641.97 ± 76.96 ^f^	97.48 ± 4.45 ^a^
Acarbose ^B^	460.71 ± 27.80 ^cd^	─
Chlorogenic acid ^C^	─	82.28 ± 6.48 ^a^

^A^ IC_50_ indicates the concentration required to inhibit 50% of enzyme activity. ^B^ Acarbose was used as the positive control for α-glucosidase inhibition. ^C^ Chlorogenic acid was used as the standard for AChE modulation. Data are presented as mean ± SD (n = 3). Different letters (a–f) indicate significant differences among groups (*p* < 0.05, Tukey’s test).

**Table 4:** NO inhibitory activities of *Taraxacum mongolicum* solvent extracts in LPS-induced RAW264.7.

Solvent Extracts	IC_50_ (μg/mL) ^A^
	NO Inhibition
*n*-Hexane	19.14 ± 0.63 ^a^
Dichloromethane	19.48 ± 2.11 ^a^
Ethyl acetate	16.17 ± 0.91 ^a^
Acetone	23.05 ± 1.14 ^a^
EtOH	28.22 ± 0.85 ^a^
MeOH	15.61 ± 1.60 ^a^
Water	71.50 ± 12.3 ^b^
Water (100 °C)	25.73 ± 0.97 ^a^
Rutin ^B^	30.45 ± 4.90 ^a^

^A^ IC_50_ indicates the concentration required for 50% inhibition. ^B^ Rutin served as the reference inhibitor in the assay. Values are presented as mean ± SD (n = 3). Different letters (a,b) indicate significant differences among groups (*p* < 0.05, Tukey’s test).

**Table 7:** NO inhibitory activities of *Taraxacum mongolicum* isolated components.

Compounds	IC_50_ (μM) ^A^
	NO Inhibition
Chicoric acid (**1**)	14.21 ± 0.75 ^ab^
Luteolin (**2**)	9.04 ± 0.14 ^a^
Caftaric acid (**3**)	26.44 ± 4.47 ^c^
Apigenin (**4**)	9.81 ± 0.82 ^a^
Vanillic acid (**5**)	18.95 ± 0.39 ^b^
Syringic acid (**6**)	12.76 ± 0.12 ^ab^
Rutin ^B^	19.10 ± 1.92 ^b^

^A^ IC_50_ represents the concentration required to achieve 50% inhibition ratio. All values were presented as mean ± SD (n = 3). ^B^ Rutin as a positive control in this assay. Different letters (a–c) indicate statistically significant differences among groups (*p* < 0.05, Tukey’s test).

**Table 9:** Predicted physicochemical characteristics and bioavailability-related properties of major components isolated from *Taraxacum mongolicum*.

Compounds	LogP	MW	HBA	HBD	Drug-Likeness Score ^C^	PSA	pKa of Most Basic/Acidic Group ^A^	%ABS ^B^
Chicoric Acid (**1**)	1.12	474.08	12	6	−0.23	162.60 Å^2^	<0/3.09	52.90
Luteolin (**2**)	2.78	286.05	6	4	0.38	89.05 Å^2^	<0/6.70	78.28
Caftaric Acid (**3**)	−0.85	312.05	9	5	−0.02	125.65 Å^2^	<0/2.58	65.65
Apigenin (**4**)	3.22	270.05	5	3	0.39	73.57 Å^2^	<0/6.70	83.61
Vanillic Acid (**5**)	1.20	168.04	4	2	−0.18	52.83 Å^2^	<0/5.12	90.77
Syringic Acid (6)	0.82	198.05	5	2	−0.81	59.39 Å^2^	<0/5.30	88.51

^A^ pKa values indicate predicted ionization tendencies of the most acidic or basic functional groups. ^B^ %ABS was estimated from PSA using the empirical equation %ABS = 109 − 0.345 × PSA and is applied for relative, screening-level comparison of absorption potential. ^C^ Drug-likeness scores represent property-based heuristic indicators and are intended for comparative physicochemical assessment rather than prediction of biological efficacy or in vivo behavior.

## Data Availability

The original contributions presented in this study are included in the article/Supplementary Materials. Further inquiries can be directed to the corresponding author.

## Supplementary Materials

The following supporting information can be downloaded at: https://www.mdpi.com/article/10.3390/antiox15060688/s1, Figures S1–S4: ^1^H-NMR, MS, purity profiles and IR spectrum of compound **1**; Figures S5–S8: ^1^H-NMR, MS, purity profiles and IR spectrum of compound **2**; Figures S9–S12: ^1^H-NMR, MS, purity profiles and IR spectrum of compound **3**; Figures S13–S16: ^1^H-NMR, MS, purity profiles and IR spectrum of compound **4**; Figures S17–S20: ^1^H-NMR, MS, purity profiles and IR spectrum of compound **5**; Figures S21–S24: ^1^H-NMR, MS, purity profiles and IR spectrum of compound **6**; Figure S25: Molecular docking interactions of Chicoric acid with α-glucosidase; Figure S26: Molecular docking interactions of Luteolin with α-glucosidase; Figure S27: Molecular docking interactions of Acarbose with α-glucosidase; Figure S28: Molecular docking interactions of Syringic acid with AchE; Figure S29: Molecular docking interactions of Cholorgenic acid with AchE; Figure S30: Molecular docking interactions of Apigenin with iNOS; Figure S31: Molecular docking interactions of Rutin with iNOS; Figure S32: Molecular docking interactions of Apigenin with COX-2; Figure S33: Molecular docking interactions of Rutin with COX-2. Primary antibody information, Table S1: Primary antibodies used for Western blot analysis; Figure S34: Original uncropped Western blot images corresponding to Figure 5A–F; Figure S35: Original uncropped Western blot images corresponding to Figure 6A–D; Figure S36: Original uncropped Western blot images corresponding to Figure 7A–D; Figure S37: Original uncropped Western blot images corresponding to Figure 8A–D; Figure S38: Original uncropped Western blot images corresponding to Figure 9A–D.

## Abbreviations

The following abbreviations are used in this manuscript:

NO	Nitric oxide
ROS	Reactive oxygen species
RNS	Reactive nitrogen species
GRAS	Generally Recognized as Safe
FDA	Food and Drug Administration
^1^H-NMR	Nuclear magnetic resonance
Hz	Hertz
IR	Infrared spectrophotometer
TLC	Thin-layer chromatography
PTLC	Preparative thin-layer chromatography
ESI-MS	Electrospray ionization mass spectrometry
CC	Column chromatography
Fr	Fraction
R*_f_*	Retardation factor
M	Methanol
E	Ethyl acetate
FA	Formic acid
H	*n*-Hexane
TFC	Total Flavonoid Content
TPC	Total Phenolic Content
DPPH	1,1-diphenyl-2-picrylhydrazyl
ABTS	2,2′-azinobis-3-ethyl benzothiazoline-6-sulphonic acid
FRAP	Ferric Reducing Antioxidant Power
AChE	Acetylcholinesterase
MTT	3-(4,5-dimethylthiazol-2-yl)-2,5-diphenyltetrazolium
NaNO_2_	Sodium nitrite
ECL	enhanced chemiluminescence
MW	molecular weight
logP	Lipophilicity
PSA	Polar surface area
SD	Standard deviation
QE	quercetin equivalents
SET	Single electron transfer
PAS	Peripheral anionic site
DPBS	Dulbecco’s phosphate-buffered saline
DCM	dichloromethane
KLF4	Krüppel-Like Factor 4
HPLC	high-performance liquid chromatography
LPS	Lipopolysaccharide
CAS	atalytic anionic site
