# Supplementary material for: Bioactive Extracts and Constituents from Taraxacum mongolicum: Antioxidant, Anti-Inflammatory, Enzyme-Inhibitory, and Molecular Docking Studies
Source: Antioxidants (Basel). 2026 May 29;15(6):688. doi: 10.3390/antiox15060688 (PMC13295379; doi:10.3390/antiox15060688)
Supplement: Supplementary file 1 [file antioxidants-15-00688-s001.zip › antioxidants-4302915-supplementary.pdf]

Bioactive Extracts and Constituents from *Taraxacum mongolicum*: Antioxidant, Anti-inflammatory, Enzyme-Inhibitory, and Molecular Docking Studies.

Kuan-Ying Huang [a], Sin-Min Li [a], and Jih-Jung Chen [a,b,c,*]

[a] Department of Pharmacy, School of Pharmaceutical Sciences, National Yang Ming Chiao Tung University, Taipei 112304, Taiwan

[b] Department of Medical Research, China Medical University Hospital, China Medical University, Taichung 404333, Taiwan

[c] Traditional Herbal Medicine Research Center, Taipei Medical University Hospital, Taipei 110301, Taiwan

[*] Corresponding author

E-mail address: jjungchen@nycu.edu.tw Tel.: +886-2-2826-7195; Fax: +886-2-2823-2940

Contents

Spectra data of isolated compounds……S3

^{1}H-NMR, ESI-MS, HPLC (purity profile), and IR spectra of compound **1**……S5

^{1}H-NMR, ESI-MS, HPLC (purity profile), and IR spectra of compound **2**……S7

^{1}H-NMR, ESI-MS, HPLC (purity profile), and IR spectra of compound **3**…… S10

^{1}H-NMR, ESI-MS, HPLC (purity profile), and IR spectra of compound **4**…… S12

^{1}H-NMR, ESI-MS, HPLC (purity profile), and IR spectra of compound **5**……S14

^{1}H-NMR, ESI-MS, HPLC (purity profile), and IR spectra of compound **6**……S16

Molecular docking interactions of Chicoric acid with α-glucosidase…… S19

Molecular docking interactions of Luteolin with α-glucosidase……S20

Molecular docking interactions of Acarbose with α-glucosidase……S21

Molecular docking interactions of Syringic acid with AChE……S22

Molecular docking interactions of Cholorgenic acid with AChE……S22

Molecular docking interactions of Apigenin with iNOS……S23

Molecular docking interactions of Rutin with iNOS……S24

Molecular docking interactions of Apigenin with COX-2……S25

Molecular docking interactions of Rutin with COX-2……S26

Primary antibody information……S27

Original uncropped Western blot images corresponding to Figure 5A–F……S28

Original uncropped Western blot images corresponding to Figure 6A–D……S29

Original uncropped Western blot images corresponding to Figure 7A–D……S30

Original uncropped Western blot images corresponding to Figure 8A–D……S30

Original uncropped Western blot images corresponding to Figure 9A–D……S31

Spectrum data of isolated compounds

Chicoric acid (**1**)

Yellow powder (water), m.p. 205–209 °C; UV (MeOH) λ_{max} (log ε): 216 (4.46), 240 (4.29), 300 (sh, 4.42), 328 (4.54) nm; ^{1}H-NMR (acetone-d_6, 400 MHz) δ 5.88 (2H, s, H-2 and H-3), 6.39 (2H, d, J = 15.9 Hz, H-8′ and H-8″), 6.89 (2H, d, J = 8.2 Hz, H-5′ and H-5″), 7.11 (2H, dd, J = 8.2, 2.0 Hz, H-6′ and H-6″), 7.22 (2H, d, J = 2.0 Hz, H-2′ and H-2″), 7.65 (2H, d, J = 15.9 Hz, H-7′ and H-7″); IR (ATR) 3402, 3336 (OH), 3300–2500 (OH stretching vibration of the carboxyl group), 1716, 1678 (C=O) cm^{-1}; ESI-MS *m/z* 473 [M−H]$^-$.

Luteolin (**2**)

Yellow powder (MeOH), m.p. 328–330 °C; UV (MeOH) λ_{max} (log ε): 252 (4.14), 346 (4.19) nm; ^{1}H-NMR (400 MHz, acetone-d_6) δ 6.23 (1H, d, J = 2.1 Hz, H-6), 6.50, (1H, d, J = 2.1 Hz, H-8), 6.56 (1H, s, H-3), 6.98 (1H, d, J = 8.3 Hz, H-5′), 7.45 (1H, dd, J = 8.3, 2.1 Hz, H-6′), 7.48 (1H, d, J = 2.1 Hz, H-2′), 12.99 (1H, brs, OH-5); IR (ATR) 3415 (OH), 1653 (conjugated C=O), 1608, 1514, 1454 (armatic C=C stretch) cm^{-1}; ESI-MS *m/z* 285 [M−H]$^-$.

Caftaric acid (**3**)

Yellow powder (water), m.p. 124–125 °C; UV (MeOH) λ_{max} (log ε): 216 (4.13), 242 (3.98), 328 (4.25) nm; ^{1}H-NMR (400 MHz, CD_3OD) δ 4.78 (1H, d, J = 2.4 Hz, H-3′), 5.55 (1H, d, J = 2.4 Hz, H-1′), 6.31 (1H, d, J = 15.9 Hz, H-8), 6.78 (1H, d, J = 8.2 Hz, H-5), 6.97 (1H, dd, J = 8.2, 2.0 Hz, H-6), 7.07 (1H, d, J = 2.0, H-2), 7.67 (1H, d, J = 15.9, H-7); IR (ATR) 3481, 3211 (OH), 3300–2500 (OH stretching vibration of the carboxyl group), 1755, 1705 (C=O), 1614, 1517, 1473 (armatic C=C stretch) cm^{-1}; ESI-MS *m/z* 311 [M−H]$^-$.

Apigenin (**4**)

Light yellowish powder (MeOH), m.p. 346–347 °C; UV (MeOH) λ_{max} (log ε): 266 (4.24), 333 (4.29) nm; ^{1}H-NMR (400 MHz, acetone-d_6) δ 6.24 (1H, d, J = 1.6 Hz, H-6), 6.53 (1H, d, J = 1.6 Hz, H-8), 6.62 (1H, s, H-3),

7.01 (2H, d, *J* = 8.6 Hz, H-3′ and H-5′), 7.92 (2H, d, *J* = 8.6 Hz, H-2′ and H-6′), 9.43 (1H, br s, OH-7), 12.99 (1H, br s, OH-5); IR (ATR) 3277 (OH), 1651 (conjugated C=O), 1602, 1587, 1494 (armatic C=C stretch) cm^{-1}; ESI-MS *m/z* 269 $[M-H]^-$.

Vanillic acid (**5**)

White powder (water), m.p. 210–211 °C; UV (MeOH) λ_{max} (log ε): 256 (3.86), 289 (3.57) nm; ^{1}H-NMR (400 MHz, acetone-d_6) δ 3.89 (1H, s, OMe-3), 6.89 (1H, d, *J* = 8.2 Hz, H-5), 7.55 (1H, d, *J* = 1.9 Hz, H-2), 7.58 (1H, dd, *J* = 8.2, 1.9 Hz, H-6); IR (ATR) 3477 (OH), 3300–2500 (OH stretching vibration of the carboxyl group), 1668 (conjugated C=O), 1595, 1519, 1454 aromatic C=C stretch) cm^{-1}; ESI-MS *m/z* 167 $[M-H]^-$.

Syringic acid (**6**)

White powder (MeOH) m.p. 206–208 °C; UV (MeOH) λ_{max} (log ε): 214 (4.29), 271 (3.91) nm; ^{1}H-NMR (400 MHz, $CDCl_3$) δ 3.96 (6H, s, OMe-3 and OMe-5), 5.98 (1H, br s, OH-4), 7.39 (2H, s, H-2 and H-6); IR (ATR) 3363 (OH), 3300–2500 (OH stretching vibration of the carboxyl group), 1693 (C=O), 1614, 1519, 1456 (aromatic C=C stretch) cm^{-1}; ESI-MS *m/z* 197 $[M-H]^-$.

MS, IR, and ^{1}H-NMR spectra of isolated compounds

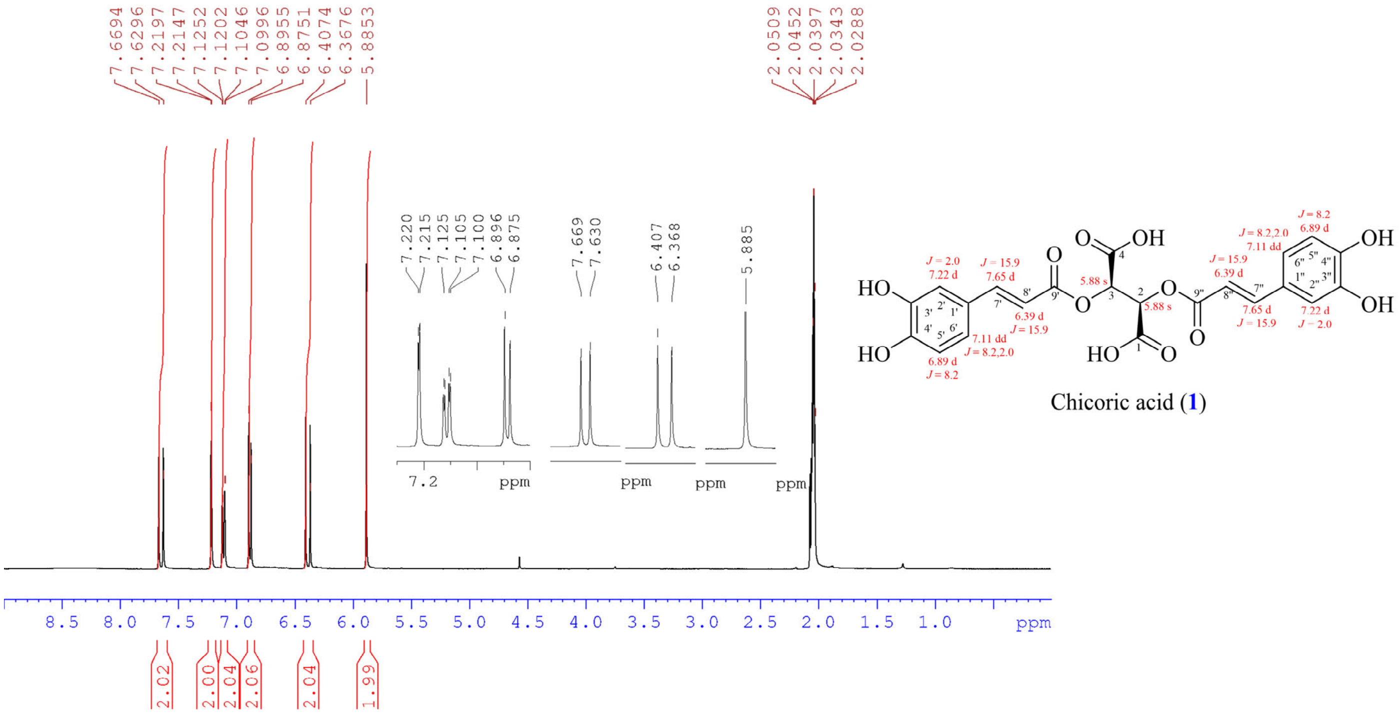

Figure S1. ^{1}H-NMR spectrum of Chicoric acid (**1**).

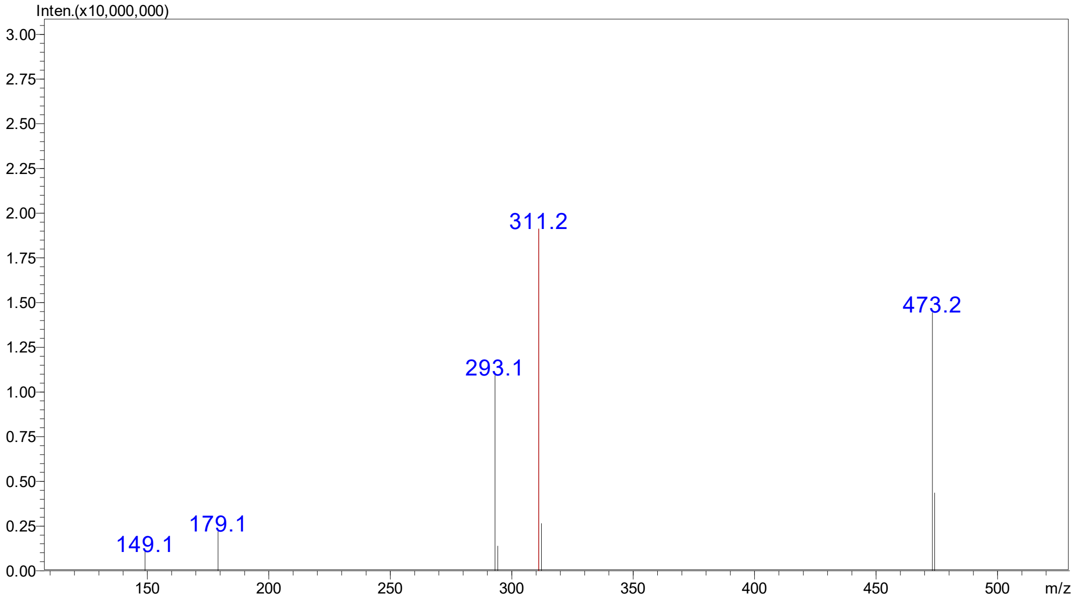

Figure S2. ESI-MS spectrum of Chicoric acid (**1**).

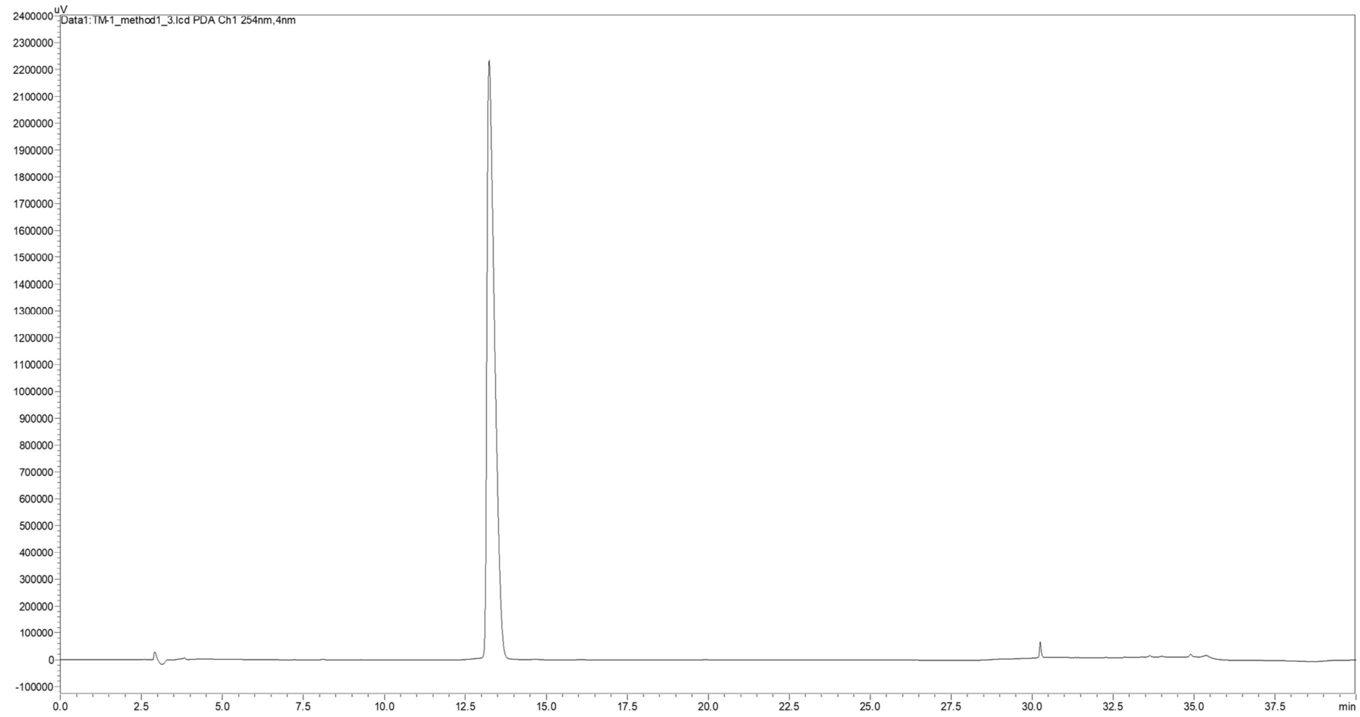

Results View - Peak Table

Peak Table | Compound | Group | Calibration Curve

Peak#	Ret. Time	Area	Height	Mark	Conc.	Area%
1	2.906	189777	32176	M	0.000	0.510
2	3.525	75409	-4	M	0.000	0.203
3	3.822	10948	5099	M	0.000	0.029
4	13.237	36462110	2230690	M	0.000	97.943
5	30.252	229260	60362	M	0.000	0.616
6	33.633	70385	6314	M	0.000	0.189
7	34.897	190031	9513	M	0.000	0.510
Total		37227922	2344150		0.000	100.000

Figure S3. purity profile of Chicoric acid (**1**).

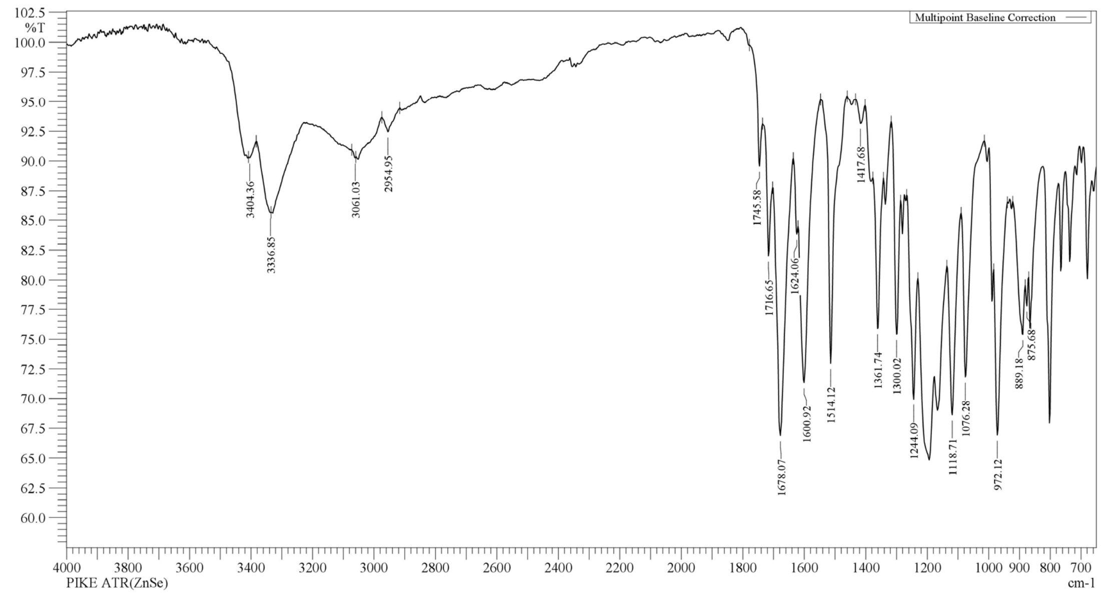

Figure S4. IR spectrum of Chicoric acid (**1**).

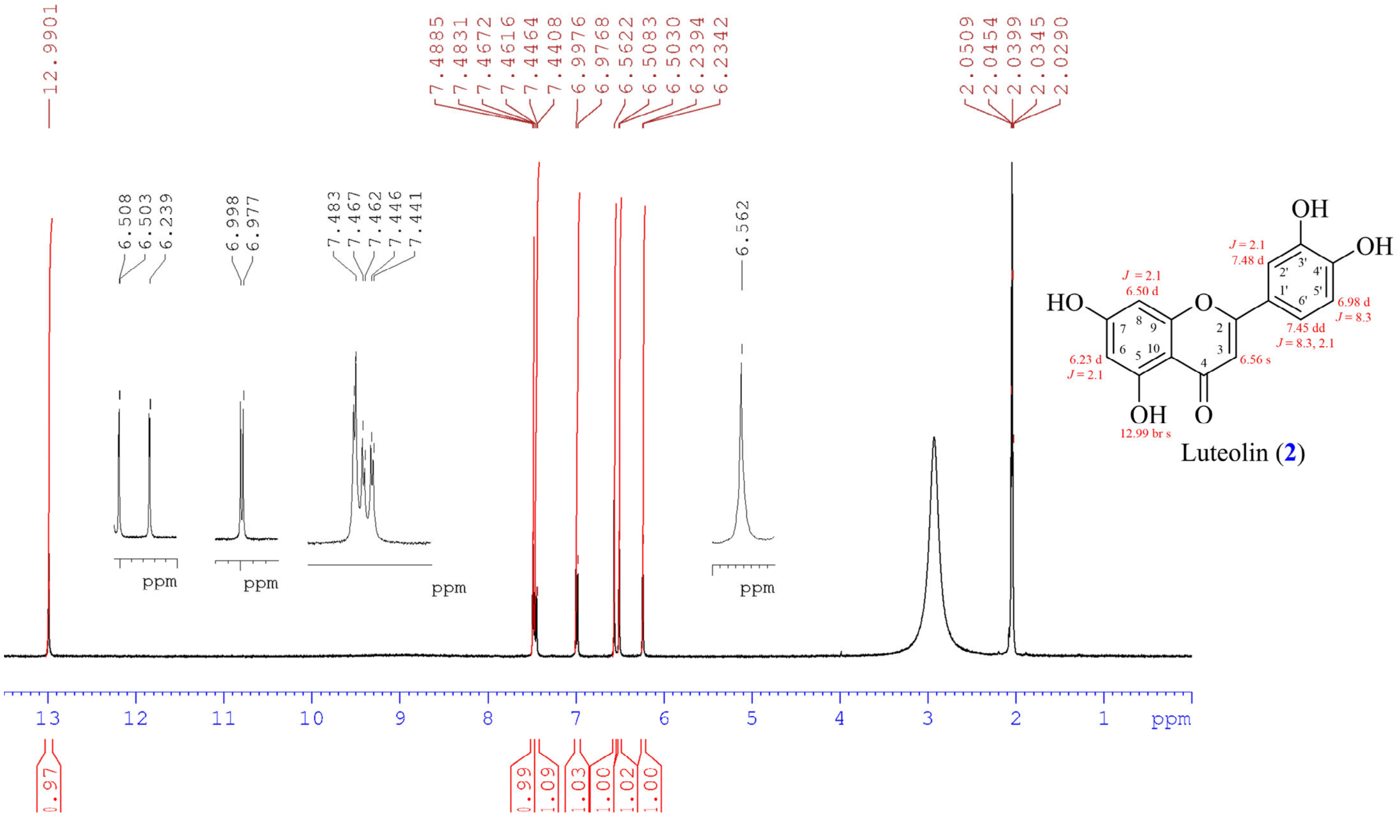

Figure S5. ^{1}H-NMR spectrum of Luteolin (**2**).

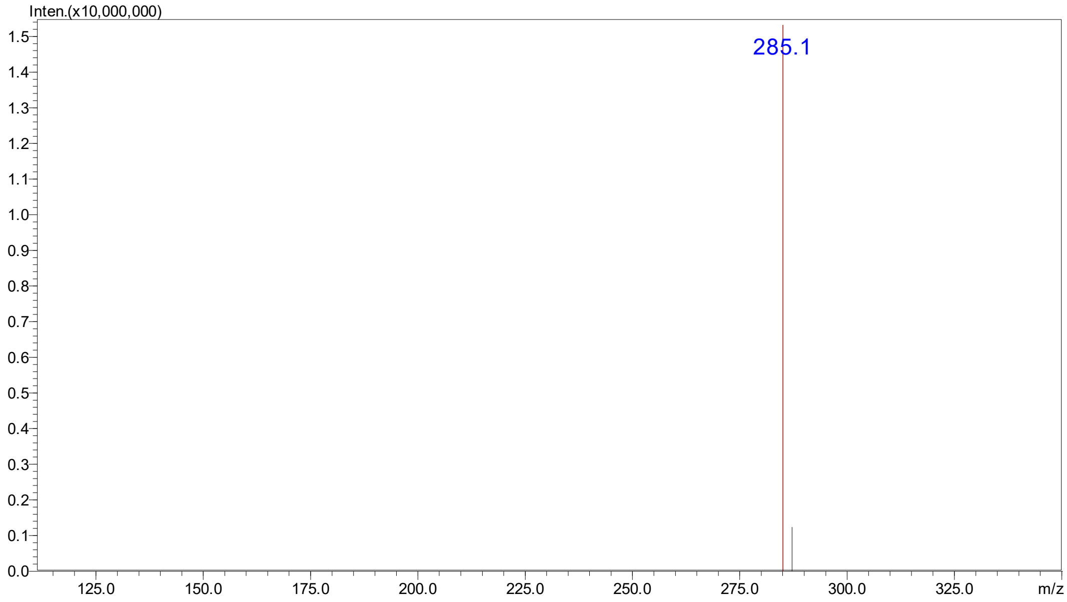

Figure S6. ESI-MS spectrum of Luteolin (**2**).

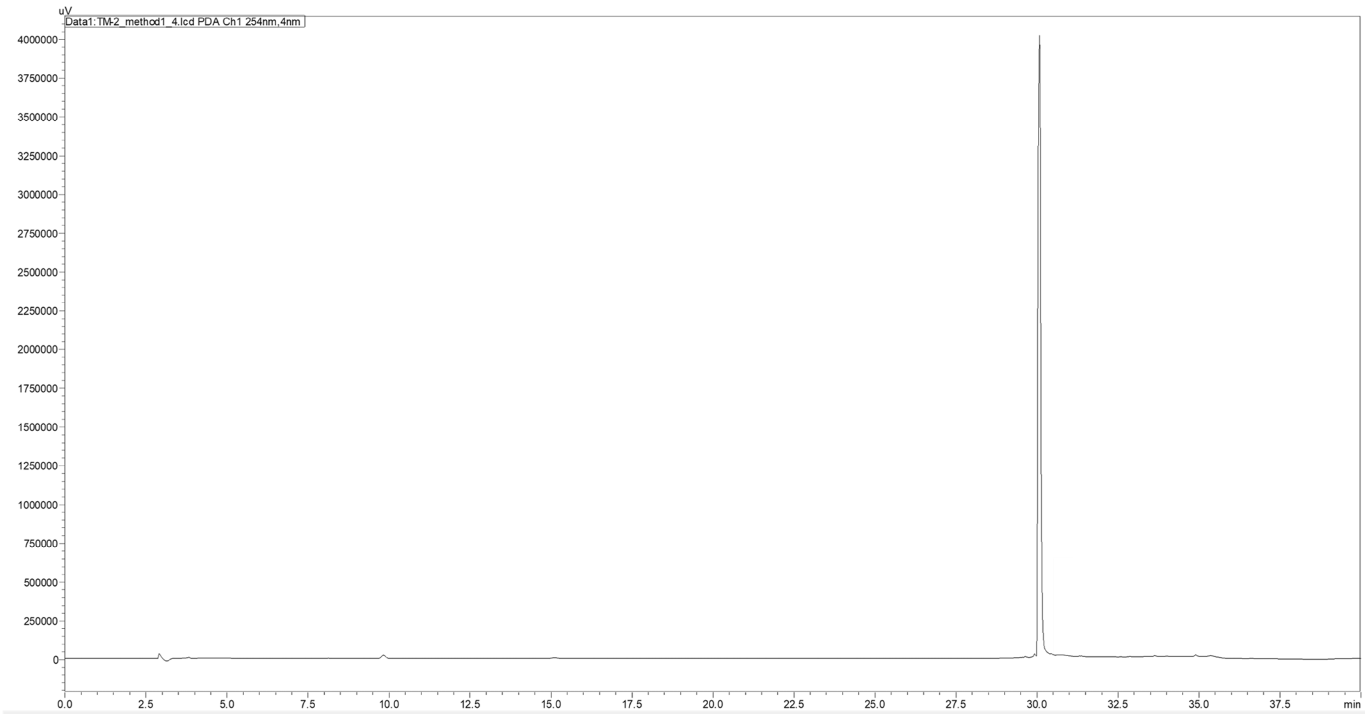

Peak Table | Compound | Group | Calibration Curve

Peak#	Ret. Time	Area	Height	Mark	Conc.	Area%
1	2.906	21339	28156	M	0.000	0.091
2	9.830	176711	19996	M	0.000	0.754
3	15.110	63149	5150	M	0.000	0.269
4	29.644	14602	4662	M	0.000	0.062
5	29.930	32372	11185	M	0.000	0.138
6	30.080	22908250	3840825	M	0.000	97.720
7	31.373	27943	4617	M	0.000	0.119
8	32.866	13474	2280	M	0.000	0.057
9	33.633	22249	5272	M	0.000	0.095
10	34.897	44045	8910	M	0.000	0.188
11	35.366	102458	7407	M	0.000	0.437
12	36.623	16118	2394	M	0.000	0.069
Total		23442709	3940855		0.000	100.000

Figure S7. purity profile of luteolin (**2**).

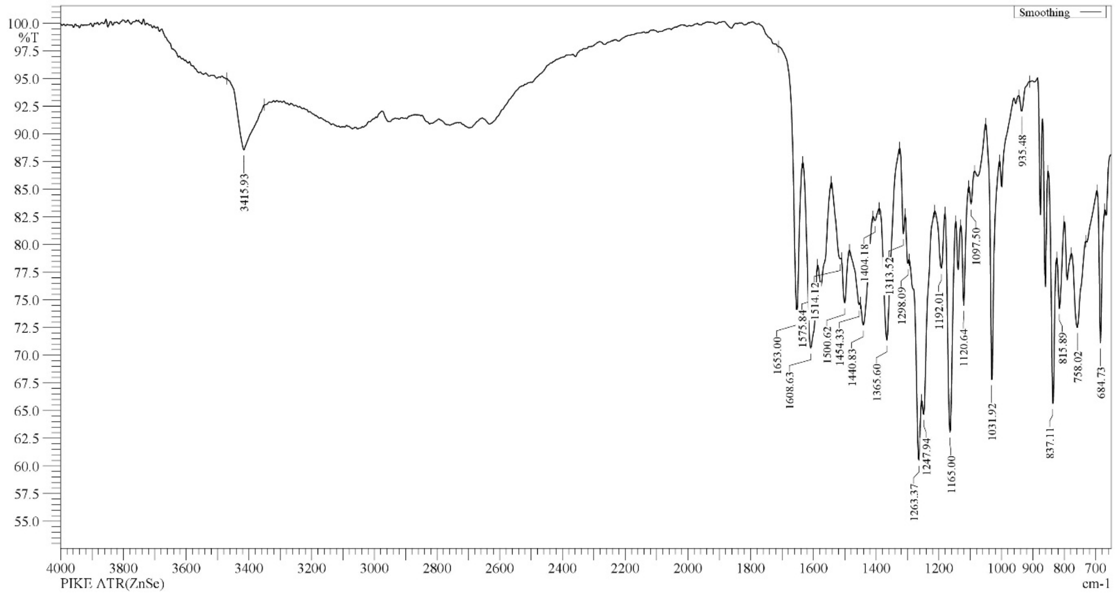

Figure S8. IR spectrum of Luteolin (**2**).

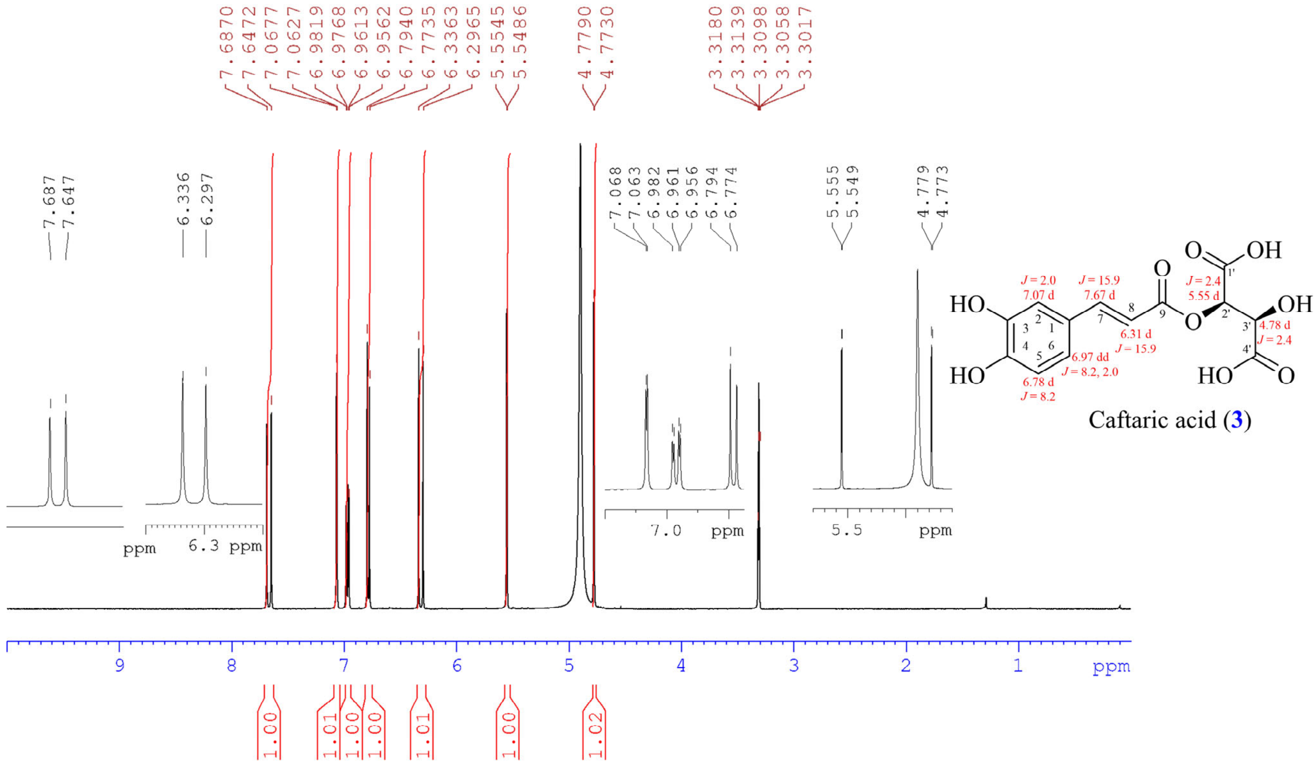

Figure S9. ^{1}H-NMR spectrum of Caftaric acid (**3**).

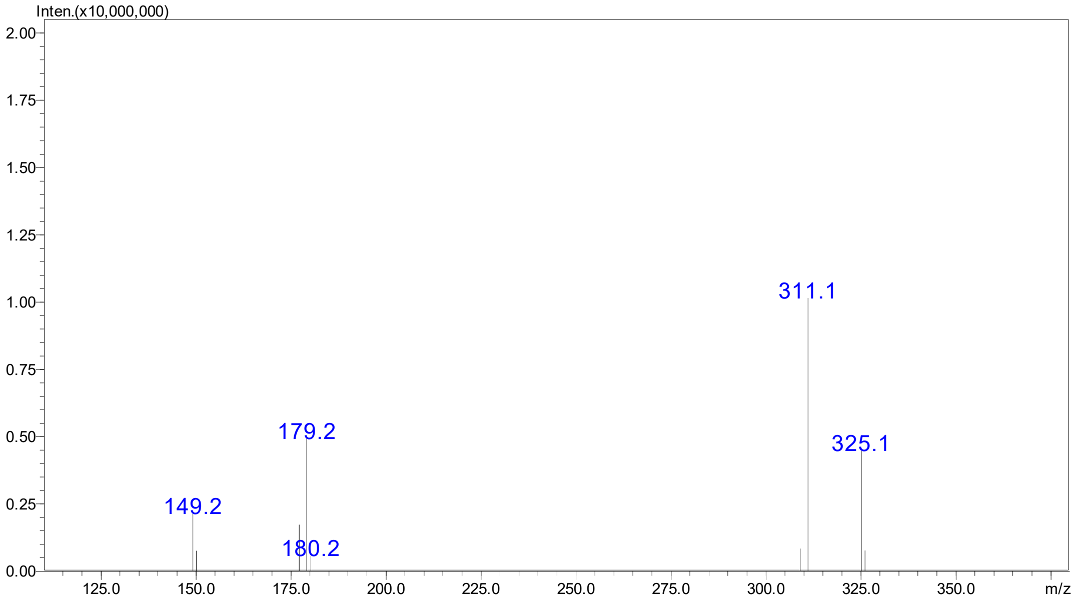

Figure S10. ESI-MS spectrum of Caftaric acid (**3**).

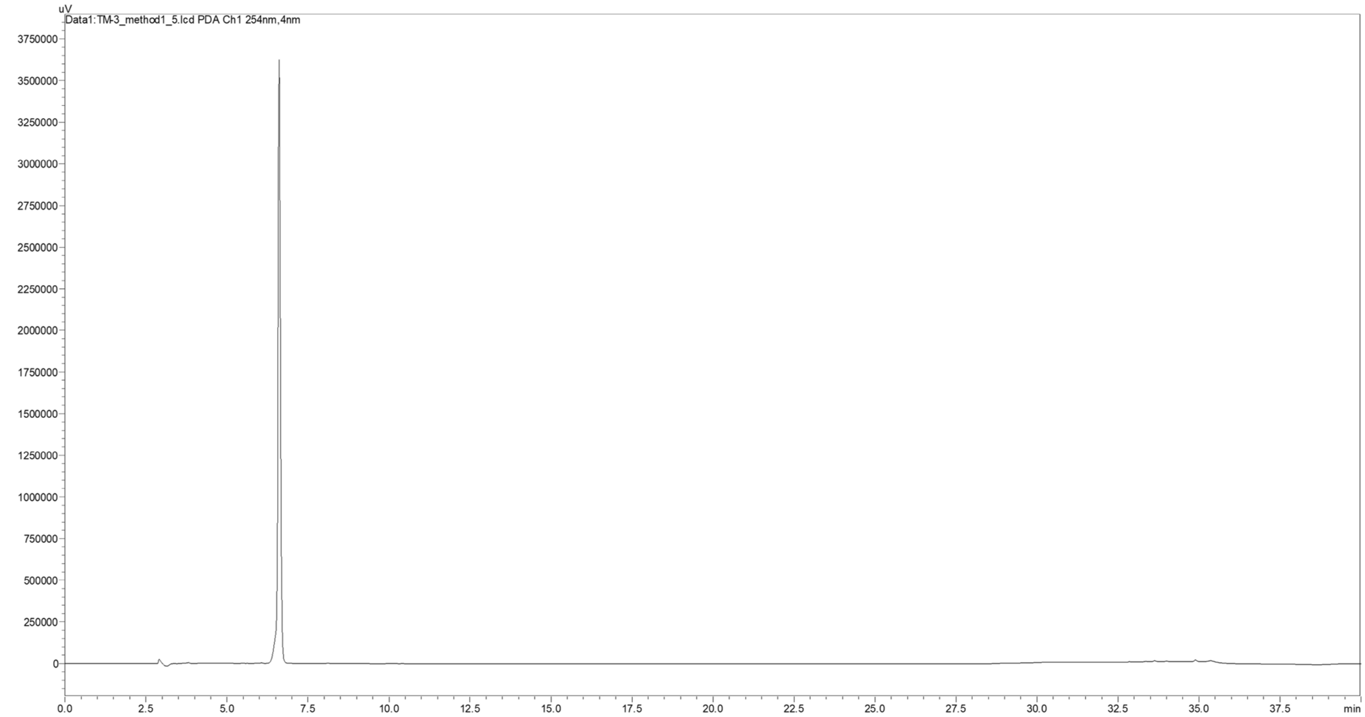

Peak Table | Compound | Group | Calibration Curve

Peak#	Ret. Time	Area	Height	Mark	Conc.	Area%
1	2.905	237458	32290	M	0.000	1.396
2	3.818	11838	4764	M	0.000	0.070
3	6.062	11897	3640	M	0.000	0.070
4	6.611	16661778	3433718	M	0.000	97.968
5	8.110	16987	3162	M	0.000	0.100
6	14.039	36	10	M	0.000	0.000
7	32.859	-1554	1789	M	0.000	-0.009
8	34.892	69003	8856	M	0.000	0.406
Total		17007442	3488228		0.000	100.000

Figure S11. purity profile of Caftaric acid (**3**).

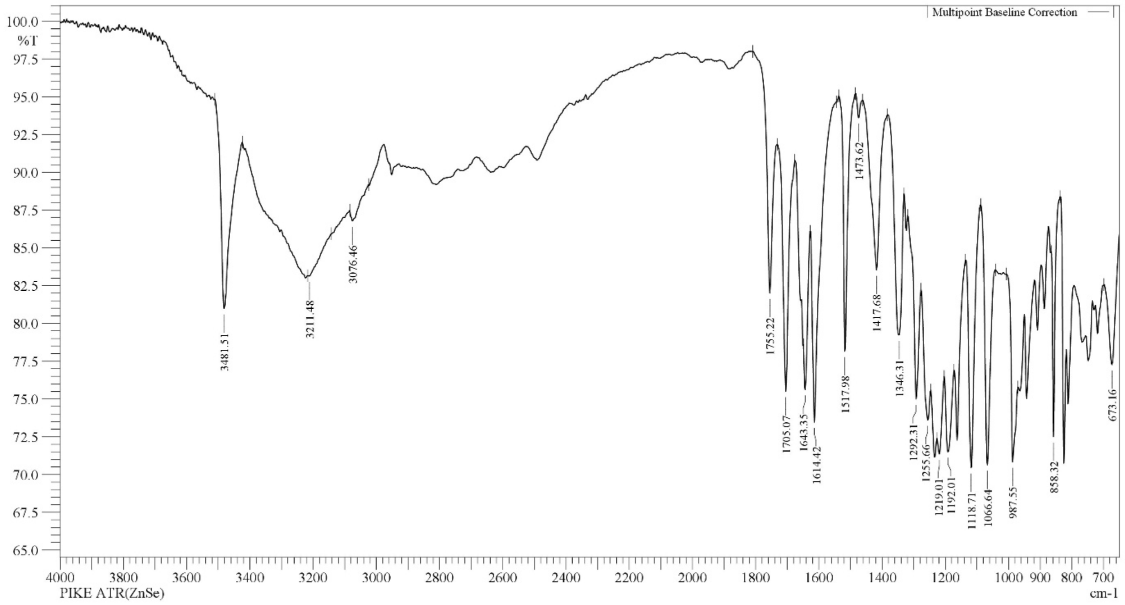

Figure S12. IR spectrum of Caftaric acid (**3**).

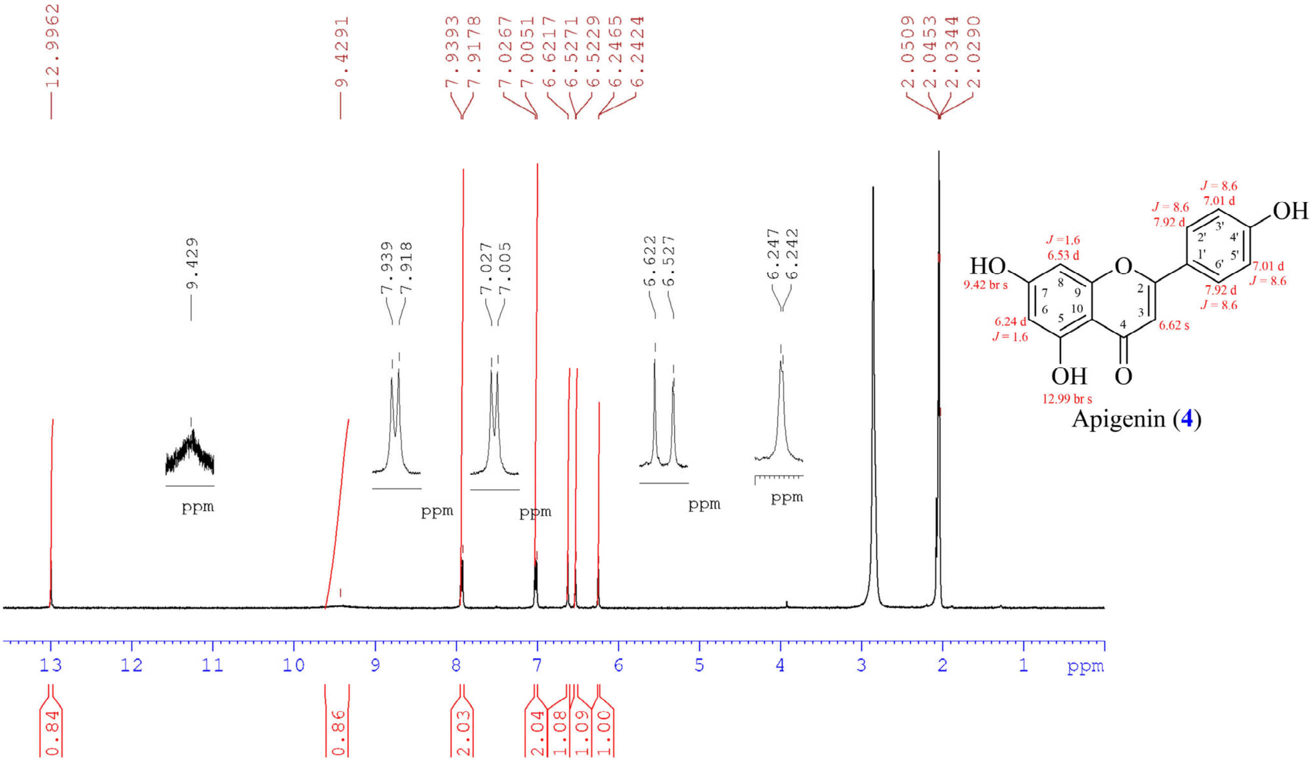

Figure S13. ^{1}H-NMR spectrum of Apigenin (**4**).

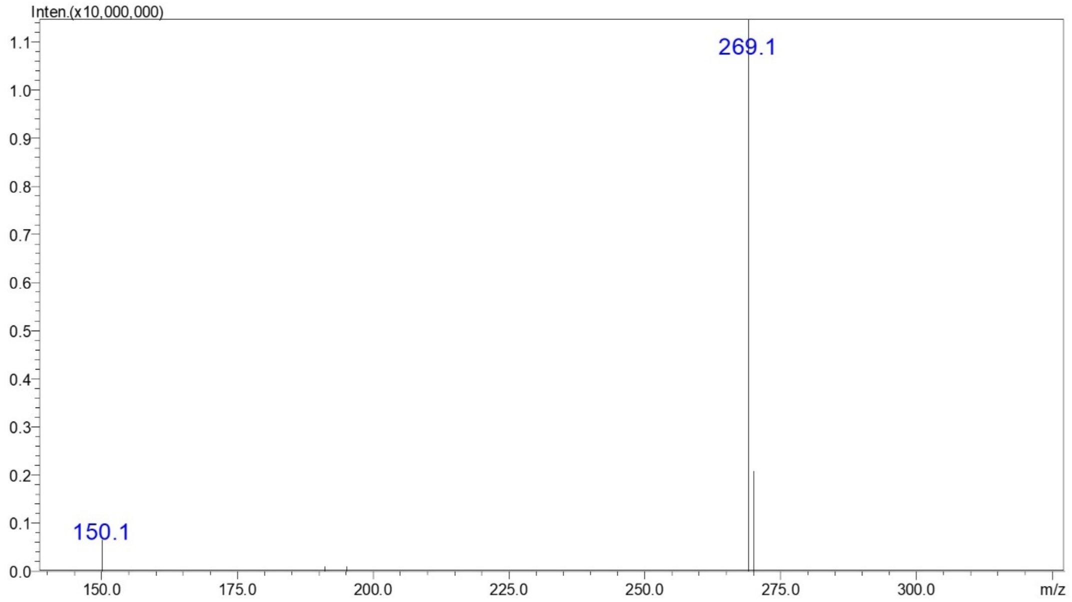

Figure S14. ESI-MS spectrum of Apigenin (**4**).

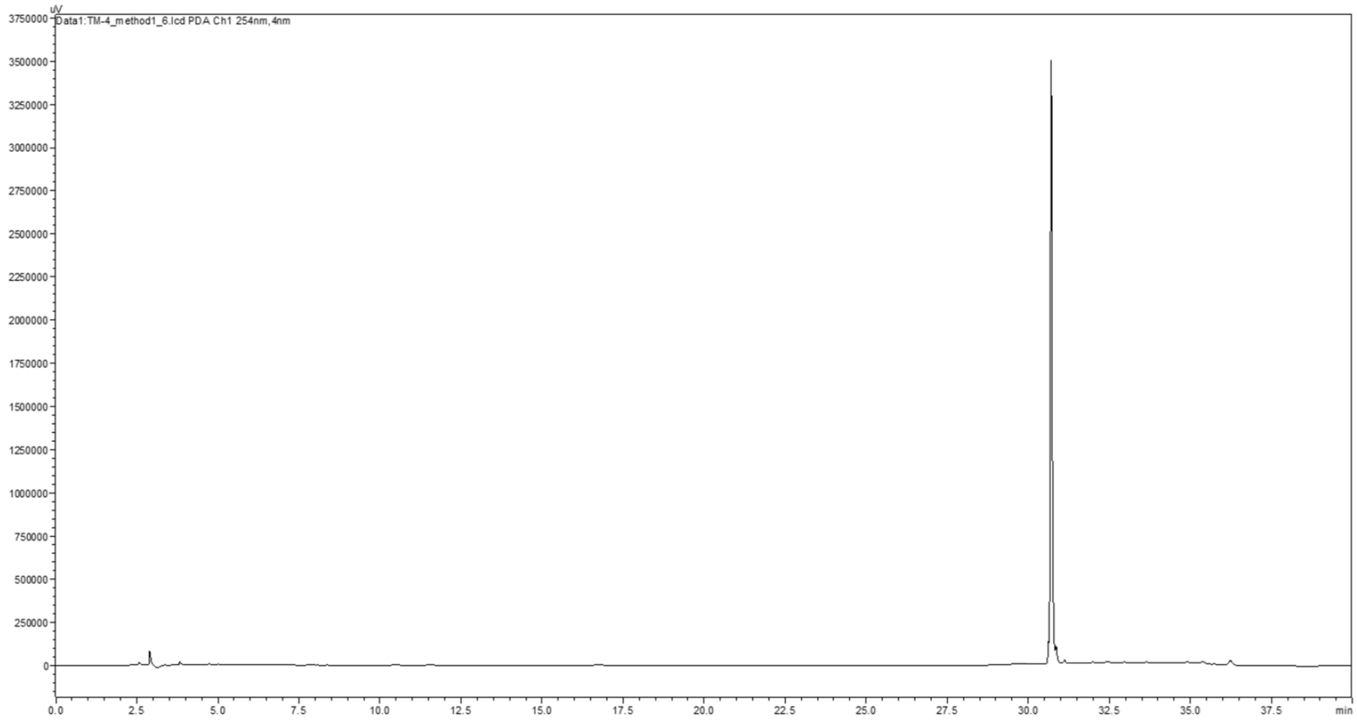

Peak#	Ret. Time	Area	Height	Mark	Conc.	Area%
1	30.086	127970	23761	M	0.000	0.850
2	30.709	14618151	3492665	M	0.000	97.112
3	34.897	45432	8864	M	0.000	0.302
4	35.368	69679	6604	M	0.000	0.463
5	36.233	191591	24822	M	0.000	1.273
Total		15052823	3556716		0.000	100.000

Figure S15. purity profile of Apigenin (**4**).

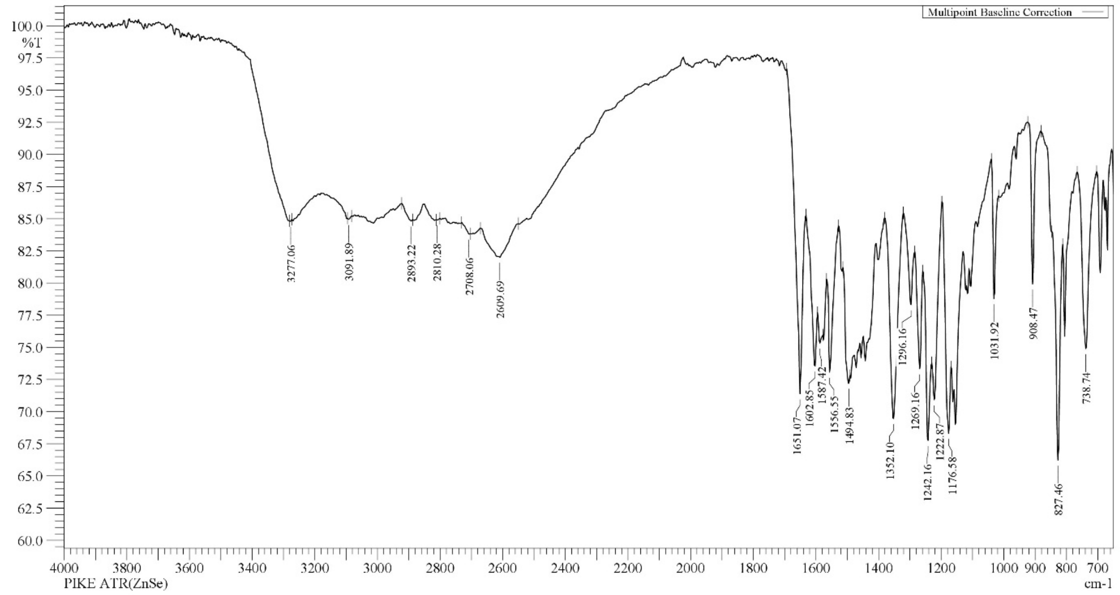

Figure S16. IR spectrum of Apigenin (**4**).

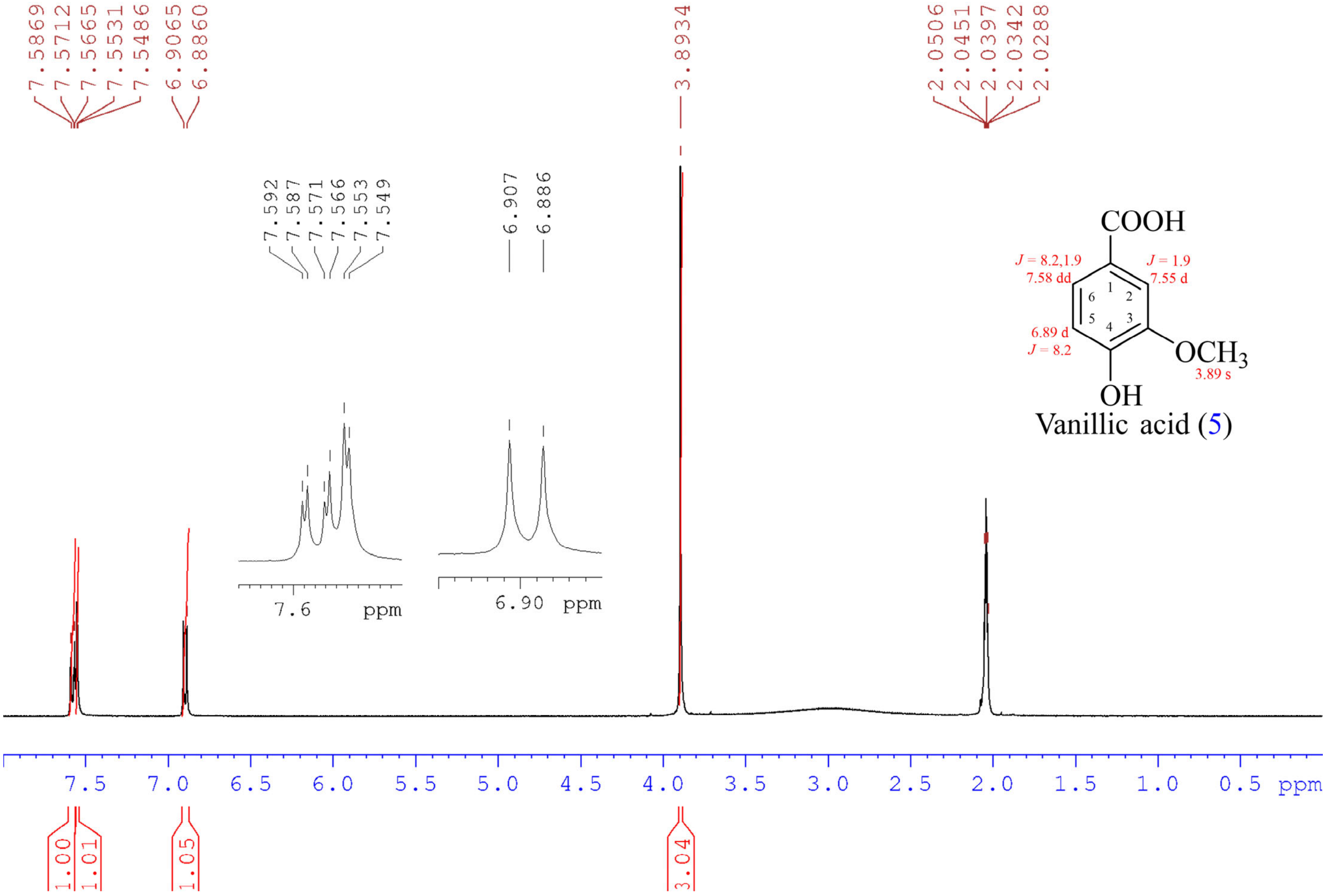

Figure S17. ^{1}H-NMR spectrum of Vanillic acid (**5**).

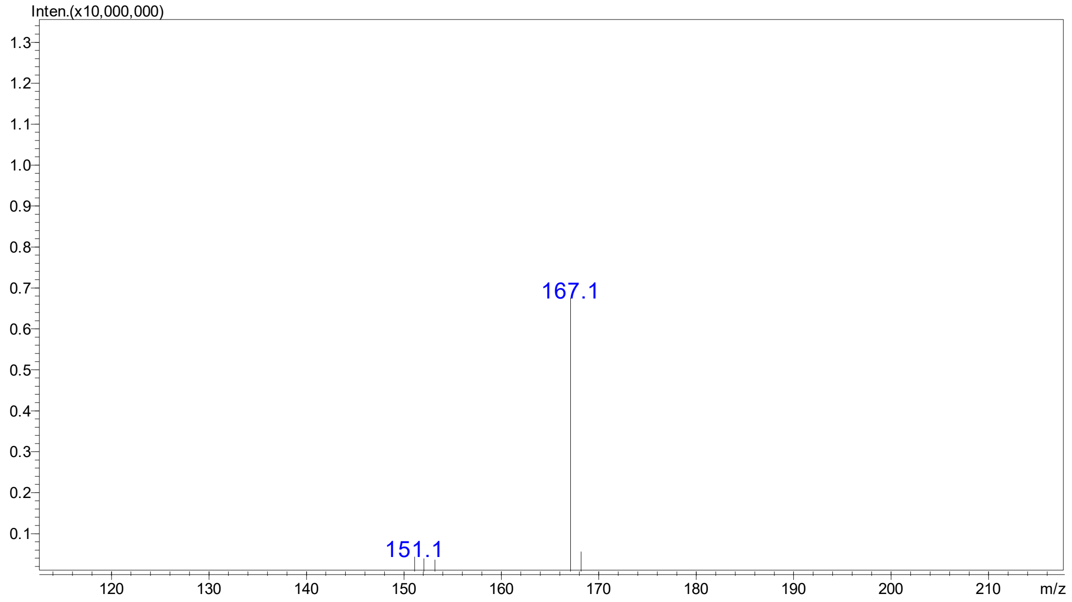

Figure S18. ESI-MS spectrum of Vanillic acid (**5**).

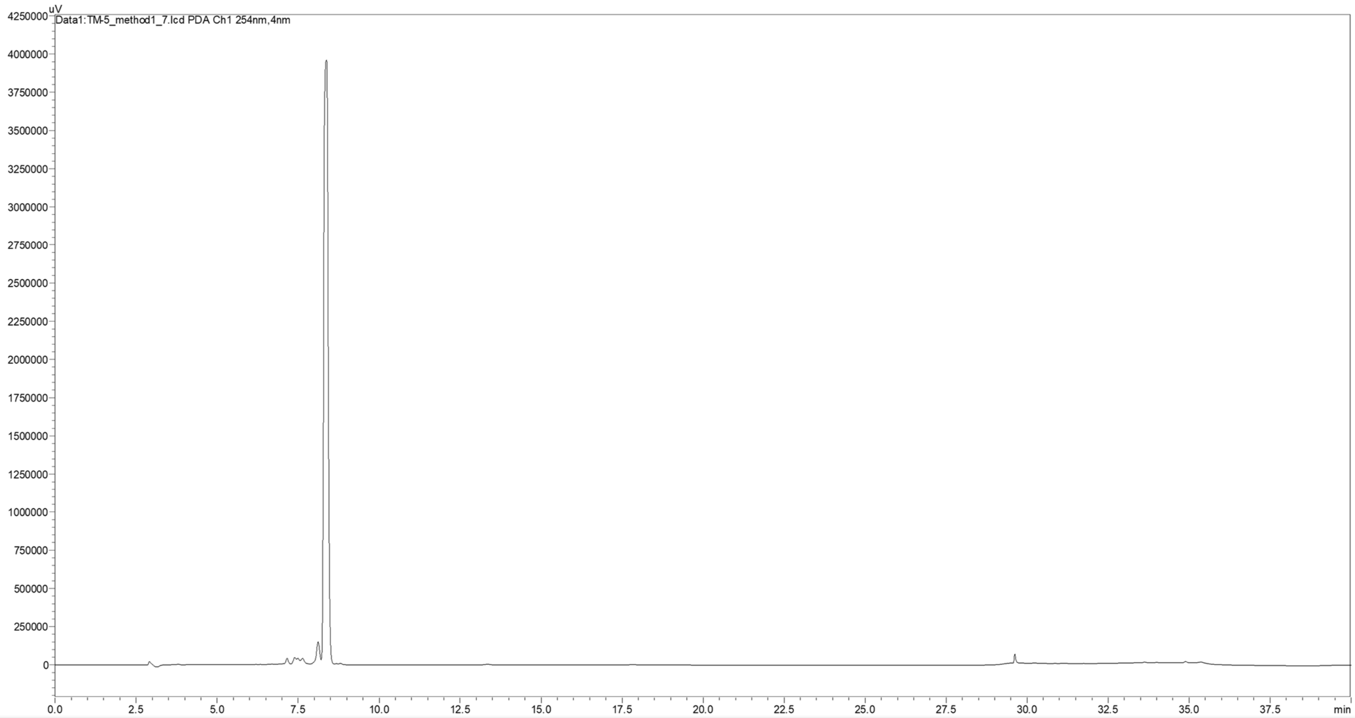

Peak Table | Compound | Group | Calibration Curve

Peak#	Ret. Time	Area	Height	Mark	Conc.	Area%
1	7.393	357529	21793	M	0.000	0.967
2	8.116	745370	124355	M	0.000	2.016
3	8.371	35871295	3935750	M	0.000	97.017
Total		36974195	4081897		0.000	100.000

Figure S19. purity profile of Vanillic acid (**5**).

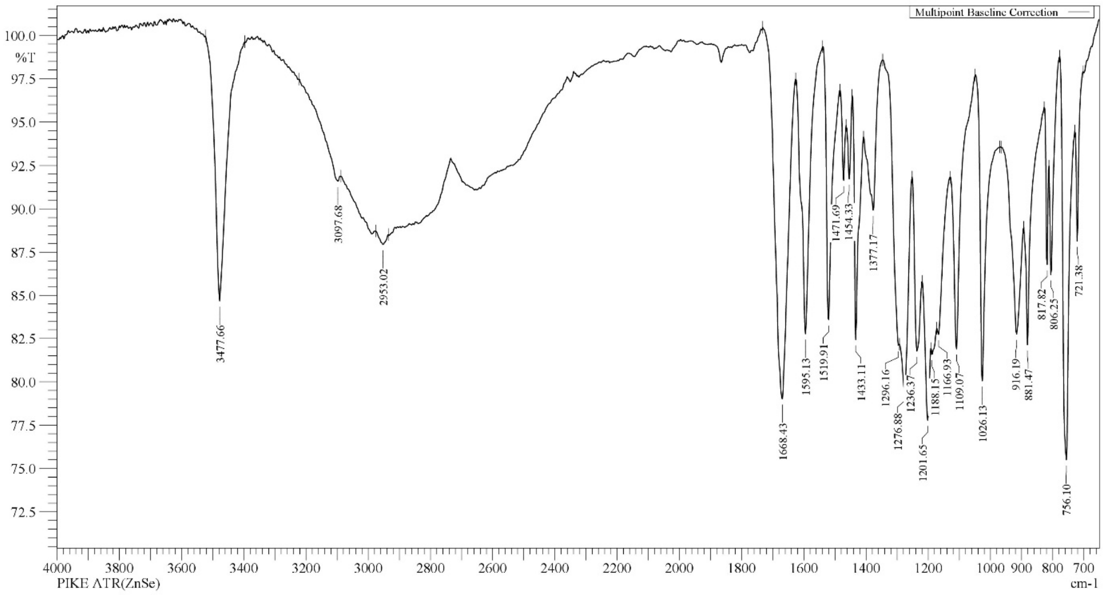

Figure S20. IR spectrum of Vanillic acid (**5**).

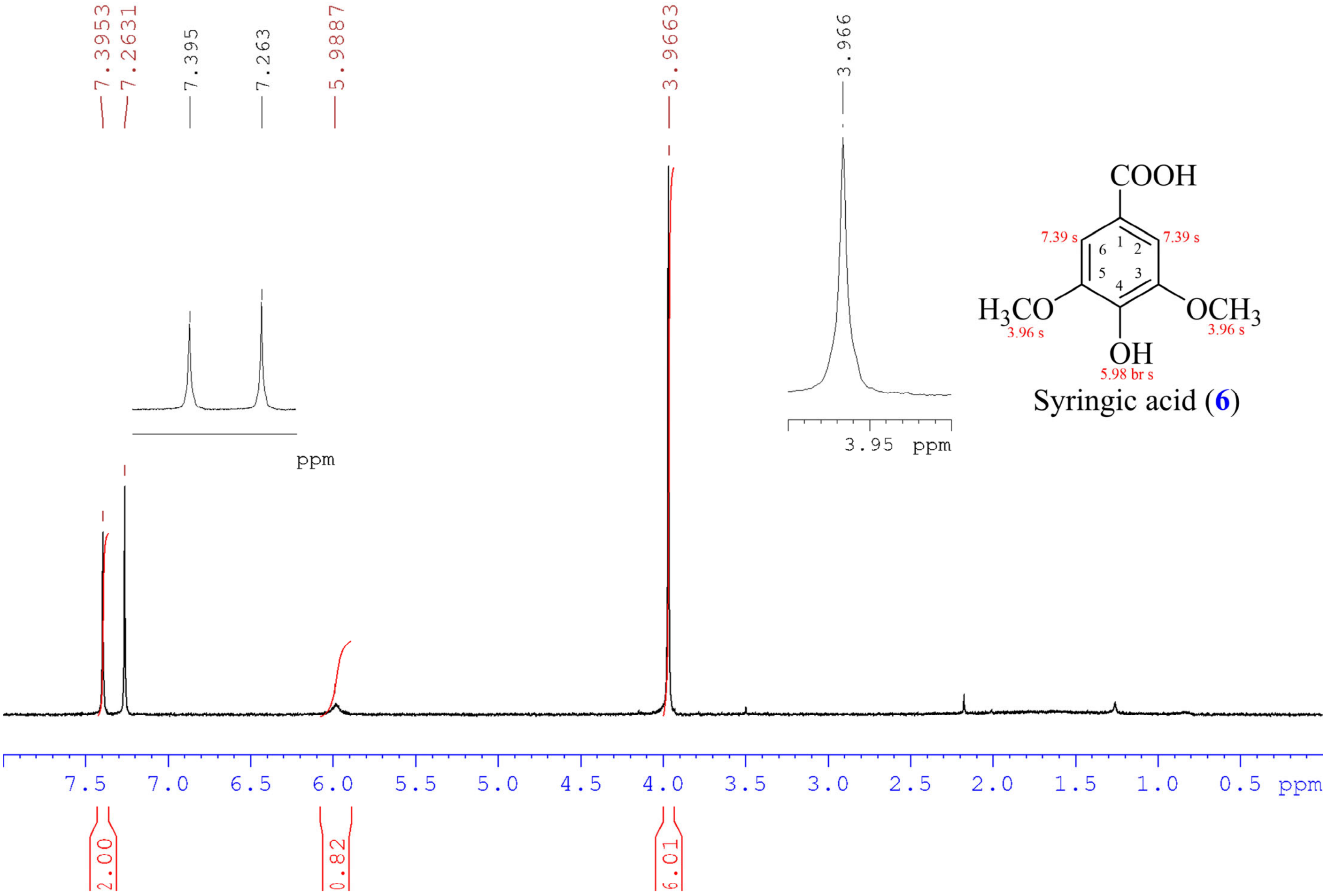

Figure S21. ^{1}H-NMR spectrum of Syringic acid (**6**).

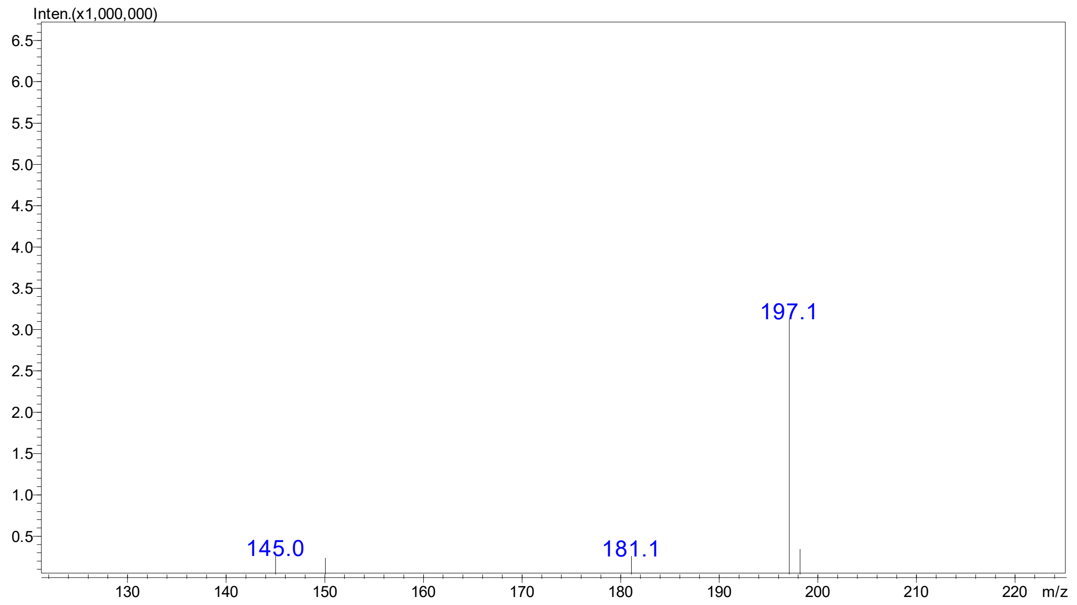

Figure S22. ESI-MS spectrum of Syringic acid (**6**).

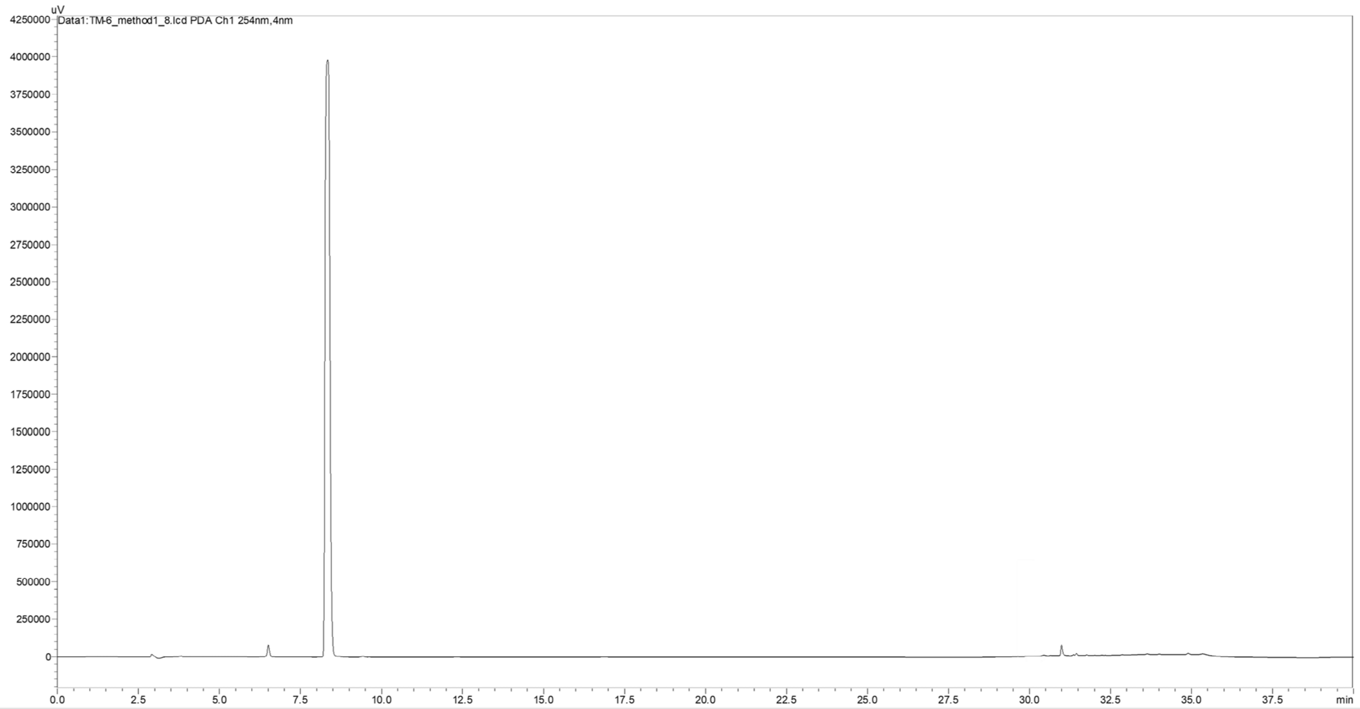

Results View - Peak Table

Peak Table | Compound | Group | Calibration Curve

Peak#	Ret. Time	Area	Height	Mark	Conc.	Area%
1	2.904	130285	21011	M	0.000	0.320
2	6.509	376743	77755	M	0.000	0.925
3	8.339	39776778	3979006	M	0.000	97.647
4	9.418	31608	4081	M	0.000	0.078
5	30.990	248763	68831	M	0.000	0.611
6	31.449	84194	15614	M	0.000	0.207
7	31.767	9922	4168	M	0.000	0.024
8	33.632	34281	5309	M	0.000	0.084
9	34.897	42836	8587	M	0.000	0.105
Total		40735410	4184361		0.000	100.000

Figure S23. purity profile of Syringic acid (**6**).

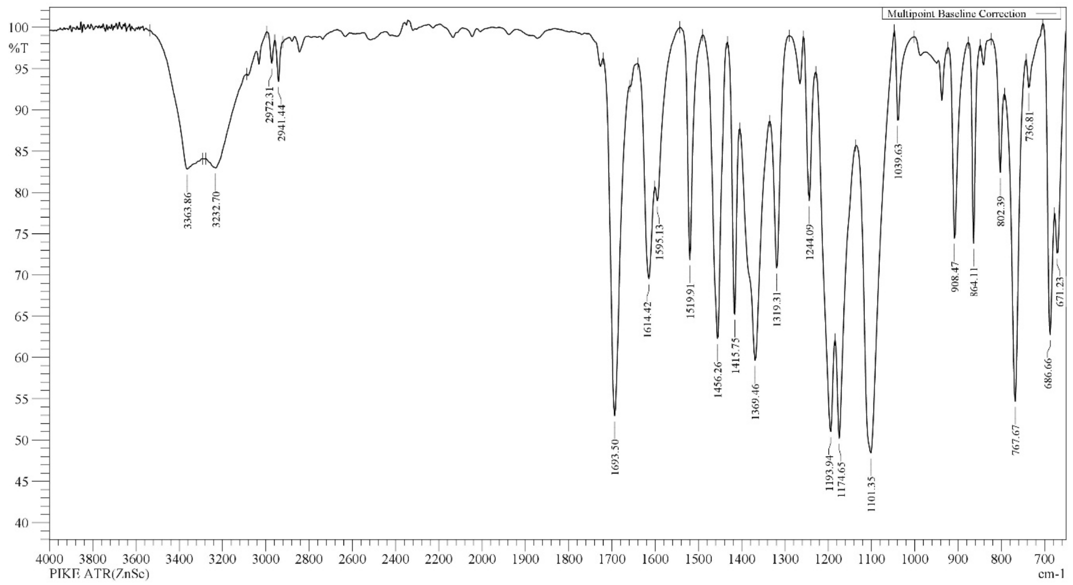

Figure S24. IR spectrum of Syringic acid (**6**).

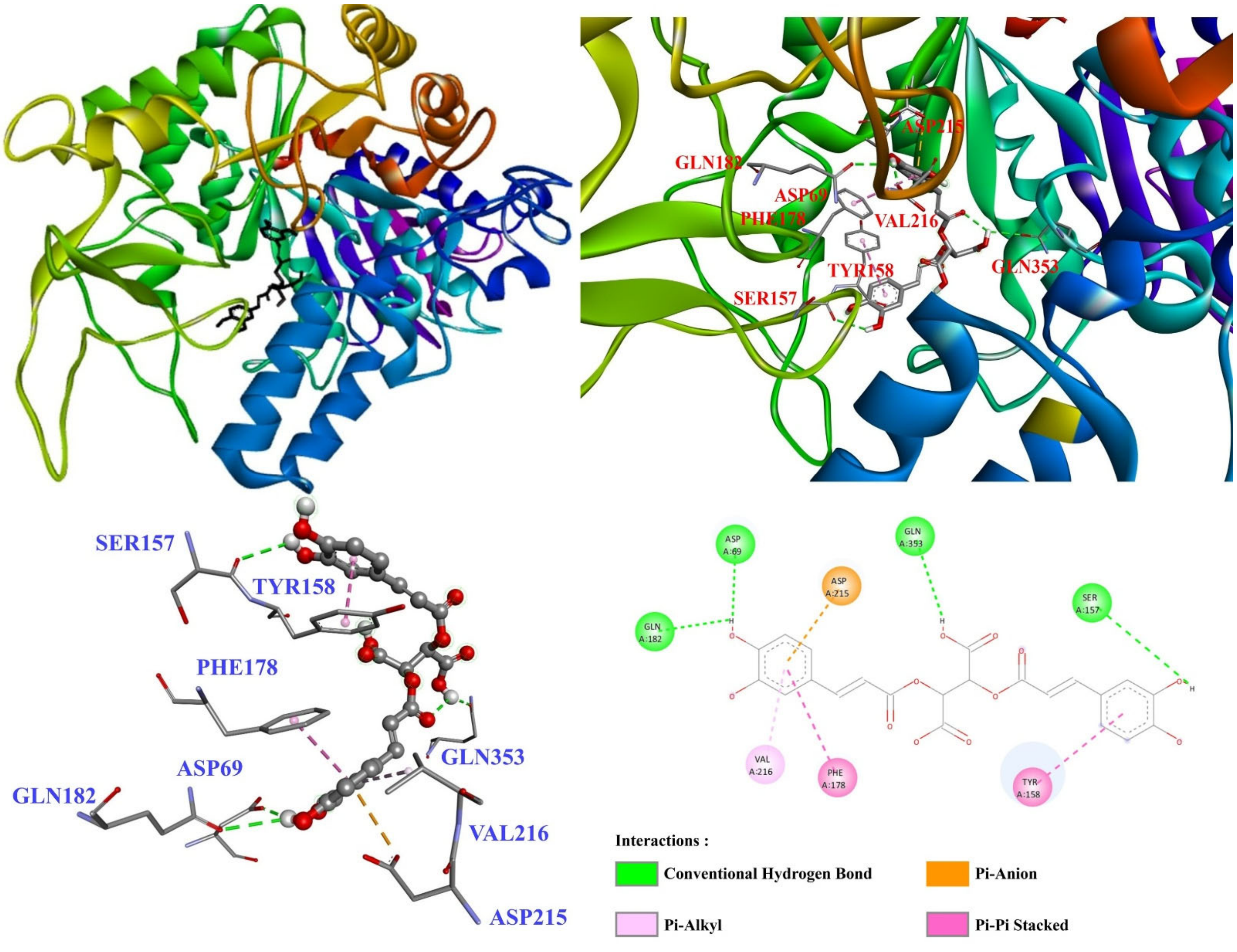

Figure S25. Molecular docking interactions of Chicoric acid with α-glucosidase

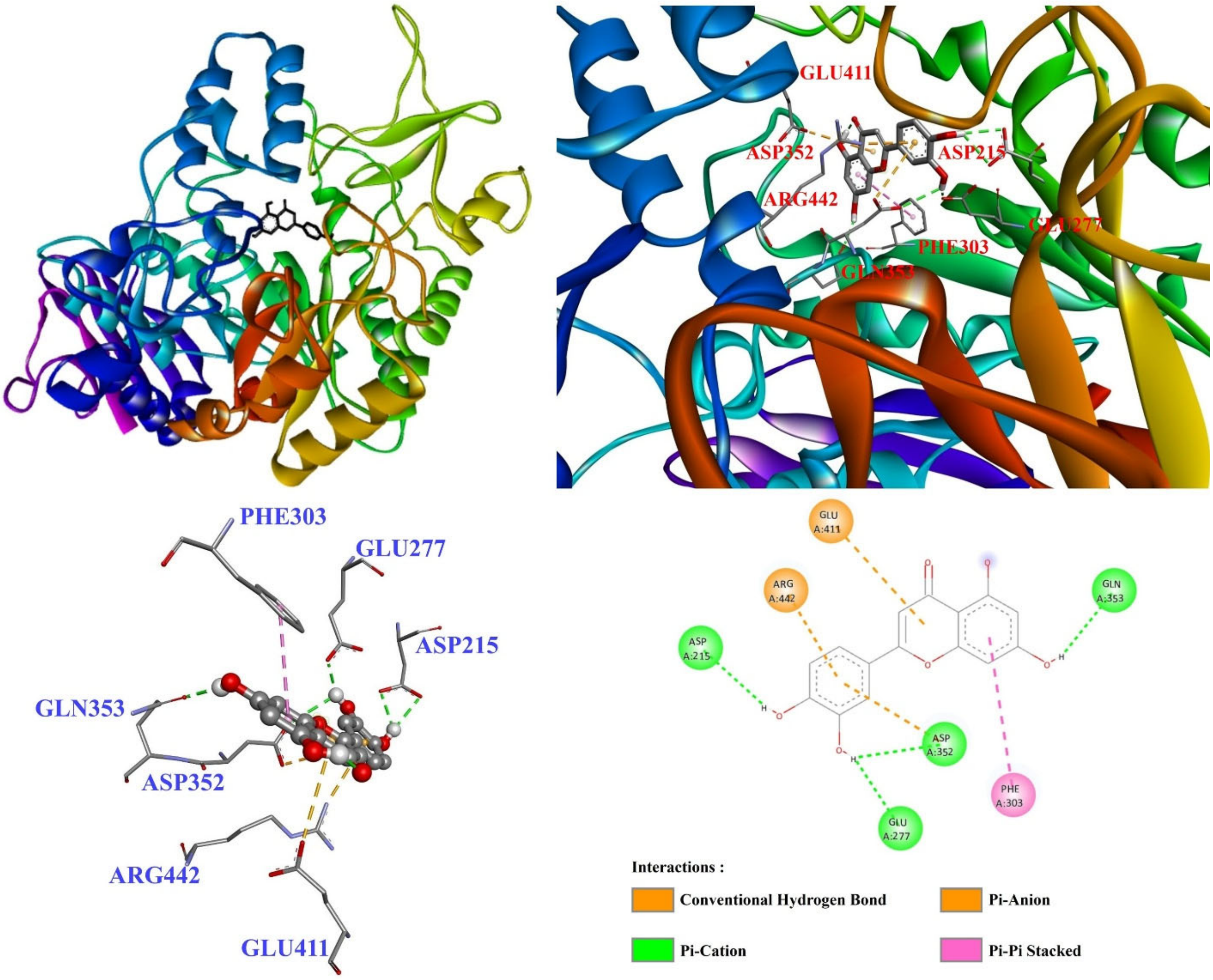

Figure S26. Molecular docking interactions of Luteolin with α-glucosidase

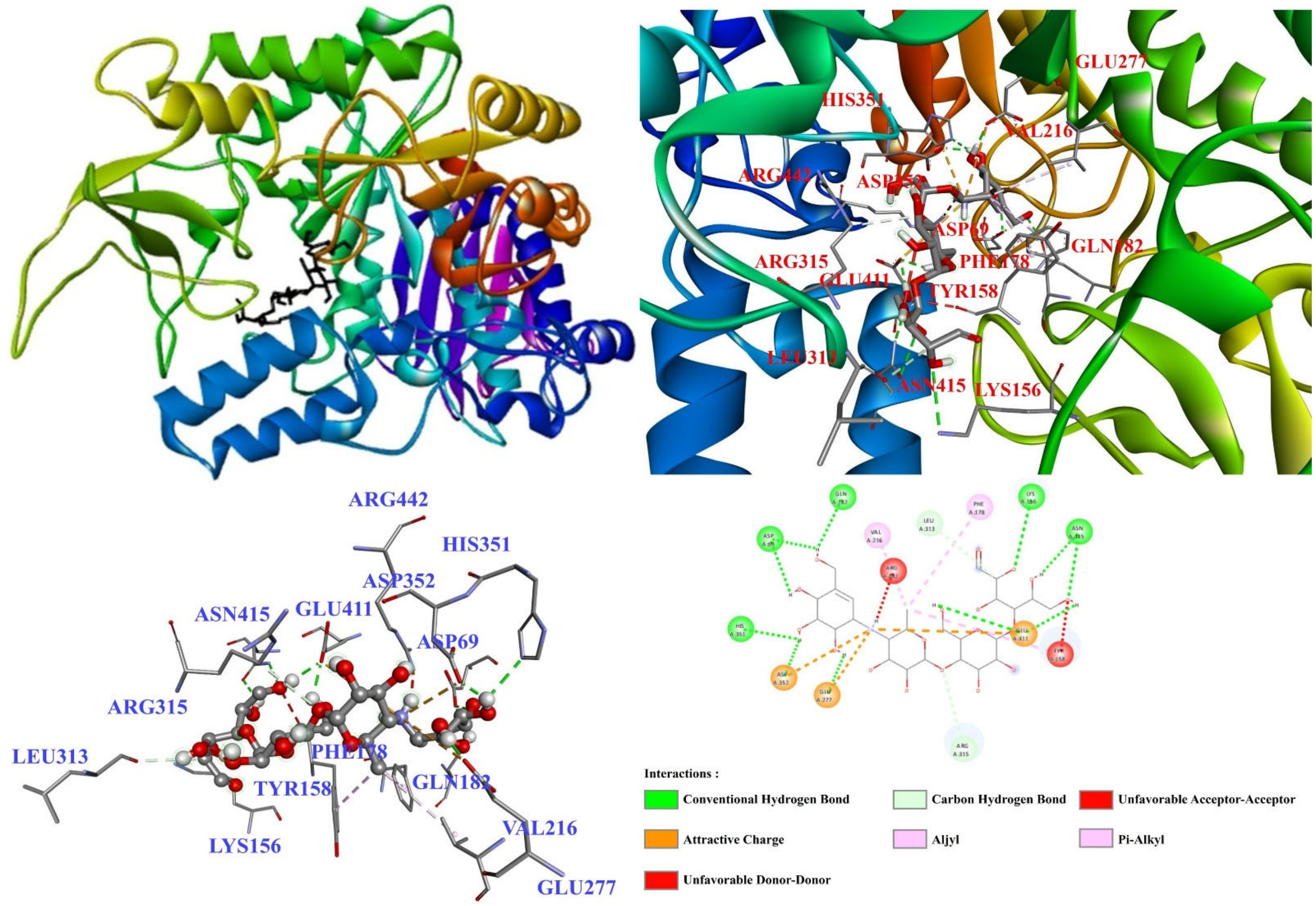

Figure S27. Molecular docking interactions of Acarbose with α-glucosidase

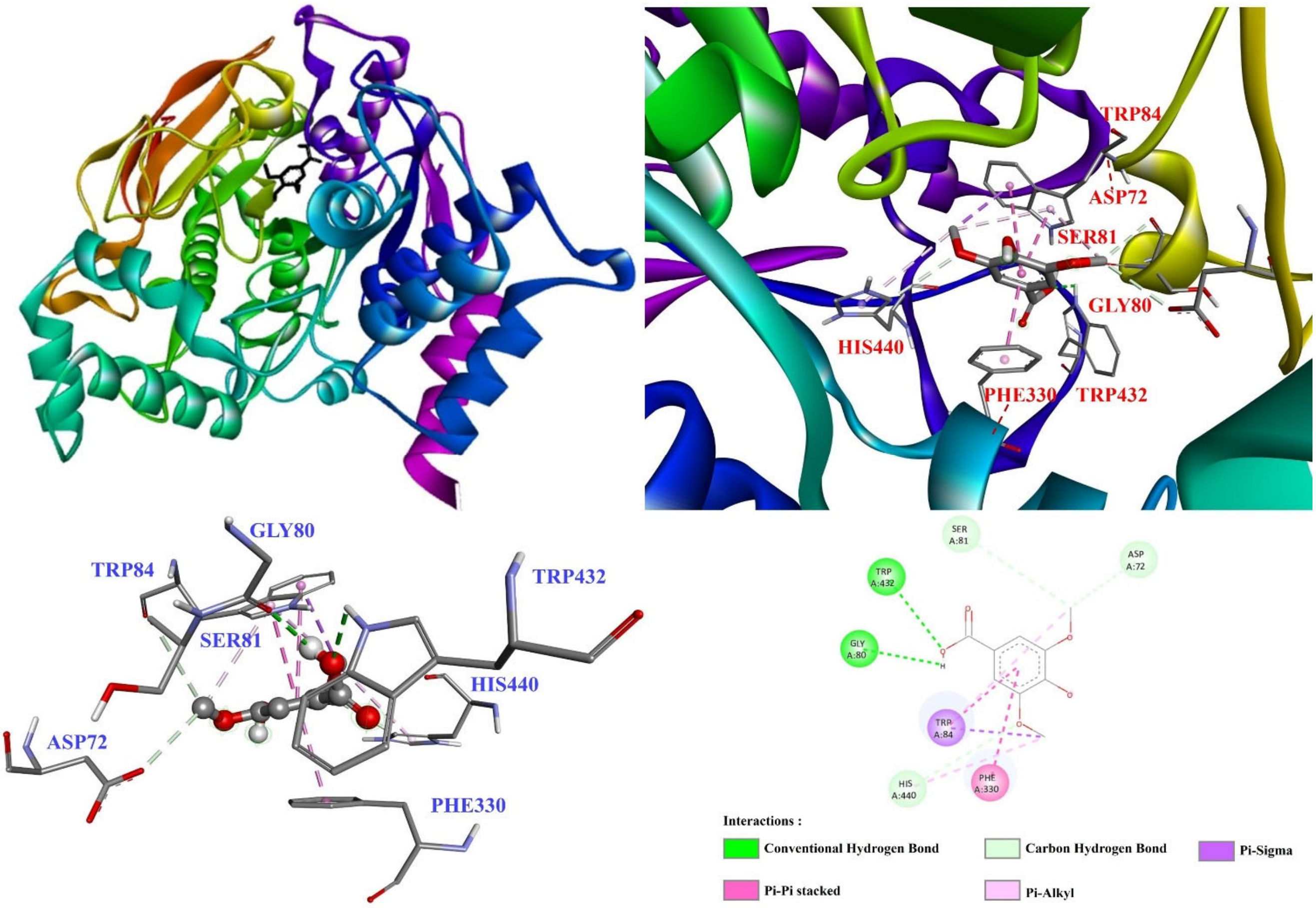

Figure S28. Molecular docking interactions of Syringic acid with AChE

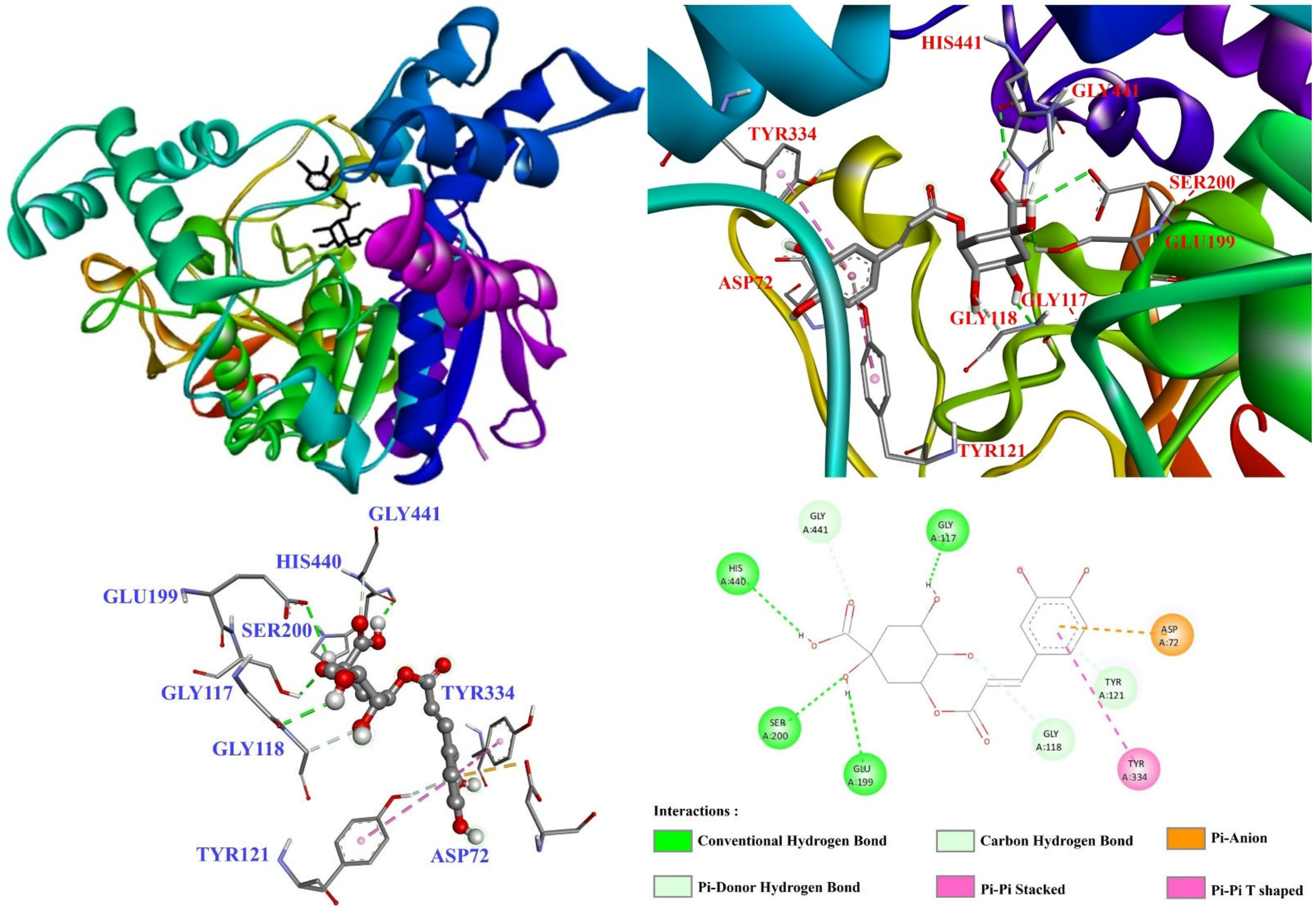

Figure S29. Molecular docking interactions of Cholorgenic acid with AchE

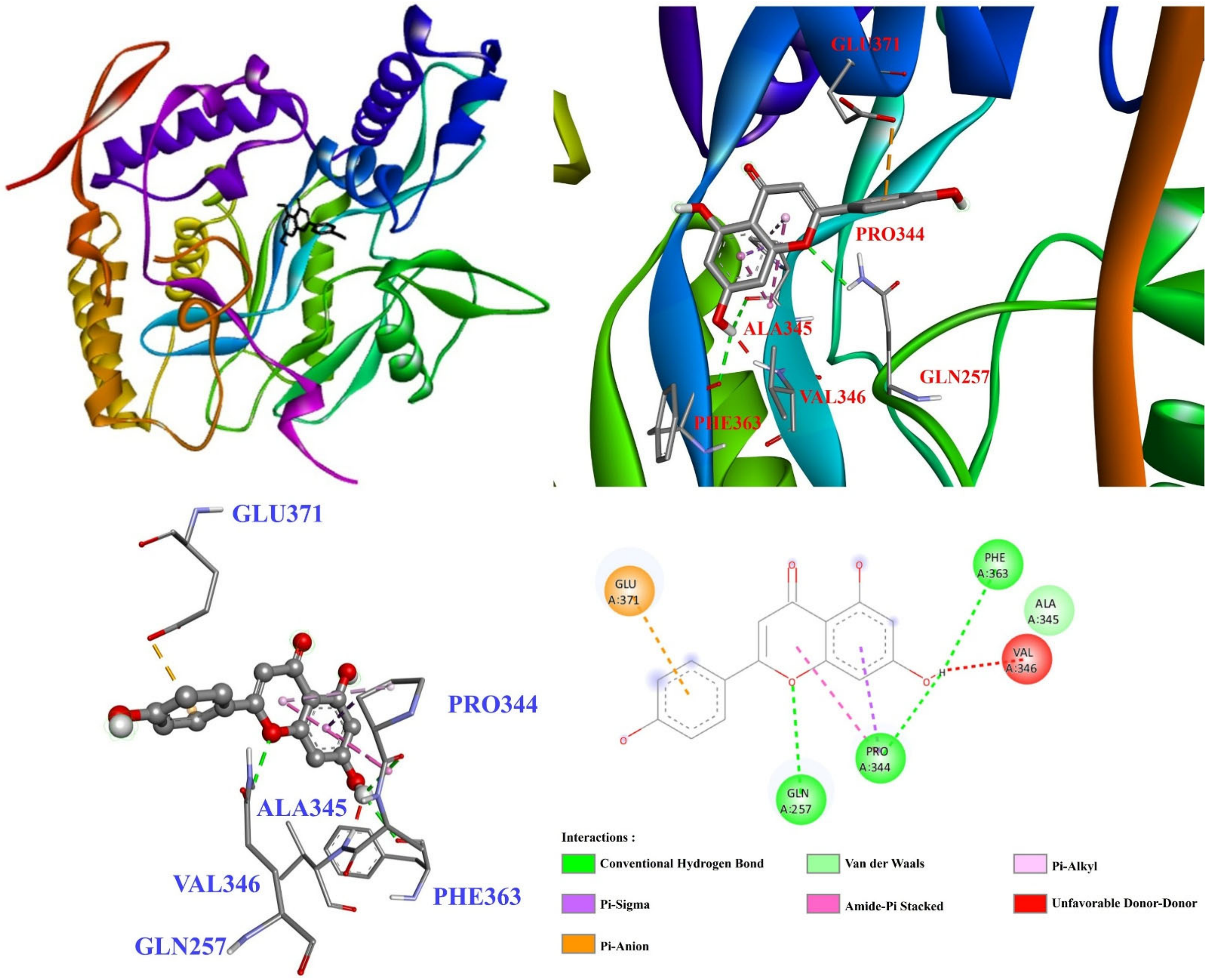

Figure S30. Molecular docking interactions of Apigenin with iNOS

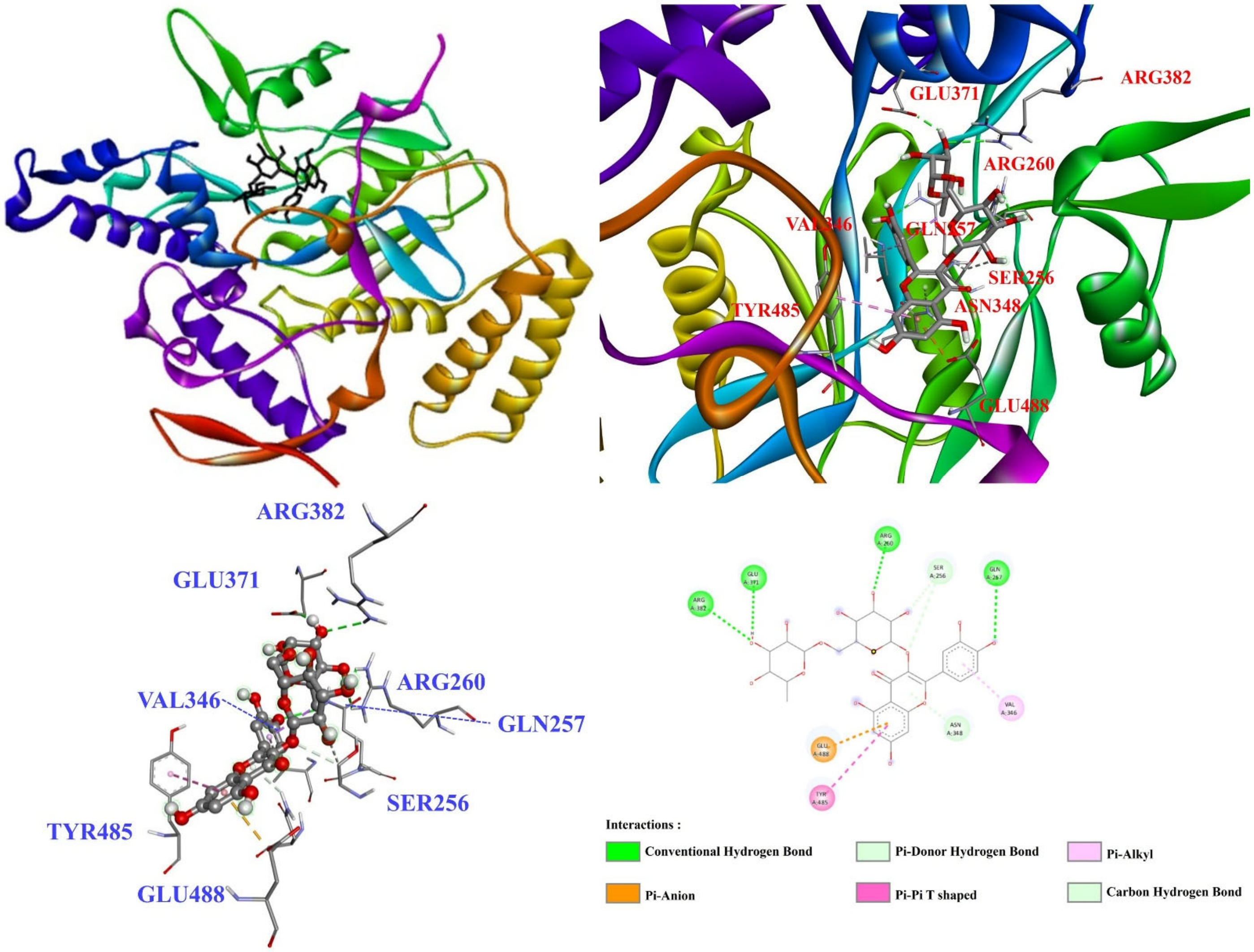

Figure S31. Molecular docking interactions of Rutin with iNOS

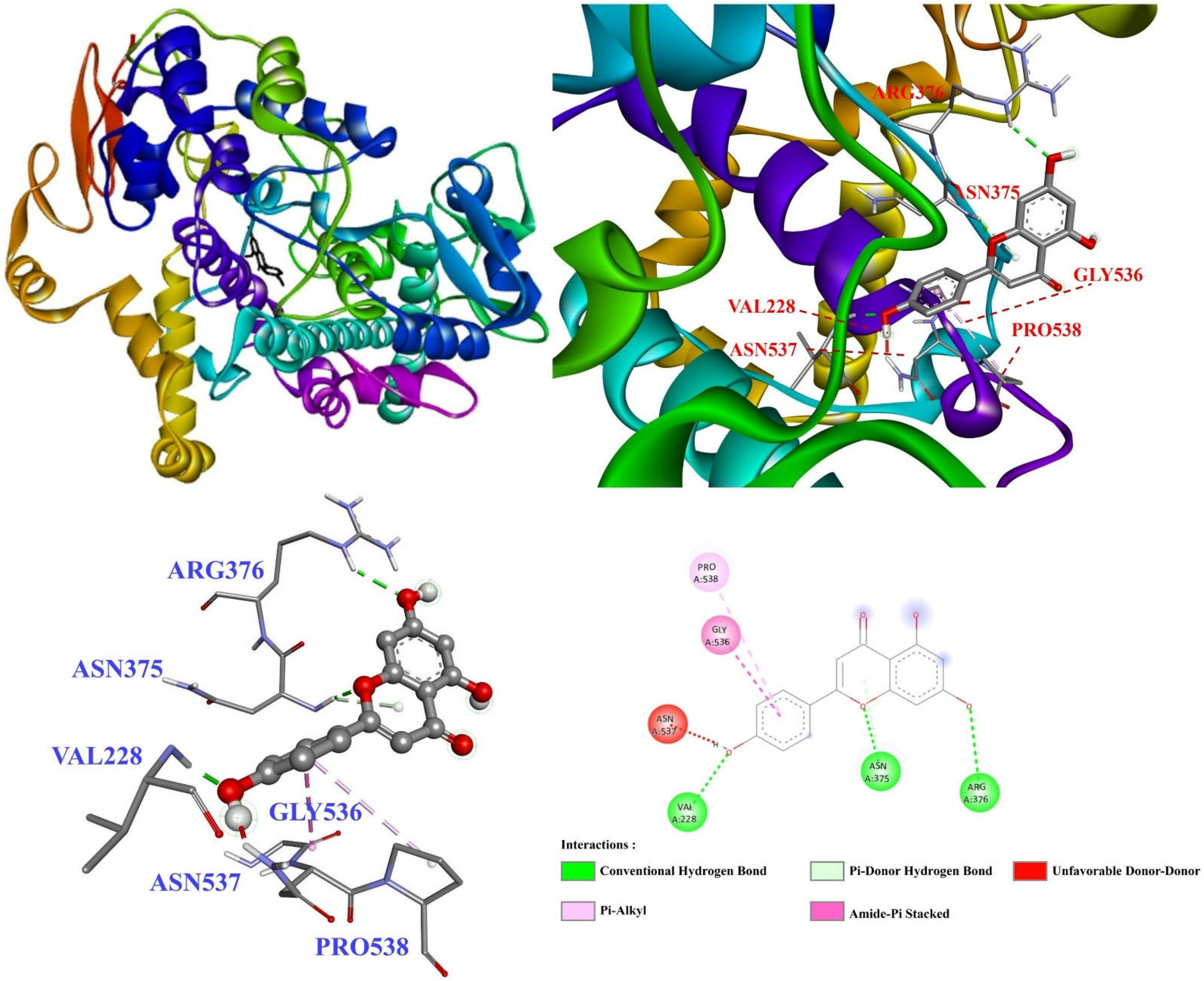

Figure S32. Molecular docking interactions of Apigenin with COX-2

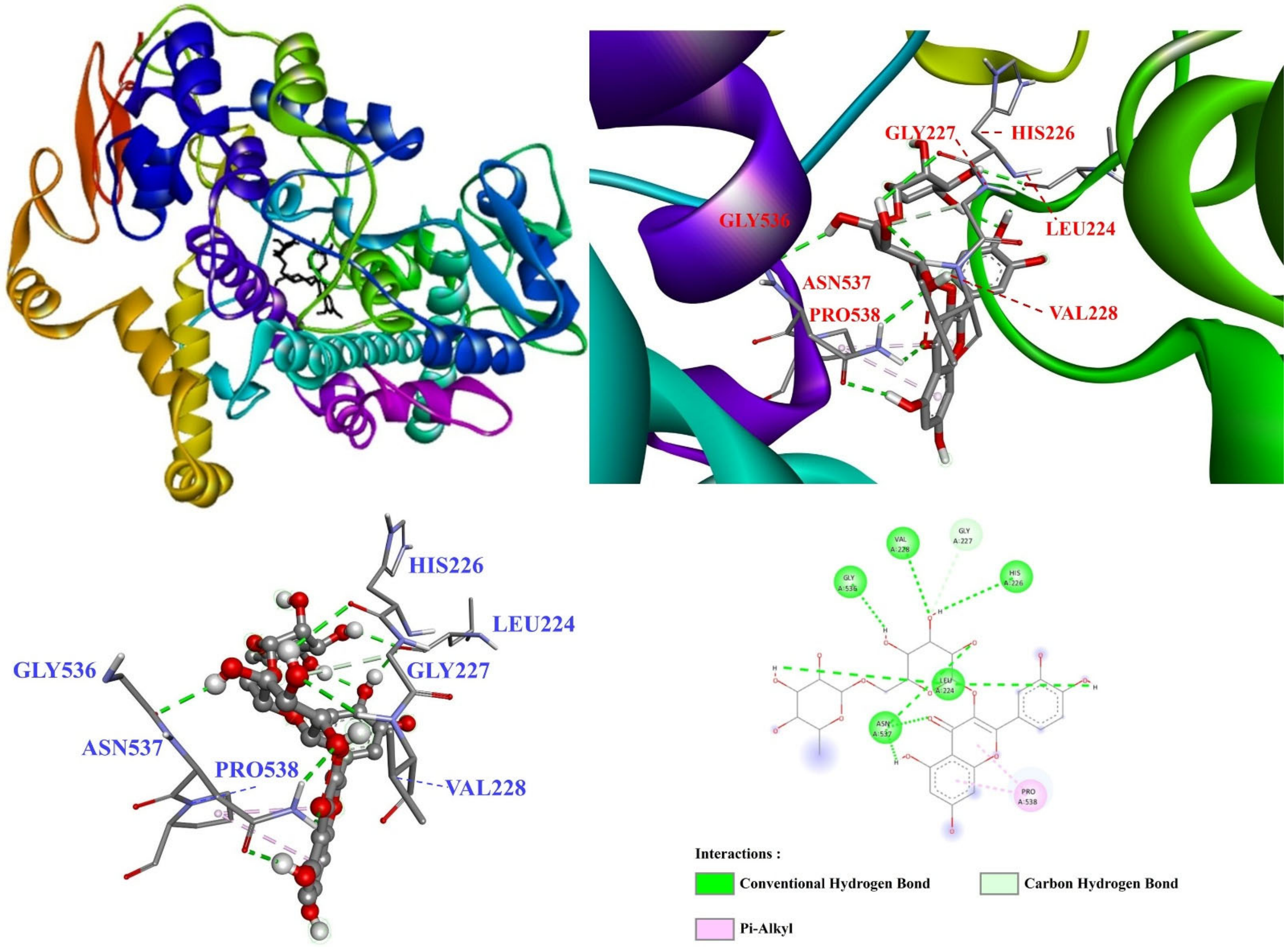

Figure S33. Molecular docking interactions of Rutin with COX-2

Primary antibody information

Table S1. Primary antibodies used for Western blot analysis.

Antibody	Host / Clonality	Supplier (Catalog No.)	Dilution
anti-β-actin	Rabbit polyclonal	GeneTex (GTX109639)	1:1000
anti-iNOS (D6B6S)	Rabbit monoclonal	Cell Signaling Technology (#13120)	1:1000
anti-COX-2	Rabbit monoclonal	Cell Signaling Technology (#12282)	1:1000
anti-TNF-α	Rabbit polyclonal	GeneTex (GTX110520)	1:1000
anti-IL-6	Rabbit polyclonal	GeneTex (GTX110527)	1:1000
anti-Phospho-SAPK/JNK (Thr183/Tyr185)	Rabbit polyclonal	Cell Signaling Technology (#4668)	1:1000
anti-SAPK/JNK	Rabbit polyclonal	Cell Signaling Technology (#9252)	1:1000
anti-Phospho-p38 MAPK (Thr180/Tyr182)	Rabbit polyclonal	Cell Signaling Technology (#9216)	1:1000
anti-p38 MAPK	Rabbit polyclonal	Cell Signaling Technology (#9212)	1:1000
anti-Phospho-p44/42 MAPK (ERK1/2) (Thr202/Tyr204)	Rabbit polyclonal	Cell Signaling Technology (#9101)	1:1000
anti-p44/42 MAPK (ERK1/2)	Rabbit polyclonal	Cell Signaling Technology (#9102)	1:1000
anti-IκBα	Rabbit polyclonal	Cell Signaling Technology (#9242)	1:1000
anti-Phospho-IκBα	Rabbit polyclonal	Cell Signaling Technology (#2859)	1:1000
anti-Arginase-1	Rabbit polyclonal	Cell Signaling Technology (#9819)	1:1000
anti-KLF4	Rabbit polyclonal	Cell Signaling Technology (#4038)	1:1000

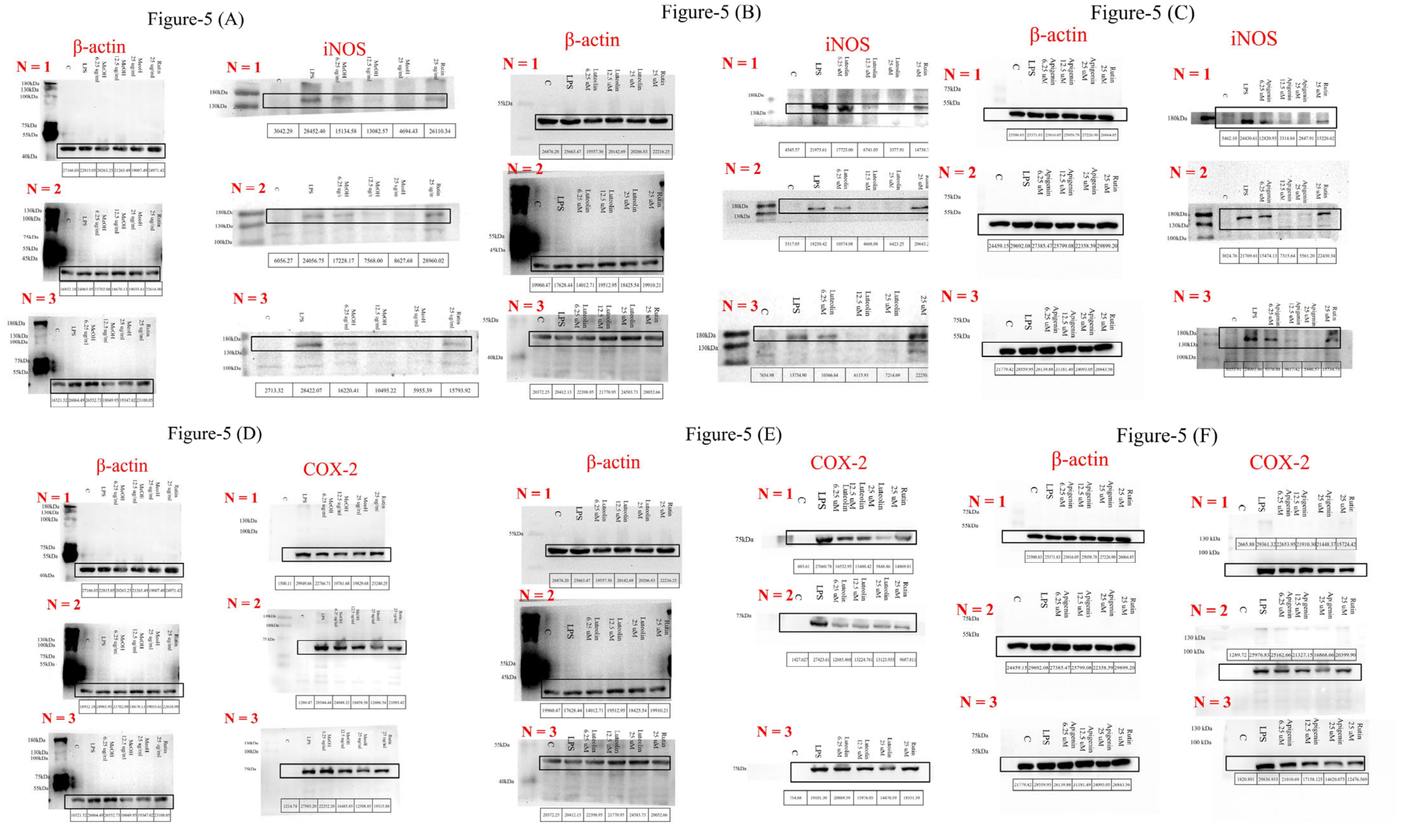

Figure S34. Original uncropped Western blot images corresponding to Figure 5A–F.

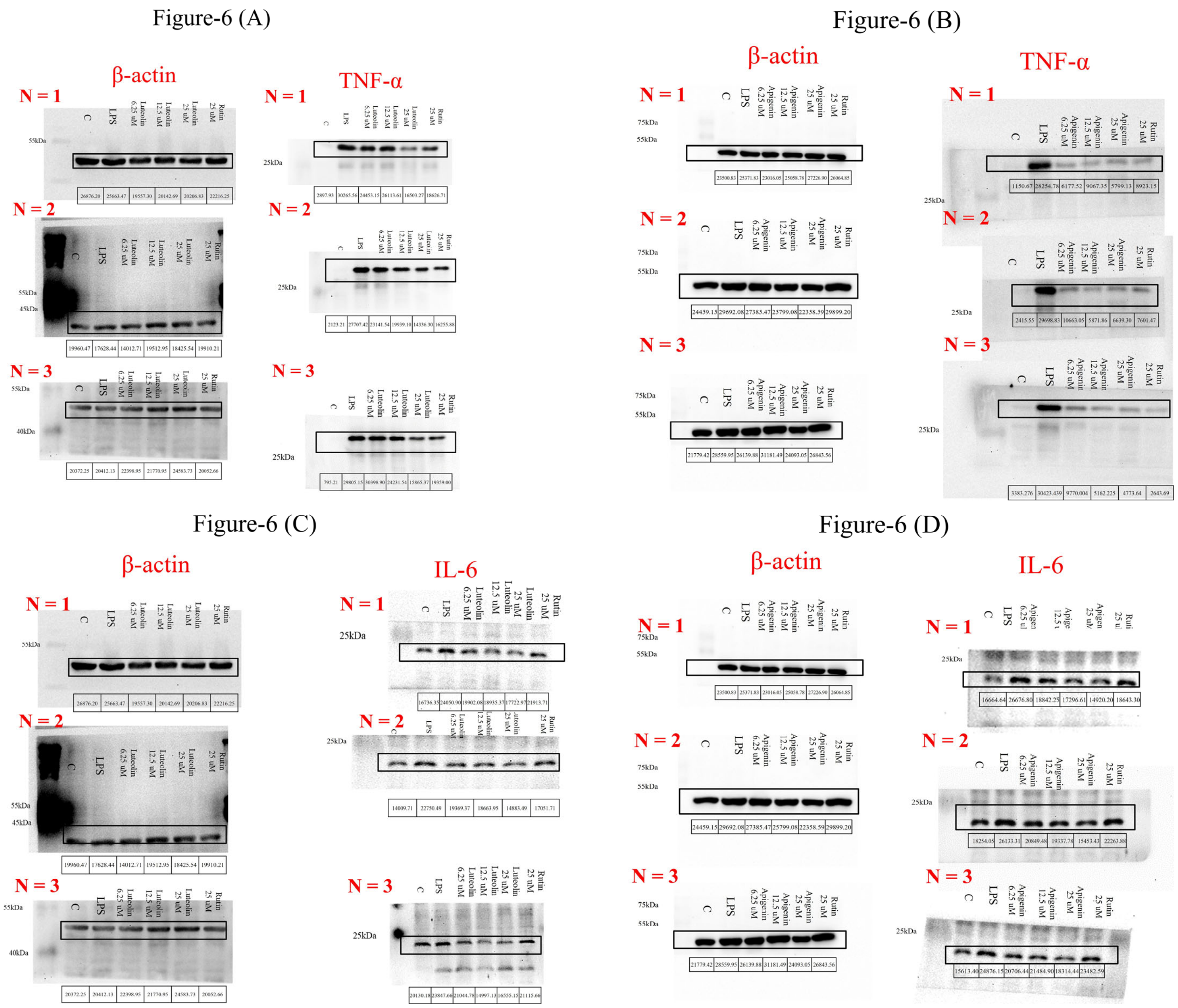

Figure S35. Original uncropped Western blot images corresponding to Figure 6A–D.

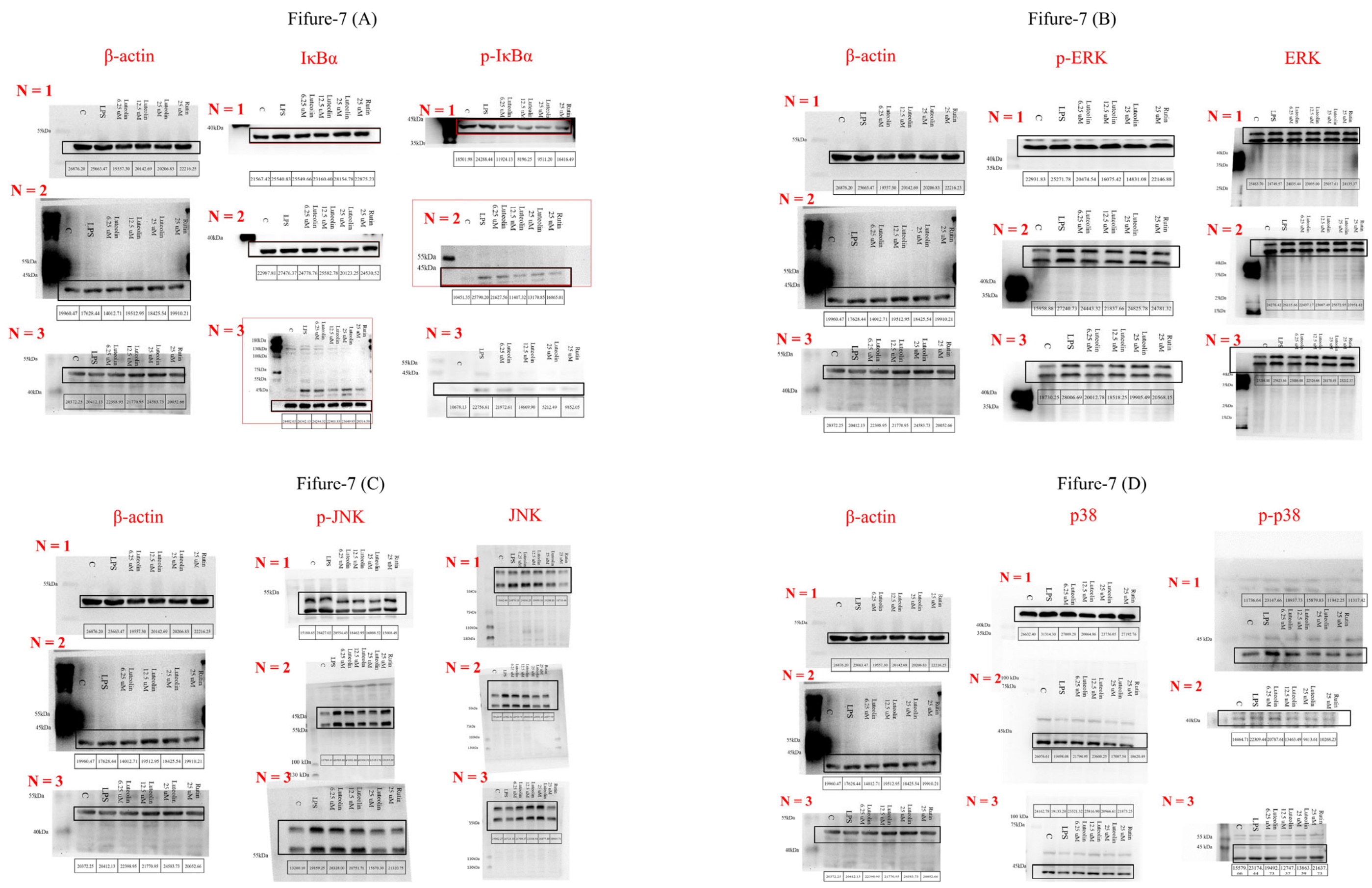

Figure S36. Original uncropped Western blot images corresponding to Figure 7A–D.

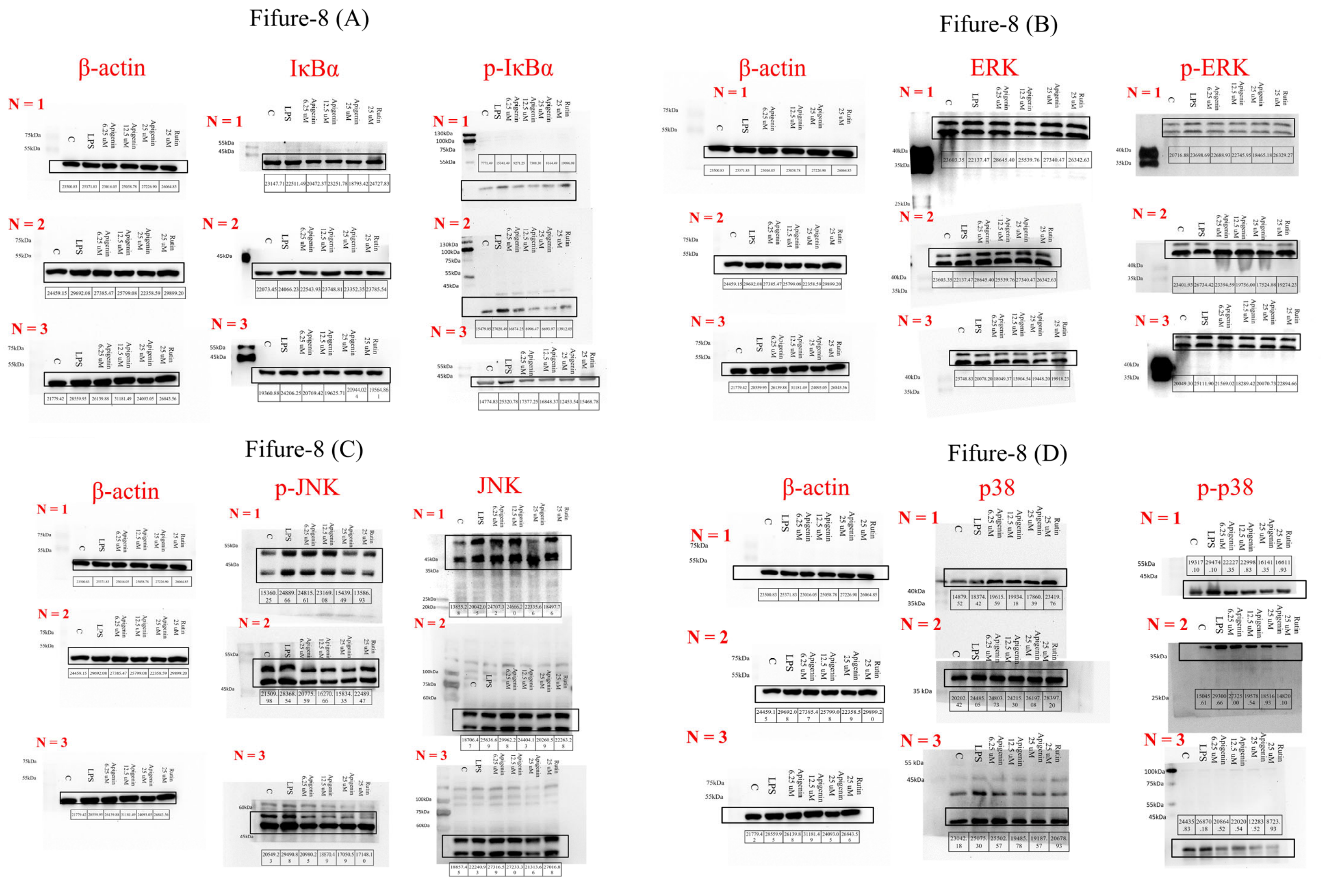

Figure S37. Original uncropped Western blot images corresponding to Figure 8A–D.

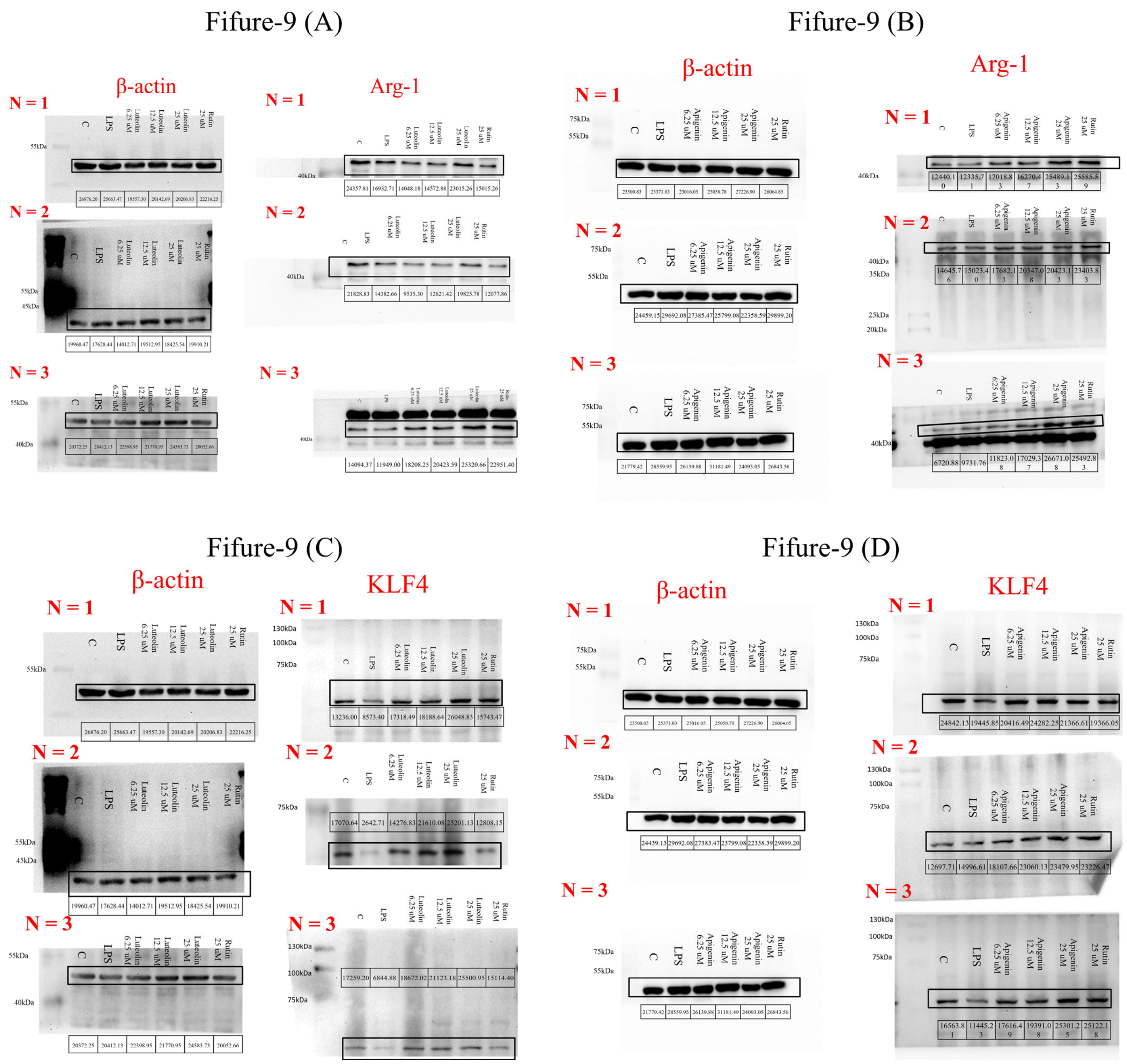

Figure S38. Original uncropped Western blot images corresponding to Figure 9A–D.
